# Pharmacological modulation of conditioned fear in the fear-potentiated startle test: a systematic review and meta-analysis of animal studies

**DOI:** 10.1007/s00213-022-06307-1

**Published:** 2023-01-18

**Authors:** Lucianne Groenink, P. Monika Verdouw, Yulong Zhao, Freija ter Heegde, Kimberley E. Wever, Elisabeth Y. Bijlsma

**Affiliations:** 1 Division of Pharmacology, Utrecht Institute for Pharmaceutical Sciences, Utrecht University, Universiteitsweg 99, 3584 CG Utrecht, The Netherlands; 2 Department of Anaesthesiology, Pain and Palliative Medicine, Radboud Institute for Health Sciences, Radboud University Medical Center, Geert Grooteplein 10, 6525 GA Nijmegen, The Netherlands

**Keywords:** Fear conditioning, Fear learning, Contextual anxiety, Acoustic startle, Anxiety, Rats, Mice, Animal model, Pharmacology, Methodology

## Abstract

**Rationale and objectives:**

Fear conditioning is an important aspect in the pathophysiology of anxiety disorders. The fear-potentiated startle test is based on classical fear conditioning and over the years, a broad range of drugs have been tested in this test. Synthesis of the available data may further our understanding of the neurotransmitter systems that are involved in the expression of conditioned fear.

**Methods:**

Following a comprehensive search in Medline and Embase, we included 68 research articles that reported on 103 drugs, covering 56 different drug classes. The systematic review was limited to studies using acute, systemic drug administration in naive animals.

**Results:**

Qualitative data synthesis showed that most clinically active anxiolytics, but not serotonin-reuptake inhibitors, reduced cued fear. Anxiogenic drugs increased fear potentiation in 35% of the experiments, reduced fear potentiation in 29% of the experiments, and were without effect in 29% of the experiments. Meta-analyses could be performed for five drug classes and showed that benzodiazepines, buspirone, 5-HT_1A_ agonists, 5-HT_1A_ antagonists, and mGluR2,3 agonists reduced cued conditioned fear. The non-cued baseline startle response, which may reflect contextual anxiety, was only significantly reduced by benzodiazepines and 5-HT_1A_ antagonists. No associations were found between drug effects and methodological characteristics, except for strain.

**Conclusions:**

The fear-potentiated startle test appears to have moderate to high predictive validity and may serve as a valuable tool for the development of novel anxiolytics. Given the limited available data, the generally low study quality and high heterogeneity additional studies are warranted to corroborate the findings of this review.

**Supplementary Information:**

The online version contains supplementary material available at 10.1007/s00213-022-06307-1.

## Introduction

Anxiety disorders are highly frequent occurring disorders (Bandelow and Michaelis 2015). Treatment options, including cognitive behavioral therapy, pharmacotherapy, or a combination of both, have proven relatively successful (Baldwin et al. 2014). A substantial group of patients, however, appear unresponsive to treatment or suffer from side effects (Baldwin et al. 2014; Bandelow et al. 2017; Gloster et al. 2013; Otte 2011). In the last two decades, both industry and academia have invested considerably in drug development for the treatment of anxiety disorders (Griebel and Holmes 2013), but without apparent breakthroughs (Swinney and Anthony 2011). It has proven difficult to successfully translate results from preclinical research to clinical practice in the field of psychiatric disorders (Llovera and Liesz 2016; Pankevich et al. 2014).

**Fig. 1:**
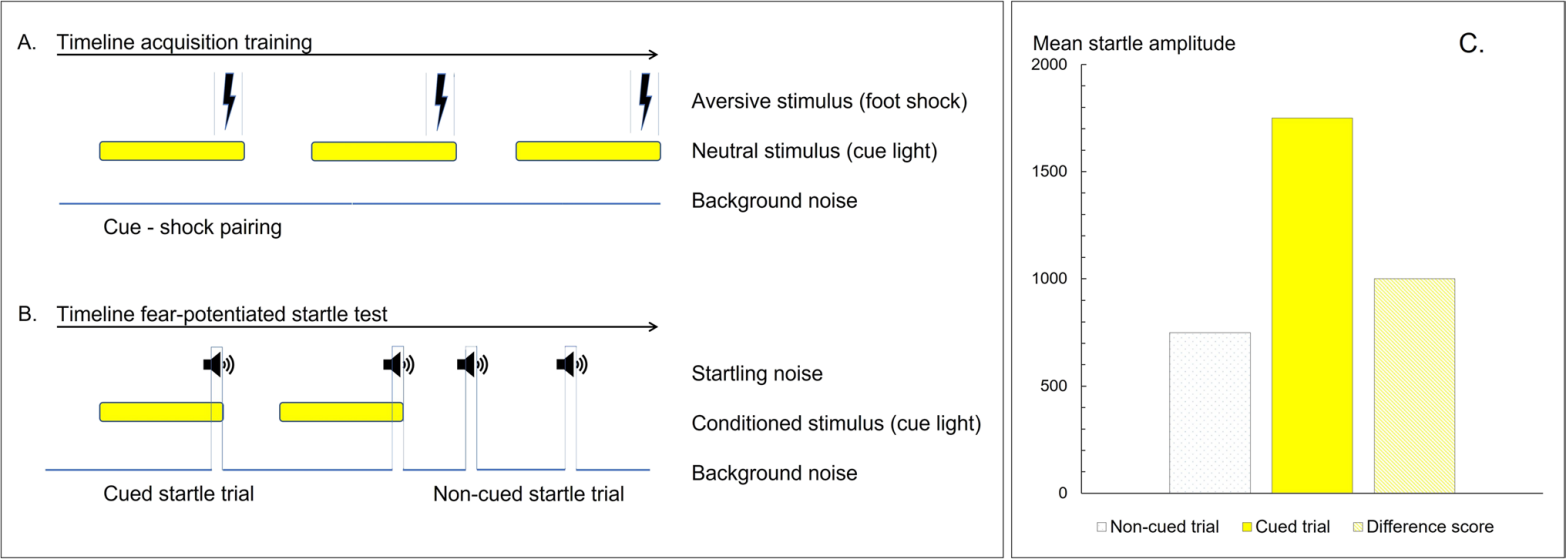
The fear-potentiated startle test procedure. The procedure consists of two phases and starts with an acquisition training (**A**), in which a neutral stimulus is paired with an unconditioned aversive stimulus, typically a foot shock. Upon repeated paired presentation of these stimuli, subjects learn to associate the cue with shock. This first phase is followed by the actual fear-potentiated startle test (**B**), which is typically conducted the day after the last training session. During the test session, acoustic stimuli are presented in the absence or presence of the conditioned stimulus. The magnitude of the startle response elicited in the presence of the conditioned stimulus (cued trial, **C**) relative to the response in absence of the conditioned stimulus (non-cued trial, **C**, white dotted bar) is taken as an index of cued-conditioned fear and can be used to measure the anxiolytic effect of the administered drug (Groenink et al. 2008). The magnitude of the startle response in the absence of the conditioned stimulus (non-cued trial, **C**, white bar) is considered the baseline response and is used to control for potential adverse drug effects, such as sedation and motor effects. Importantly though, a drug-induced reduction in the non-cued baseline startle response may also reflect a decrease in contextual anxiety (Guscott et al. 2000; Joordens et al. 1997; Zhao et al. 2018b). Fear potentiation can be expressed as “absolute value of the magnitude of the startle response to cued trials” (**C**, yellow bar), “the difference score” (startle response to cued trials − startle response to non-cued trials; **C**, striped bar), or as “percent fear potentiation” (100 × ((startle response to cued trials − startle response to non-cued trials)/startle response to non-cued trials))

One way to improve this translation is to use tests that are based on processes that directly relate to the human disorder under study and that can be used in both humans and animals (Bach 2022; Hendriksen and Groenink 2015). One such test is the fear-potentiated startle test (for description of the test procedure, see Fig. 1). The fear-potentiated startle test is based on classical fear conditioning. Fear learning is considered a central process in the development of anxiety-like disorders (Bouton et al. 2001; Duits et al. 2015; Lissek 2012; Mineka and Oehlberg 2008). The fear-potentiated startle test has been used as an experimental model for anxiety in both humans and animals. The methods to induce conditioned fear in this test are very similar across species (Fendt and Koch 2013; Lezak et al. 2017; Klumpers et al. 2010). Since both humans and animals show a potentiation of the startle response in anticipation of an electric shock, and the neural circuitry that causes this response is highly comparable between humans and animals (for reviews see Fendt and Koch 2013; Lezak et al. 2017), one could argue that the fear-potentiated startle test has a certain degree of construct validity (Luyten et al. 2011). However, the test does not model the mechanisms that underly pathological anxiety; the potentiated conditioned startle response measured in naïve animals is a healthy, adaptive response whereas in patients the exaggerated startle responses is a result of maladaptive processes (Willner 1986).

According to the DSM-V, one of the main symptoms of generalized anxiety disorders is an exaggerated startle response. In addition, individuals with panic disorder (Grillon et al. 1994) or posttraumatic stress disorder (Duits et al. 2015; Grillon et al. 1999) show an enhanced startle response relative to healthy controls. This exaggerated startle response in patients is thought to reflect an acute affective response to trauma-related cues (Grillon and Baas 2003). This enhanced startle response in patients resembles the enhanced startle response in animals, which is induced by the central state of fear elicited by the conditioned stimulus in the fear-potentiated startle test. As such, the fear-potentiated startle has face validity with regard to at least one of the symptoms of generalized anxiety disorder, panic disorder, and posttraumatic stress disorder (Willner 1986).

In animals, the fear-potentiated startle test is generally considered to have good predictive validity. Several research groups showed that clinically used anxiolytics such as benzodiazepines reduce fear potentiation, whereas other non-anxiolytic psychoactive drugs do not (Davis et al. 1988; Hijzen et al. 1995; Steiner et al. 2012). The predictive validity of the human fear-potentiated test is, however, not unequivocal since benzodiazepines do not consistently reduce fear potentiation in healthy human subjects (Baas et al. 2002; Grillon et al. 2006 but see Riba et al. 2001). Interestingly, all three human studies indicated that benzodiazepines do reduce conditioned contextual anxiety in this test. During fear conditioning, subjects not only acquire the cue-shock contingency but also learn to associate the foot shock with the experimental context. Re-exposure to the context may induce sustained, contextual anxiety (Grillon et al. 2007). This anxiety state also holds important resemblance to the pathology of human anxiety (Grillon and Ernst 2020). In humans, both cued-conditioned and sustained contextual anxiety states can well be discriminated and measured within the same fear potentiation startle test (Baas et al. 2004). In a standard fear-potentiated startle test in animals, contextual fear cannot readily be assessed, which could be seen as a limitation of the test (Groenink et al. 2008).

The fear-potentiated startle test has often been used to assess anxiolytic-like properties of drugs in animals. The effects of pharmacological interventions on the fear-potentiated startle, however, have not been systematically reviewed. With this systematic review, we aimed to determine which drug classes alter the expression of conditioned fear in the fear potentiated startle test in animals and to evaluate the predictive validity of the test. We identified, appraised, and synthesized the effects of all drugs that have been tested in animal studies using this test. We limited this review to single-dose studies using systematic drug administration because we were primarily interested in the fear-potentiated startle test as a drug screen for anxiolytic drug effects, and this focus would help to reduce heterogeneity. Since drug-induced changes in the non-cued baseline startle response may complicate the interpretation of drug effects on fear-potentiated startle (Groenink et al. 2008), we also determined the effect of all drug classes on the non-cued baseline startle response. Given that the fear-potentiated startle response and its sensitivity toward drugs may depend on species as well as strain, these factors were also included in the analysis (Risbrough et al. 2009; Risbrough and Geyer 2005; Steiner et al. 2011). We further included characteristics of both the training and test procedures since these may also affect test outcome (Davis and Astrachan 1978; de Jongh et al. 2003). Analysis of these methodological factors may help to optimize and refine testing procedures for future use.

## Materials and methods

The systematic review was performed following a pre-determined protocol (http://www.crd.york.ac.uk/PROSPERO/display_record.php?ID=CRD42018116762). Key elements of this protocol are described in the following paragraphs.

### Literature search and study selection

Studies reporting on drug effects in the fear-potentiated startle test in animals were identified by electronic searching of PubMed and Embase from inception up to September 9, 2021. There were no restrictions regarding the publication date. The search strategy aimed to identify any article that reported on the fear-potentiated startle test in animals. The search was not restricted by language or pharmacological intervention (for details see Table 1).

**Table 1:** Search strategy used to identify relevant articles

Database	Search string	Hits
*Pubmed*	(("reflex, startle"[MeSH Terms] OR “startle”[All Fields]) AND ( "fear"[MeSH Terms] OR "fear"[All Fields] OR "anxiety"[MeSH Terms] OR "anxiety"[All Fields] OR “anxious”[All Fields] OR “anxieties”[All Fields] OR "emotions"[MeSH Terms:noexp] OR "emotion"[All Fields] OR "conditioning"[All Fields] OR “conditioned”[All fields]) AND animal filter for Pubmed (Hooijmans et al. 2010)	*2042*
*Embase*	(‘startle reflex’/exp OR startle) AND (‘Fear’/exp OR fear OR anxiety OR anxious OR anxieties OR ‘emotion’/de OR emotion OR conditioned OR conditioning) AND animal filter for Embase Elsevier (de Vries et al. 2014)	*2479*

Peer-reviewed articles retrieved in the literature search were screened for eligibility using predefined inclusion and exclusion criteria based on the title and the abstract. Eligible for inclusion were studies performed in animals (regardless of strain, age, or sex) studying the effect of acute, systemic drug treatment on the expression of fear-potentiated startle, relative to control animals receiving vehicle treatment. We only included studies performed in naïve animals since pretreatments can affect the acquisition, retention, or spontaneous extinction of conditioned fear, which in turn could affect the efficacy of the drugs under investigation. In addition, to formulate recommendations on how to optimize and refine the experimental procedure and to reduce heterogeneity, we aimed to compare experiments that were sufficiently similar regarding the aversive and conditioned stimuli, as well as the stimuli used to elicit the startle response. We therefore only included studies that used a foot shock as unconditioned stimulus, a cue light as conditioned stimulus, and an acoustic stimulus to elicit the startle response. This combination of stimuli is commonly used if the fear-potentiated startle test is used for drug screening. Since the experimental set-up of the training and test sessions may well depend on the modalities of the stimuli that are used (Campeau & Davis 1995; Lonsdorf et al. 2017; Walker et al. 2005), focus on these specific stimuli would help to reduce heterogeneity in the data set.

Based on these inclusion criteria, the following set of exclusion criteria were used during both screening phases:

1. The study was not performed in animals.
2. The study did not describe the effect of drug treatment on the fear potentiated startle compared to control animals receiving vehicle treatment.
3. The animals underwent any prior treatment, stress manipulations, brain lesions, genetic modification, or other interventions aimed at altering the baseline level of the fear-potentiated startle response.
4. The drug was not systemically administered (e.g., intra-cerebrally).
5. The drug was administered chronically or repeatedly.
6. The study only reported on the acquisition or extinction of fear-potentiated startle or other measures of anxiety.
7. The study used alternative stimuli to induce startle potentiation, such as air puffs, noise, tone, or odor.
8. The study was not a full research report presenting original data (e.g., a review article).

The screening was performed independently by two investigators using EROS 2.0 (Early Review Organizing Software; Institute for Clinical Effectiveness and Health Policy, Buenos Aires, Argentina) and Rayyan (https://www.rayyan.ai/). Discrepancies were solved by discussion between these investigators (a third investigator was available to serve as arbiter in case consensus could not be reached, but did not occur).

### Data extraction

Included articles were randomly allocated to two investigators who independently extracted the predefined study characteristics from the articles (see Table 2). Outcome data were extracted by one investigator, and a second investigator then checked the extracted data. Discrepancies were solved by discussion between the investigators or where necessary with a third investigator.

**Table 2:** Overview of study characteristics and outcome data that were extracted

Study categories	Extracted study characteristics
*Animal characteristics and housing conditions*	Species, strain, sex, body weight, age, group- or single-housing, time of testing relative to dark–light cycle
*Experimental conditions*	
*General*	Brand of test chambers, calibration of the test box, background noise intensity, acclimation procedure
*Acquisition training*	Foot shock intensity, shock duration, cue-light duration, the timing of the shock relative to cue presentation, number of cue-shock pairings, mean interval between pairings, number of conditioning sessions per day
*Test session*	The time between training and test, characteristics of context, acclimatization, and habituation, number of cued and non-cued trials per session, trial order, startling noise intensity, startling noise duration, the interval between stimuli, cue-light duration, the timing of startling noise relative to cue light presentation, duration of data sampling, number of tests per week
*Pharmacological intervention*	Drug name, mode of action, dose (mg/kg), route of administration, injection-test interval
*Outcome measures*	Group mean and disperion for baseline startle (i.e., startle responses to non-cued trials), fear-potentiated startle (i.e., startle responses to cued trials), percent fear potentiation, absolute difference between the startle responses to cued and non-cued trials (i.e., difference score). Number of animals in the intervention and control groups, and study design (within or between-subjects design)
*Reporting of key study quality indicators*	Reporting of randomization at any level (yes/no), reporting of allocation based on baseline characteristics (yes/no), reporting of blinding at any level (yes/no), and reporting of a sample size calculation (yes/no)

### Data synthesis and meta-analysis

The study characteristics, quality assessment, and outcome data of any included study were reported in the systematic map (Supplementary File 2). We also described and summarized the effects of all drugs that have been tested (Fig. 3, Table 3). However, we only discussed effects of drug classes that had been tested at least three times and for which effect sizes could be calculated (Figs. 4, 5, 6, and 7).

We conducted a meta-analysis on fear potentiation and on the non-cued baseline startle response for each drug class or drug that was tested in at least five independent experiments and for which results had been reported in at least three different articles. For each experiment, we only included data for the most effective dose tested in the meta-analysis (the most effective dose was defined as the dose inducing the largest difference in outcome measure relative to the vehicle control condition) because inclusion of drug effects obtained at suboptimal doses would interfere with the interpretation of the effect size estimates. Experiments for which the mean startle magnitude, group size, or variance was missing could not be included in meta-analysis, but are included in a descriptive synthesis.

Data were analyzed using Comprehensive Meta-analysis version 3 (Biostat, NJ). We selected only one effect size per experiment, and although not all data were independent for all experiments, we treated them as such. If more than one outcome measure was reported for an experiment, we applied the following order for data inclusion in the meta-analysis: (1) difference score, (2) absolute values converted to difference score and corresponding standard error, and (3) percent fear-potentiated startle. Data are presented as standardized difference in means (SMD) with corresponding 95% confidence interval (95% CI). An overall SMD with a 95% CI was calculated for each of the drug classes by pooling all the individual effect sizes.

We used a random-effects model of DerSimonian and Laird to account for expected heterogeneity between experiments. Heterogeneity was assessed using *I*^2^ and tau^2^ statistics. We used the Holm–Bonferroni method to correct for multiple testing of the secondary outcome measure and of the subgroup variables separately for quality items and study characteristics.

We pre-specified drug class as the unit of analysis (based on mechanism of action, to be defined separately for clinically active anxiolytics and experimental drugs). For methodological characteristics and quality items, subgroup analyses were performed for each drug class or drug for which at least 10 experiments were available from at least 5 different articles. Pre-specified subgroup variables were species used, sex (males, females, mixed groups), time of testing (active or inactive phase of day–night cycle), cue-shock pairings (total number; 1–2/3–10/ > 10x), shock intensity (< 0.3 mA/0.3–0.8 mA/ > 0.8 mA), test context (same or different from training context), startle noise intensity (dB above background; < 20/20–35/ > 35 dB), and study design (within- or between-subjects design). Subgroups were omitted from the analysis if they contained less than five experiments, from fewer than three different articles.

We performed three sensitivity analyses for pooling different outcome measures of the fear potentiation. For this, we planned separate analyses for experiments reporting absolute fear-potentiated startle values, experiments reporting percent fear-potentiated startle, and experiments that reported fear potentiation as a difference score. Further sensitivity analyses were planned for the categories created for shock intensity, cue-shock pairings, and startle noise intensity.

Publication bias was investigated for each outcome measure separately, using visual inspection of funnel plots, Egger’s regression, and trim-and-fill analysis.

## Results

### Article selection and search results

**Fig. 2:**
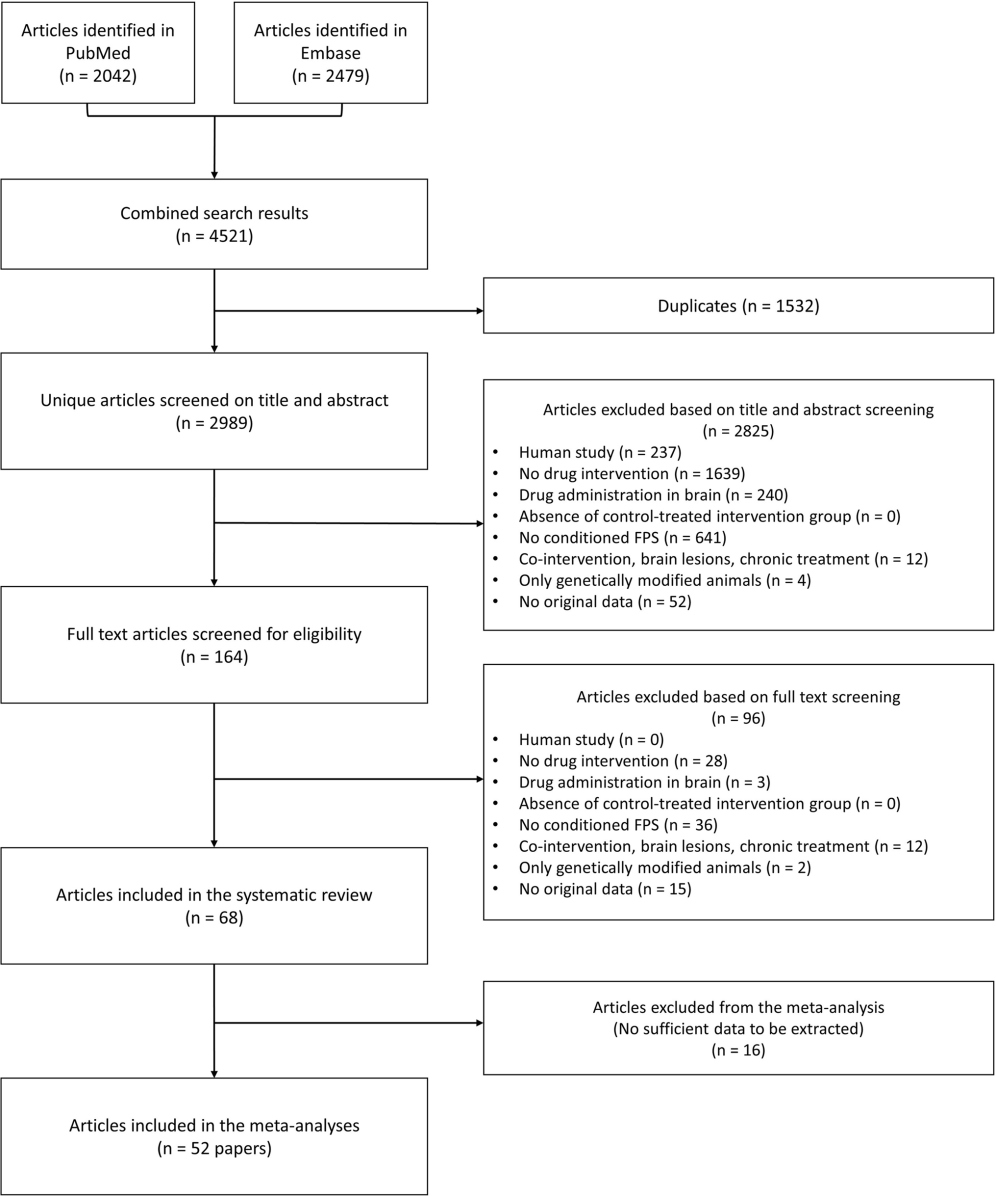
Flowchart of the article selection process

As shown in the flowchart (Fig. 2), the electronic search retrieved 2989 unique articles. After screening for eligibility, 68 were included in this systematic review, all of which were published in English. An analysis of the year of publication of the included articles showed that the first paper that studied acute, systemic drug effects in the fear-potentiated startle test was published in 1965. The number of publications peaked between 2000 and 2010 and has gradually declined since (Supplementary File 1).

Here, we report the results of 201 experiments (i.e., experimental comparisons between a control and a treated group) from these 68 articles.

### Study characteristics

Below, we summarize the characteristics that were predefined for subgroup analysis. A complete overview of animal and housing characteristics per article is provided in Supplementary File 3. A synthesis of the methodological characteristics used is shown in Supplementary File 4. All details for individual articles can be found in the searchable systematic map together with the outcome of all individual experiments (Supplementary File 2).

#### Animal characteristics and housing conditions

In 64 of the 68 articles, drugs were tested in rats. Sprague–Dawley (31 articles) and Wistar rats (20 articles) were the most frequently used rat strains (Supplementary File 3). Only two articles compared drug effects in male and female rats (Toufexis et al. 2016; Zhao et al. 2018a). The other four articles used mice of four different strains (129SvEv, C57BL/6 J, CD-1, and DBA/1 J). In one of these four articles, female mice were included in the experimental design. Since almost all drugs were tested in rats, we will only specifically refer to species in those cases where mice were used (11 experiments).

In nine articles, animals were tested in the active phase of the dark–light cycle. In 33 articles, animals were tested during the inactive, dark phase. In the other 26 articles, it was not reported when the animals were tested.

#### Methodological characteristics of the acquisition training

For the acquisition training, a variety of protocols was used. Protocols mostly varied in the number of cue-shock pairings presented, number of training sessions, and number of training days. A commonly used set-up consisted of a foot shock with an intensity of around 0.6 mA for a duration of 500 ms, which was delivered during the last 500 ms of a 3700-ms cue-light presentation (Supplementary File 4, Supplementary File 5). Remarkably, 11 of the 68 articles did not report any information on the training procedure.

To compare the results obtained with different training protocols, we calculated the total number of cue-shock pairings based on the reported number of training sessions and the number of cue-shock pairings per session (Supplementary File 5). This could be done for 59 of the 68 articles included in this systematic review. In most articles (23), a total number of 20 cue-shock pairings was used. In these articles, pairings were most times divided over two sessions of ten cue-shock pairings each (21 of 23 articles). In the 13 articles that presented 10 pairings in total, all pairings were presented within one session. The lowest number of pairings presented was five (within one session, two articles), and the highest number of pairings was 90, that is, 45 pairings in two sessions. The four articles that applied the latter protocol are among the earlier publications (Davis 1979; Hijzen and Slangen 1989).

The intensity of the foot shocks that were presented during fear conditioning varied between 0.14 and 1.25 mA (reported in 60 articles; Supplementary File 5). In most articles (45), shock intensities between 0.4 and 0.6 mA were used. Six articles used foot-shock intensities of 1.0 mA or above (1.25 mA; four and two articles, respectively). The lowest shock intensity, 0.14 mA, was only used for acquisition training with mice (two articles).

#### Methodological characteristics of the test sessions

In 35 articles, one single startling noise intensity was used, whereas in 26 articles more than one noise intensity was used. In seven articles, the intensity of the startling noise was not reported. Using the articles that reported both background noise and startle noise intensities (49 articles), we calculated that the startling noise intensity varied between 21 and 65 dB above the background noise. In absolute values, the lowest intensity used was 85 dB, and the highest intensity was 122 dB (Supplementary File 5).

#### Pharmacological interventions

Together, the included articles reported on the effects of 103 different drugs. We categorized these drugs into 56 different drug classes. Drug classes were based on neurotransmitter system and mechanism of action, except for benzodiazepines and barbiturates which were both categorized separately from other GABA_A_ receptor–positive allosteric modulators.

Most articles reported on drugs that target the GABA-ergic system (35 articles), the serotonergic system (16 articles), or the glutamatergic system (18 articles). Drugs acting on the noradrenergic system (4 articles), the dopaminergic system (5 articles), or the opioid system (4 articles) were less frequently studied. Seventeen articles studied drug effects on neuropeptide systems other than the opioid system (ten different systems). The remaining experiments were categorized under “miscellaneous” (4 articles) comprising the cholinergic system (2 articles), the endocannabinoid system (1 article), the glucocorticoid system (1 article), and “other” (1 article, testing carbamazepine, a voltage-dependent sodium channel blocker).

In Table 3, we present an overview of all drugs that were tested in the fear-potentiated startle test. The drug effects that are listed in this table are based on significance as reported by the authors of the respective articles. In total, the results of 201 experiments were reported. In 60 experiments, clinically used anxiolytics were tested. Experimental compounds were tested in 141 experiments, including 17 experiments that tested putative anxiogenic drugs. In general, the reported effects on both fear potentiation and baseline startle were consistent among experiments that tested the same drug classes.

#### Outcome measures

The primary outcome parameter in the fear-potentiated startle test is potentiation of the acoustic startle response. As shown in Fig. 1, this outcome can be reported in various units of measurement, namely, “the magnitude of the startle response to cued trials,” “percent fear potentiation” (100 × ((startle response to cued trials − startle response to non-cued trials)/startle response to non-cued trials)), or “the difference score” (startle response to cued trials − startle response to non-cued trials). Reported drug effects on these outcomes are summarized in the respective columns in Table 3. In 45 of the 68 articles (145 of the 201 experiments), drug effects were reported as absolute startle response values (“the magnitude of the startle response to cued trials”). In 17 articles (35 experiments), results were reported as percent fear potentiation, and in 17 articles (76 experiments), drug effects were reported as difference score. In 22 articles (85 experiments), data were reported in more than one unit of measurement. In 16 articles (38 experiments), outcome data were missing or incomplete (mean, dispersion, or group sizes were not reported); therefore, 19% of eligible data could not be included in meta-analysis.

We analyzed drug effects on non-cued baseline startle response as a secondary outcome parameter. The reported effects on this parameter are shown as *non-cued* in the tables. Drug effects on the non-cued baseline startle response were reported for 145 of the 201 experiments (72% of the experiments).

#### Qualitative data synthesis

**Fig. 3:**
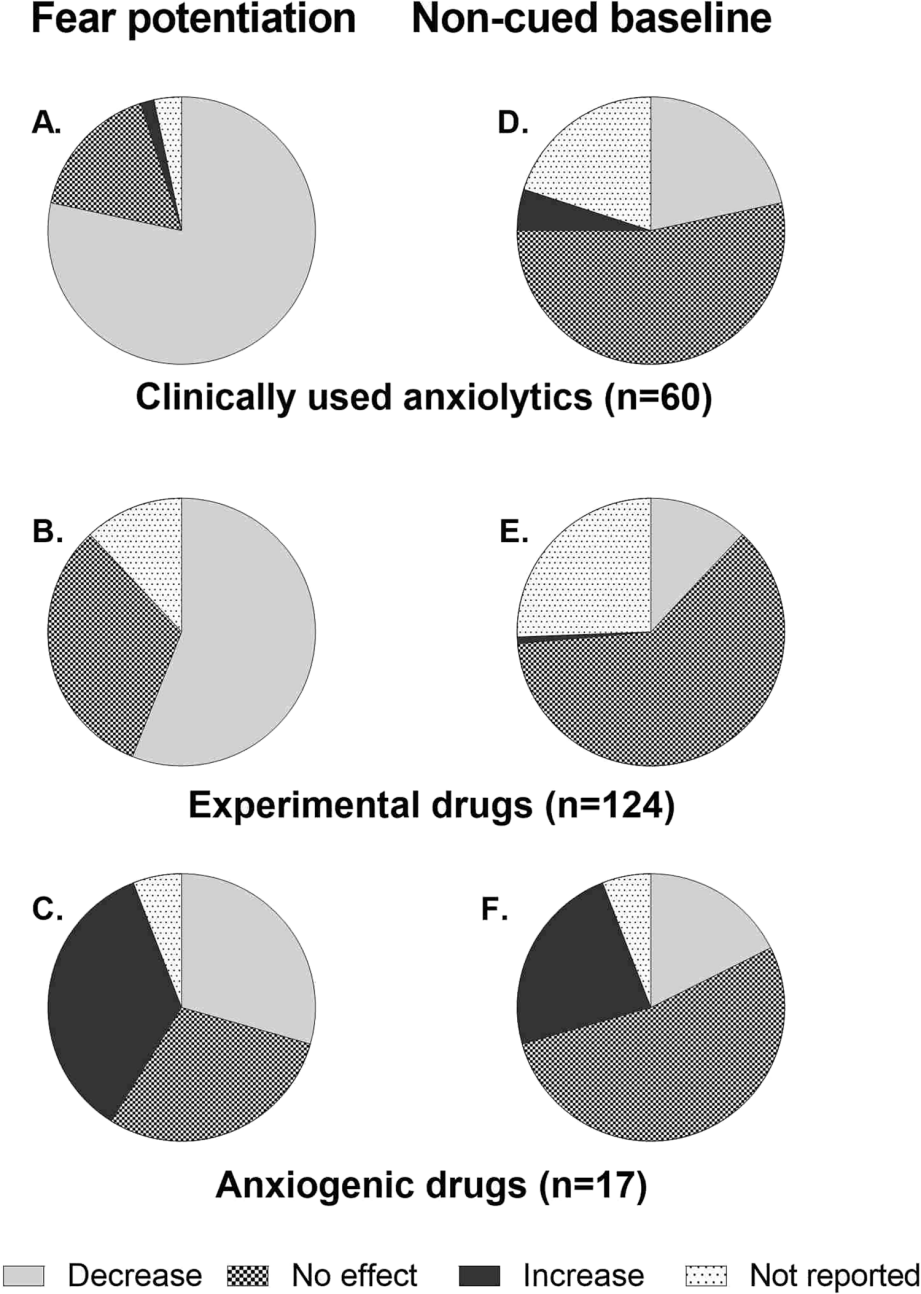
Reported effects on fear potentiation (left panel) and the non-cued baseline startle response (right panel) for clinically used anxiolytics (2A, 2D), experimental drugs (2B, E), and anxiogenic drugs (2C, F). Data are shown as a percentage of the total number of experiments (*n*) performed for each drug category. Non-cued baseline = the non-cued baseline startle response

In most experiments (78%), clinically used anxiolytics reduced fear potentiation, whereas in 10% of the experiments, these drugs had no effect on fear potentiation (Fig. 3a). In 53% of the experiments, clinically used anxiolytics had no effect on the non-cued baseline startle response (Fig. 3d). In 22% of the experiments, clinically used anxiolytics reduced the non-cued baseline startle response, and in three experiments (5%), the drugs under study (buspirone and sertraline) enhanced the non-cued baseline startle response (Fig. 3d, Table 3).

In 56% of the experiments that tested experimental drugs, these drugs reduced fear potentiation (Fig. 3b). This percentage is in line with the percentage of experiments for which the researcher expected the experimental drug to have anxiolytic-like effects (57%; Supplementary File 2). In 32% of the experiments, the drugs had no effect on fear potentiation (Fig. 3b), whereas for 16% of the experiments, researchers expected the experimental drug to be devoid of effects on fear potentiation. In 2% of the experiments, anxiogenic-like drug effects were expected, but for none of the experimental drugs an increase in fear potentiation was reported. For 33% of the experiments, the expected drug effects on fear potentiation were not reported in the articles. Experimental drugs reduced the non-cued baseline startle in 12% of the experiments, whereas the absence of effect on the non-cued baseline startle was reported for 62% of the experiments (Fig. 3e). For none of the 124 experimental drugs expected effects on non-cued baseline startle were reported.

As shown in Fig. 3c, 35% of the experiments that tested anxiogenic drugs reported an increase in fear potentiation. In a comparable number of experiments, anxiogenic drugs reduced fear potentiation (29%) or had no effect (29% of the experiments), whereas for 5% of the experiments, drug effects on fear potentiation were not reported. In half of the experiments (53%), anxiogenic drugs did not alter the non-cued baseline startle response. For the other experiments, either an increase (24%) or a decrease (18%) in the non-cued baseline startle response was reported, or data were not reported (5%) (Fig. 3f).

### Quality of reporting and study design

We assessed reporting of four predefined key indicators of study quality (scores for individual articles and a summary graph are shown in Supplementary File 6).

Only one of the 68 selected articles reported that a sample size calculation had been performed to assure adequate power to detect statistical significance. Three articles reported on blinding of treatment conditions. Eight articles (12%) reported that animals were randomly allocated to treatment groups, however without description of the method of randomization used. In 37 articles (54%), animals were allocated to experimental groups based on their baseline (potentiated) startle amplitude characteristics, to control for individual differences in the startle response. In 33 of these 37 articles, the mean startle response of animals as measured before (31) or after (2) the fear-conditioning training was used to do so. In the other four articles, percent fear-potentiated startle was used to compose comparable experimental groups. In eight articles, the read-out that was used to compose equal groups was not specified. Two of the 37 articles detailed how the actual allocation was performed when baseline characteristics were used to allocate the animals. These articles reported that treatment groups were matched for equivalent numbers of high and low amplitude startle responders as measured after training (Anthony and Nevins 1993; Nevins and Anthony 1994). Fourteen articles (9.5%) did not report measures that could reduce selection bias.

Regarding study design, a between-subjects design was used in 51 articles and a within-subjects design in 10 articles. In the remaining seven articles, the study design that was used was not reported (Supplementary File 6). The group size of the experimental groups ranged from 5 to 55 animals per group (Supplementary File 4). In 10 articles, a balanced within-subject design (Latin-square) was used to control for baseline differences. In one article, the mean startle amplitude measured before fear conditioning was included as a covariate in the statistical analysis to control for possible confounding effects of baseline differences (Hijzen et al. 1995).

### Pharmacology of the fear-potentiated startle test

#### GABA-ergic system

Ever since the discovery of benzodiazepines as clinically effective anxiolytics, the GABA-ergic system has been implicated in the modulation of anxiety. By now, the GABA-ergic system has been studied extensively in anxiety research using a wide range of experimental drugs (Castellano et al. 2020). On the one hand, attempts have been made to synthesize drugs that would interact more selectively (e.g., TPA023) or with lower efficacy (e.g., bretazenil) with the GABA_A_ receptor complex to overcome the adverse effects of prototypical benzodiazepines (Rudolph and Knoflach 2011). On the other hand, GABA_A_ receptor inverse agonists, including FG-7142, pentylene tetrazole, and DMCM, proved useful to study anxiogenic-like behavior (Pellow and File 1984). In addition, benzodiazepines have often been used as positive control condition in experimental animal studies.

##### Study characteristics for the GABA-ergic system

The included articles reported on 59 experiments in which 20 different GABA-ergic drugs were tested. These drugs were categorized into 10 different drug classes (Table 3). Regarding clinically used anxiolytics, two drug classes were studied, the benzodiazepines and barbiturates. Benzodiazepines were tested in 41 experiments, whereas the barbiturate amobarbital was tested once. Of the six registered benzodiazepines, diazepam and chlordiazepoxide were tested most frequently, in 25 and 10 experiments, respectively. The other benzodiazepines were tested once or twice (Table 3).

**Table 3:** Pharmacological interventions in the fear-potentiated startle test

Article	Drug	Species, strain	Dose (mg/kg)	Route, ITI (min)	Group size (*)	Reported effective dose (mg/kg); Direction of effect; ANOVA effect			
						Non-cued startle	Cued startle	Difference score	% FPS
**GABA-ergic system**									
*Benzodiazepines*									
(Hijzen et al. 1995)	Alprazolam	Rat, Wi	1, 2, 3	IP, 30	12	=	NR; ↓; cue × dose	NR	NR
(Jenck et al. 2000)	Alprazolam	Rat, Wi	0.32, 1, 3.2	IP, 30	16	1, 3.2; ↓	1, 3.2; ↓	NR	NR
(Steiner et al. 2011)	Alprazolam	Rat, F344	0.3, 1. 3	PO, NR	16	1, 3; ↓	0.3, 1, 3; ↓	0.3, 1, 3; ↓	1, 3; ↓
(Bill et al. 1992)	Chlordiazepoxide	Rat, LH	5	SC, 30	10	5; ↓	5; ↓	NR	NR
(Guscott et al. 2000)	Chlordiazepoxide	Rat, SDR	0.3. 1, 3, 10	IP, 20	10	1, 3, 10; ↓	1, 3, 10; ↓	3, 10; ↓	NR
(Guscott et al. 2000)	Chlordiazepoxide	Rat, SDR	0.3, 1, 3, 10	IP, 30	12	10; ↓	10; ↓	10; ↓	NR
(Hijzen et al. 1995)	Chlordiazepoxide	Rat, Wi	2.5, 5, 10	IP, 20	12	=	NR; ↓; cue × dose	NR	NR
(Joordens et al. 1996)	Chlordiazepoxide	Rat, Wi	2.5, 5, 10	IP, 20	8	=	NR; ↓; cue × dose	NR	NR
(Joordens et al. 1996)	Chlordiazepoxide	Rat, Wi	2.5, 5, 10	IP, 20	12	=	NR; ↓; cue × dose	NR	NR
(Joordens et al. 1997)	Chlordiazepoxide	Rat, Wi	3.2, 10, 32	SC, 20	8	=	NR; ↓; cue × dose	NR	NR
(Risbrough et al. 2003)	Chlordiazepoxide	Mouse, DBA/1 J	10	IP, 30	6	=	NR; data shown	NR	10; ↓
(Vale & Green 1996)	Chlordiazepoxide	Rat, HL	2.5, 5	IP, 30	10	=	2.5, 5; ↓	NR	NR
(Varty et al. 2008)	Chlordiazepoxide	Rat, Wi	1, 6, 10	IP, 30	12–14	=	NR	NR	10; ↓
(Zhao et al. 2018a)	Chlordiazepoxide	Rat, Wi	2.5, 5, 10	IP, 20	24	NR; ↓	NR; ↓; cue × dose	10; ↓	NR
(Zhao et al. 2018a)	Chlordiazepoxide	Rat, Wi	2.5, 5, 10	IP, 20	54	2.5, 5, 10 ↓	2.5, 5, 10 ↓	NR	10; ↓
Anthony & Nevins 1993)	Diazepam	Rat, SDR	1	IP, 30	8	=	NR; data shown	1; ↓	NR
(Brodkin et al. 2002)	Diazepam	Rat, Wi	0.3, 1, 3	SC, 30	8	=	3; ↓	NR	NR
(Busse et al. 2004)	Diazepam	Rat, Wi	0.3, 1, 3	SC, 30	8	=	3; ↓	NR	NR
(Collado et al. 2002)	Diazepam	NR	0.6	IP, NR	8	NR	NR	NR	NR
(Collado et al. 2004)	Diazepam	Rat, SDR	0.6	IP, NR	NR	NR	NR	0.6; ↓	NR
(Davis 1979)	Diazepam	Rat, SDR	0.31, 0.62, 1.25, 2.5	IP, 10	12	=	NR; data shown	NR	0.31, 0.62, 1.25, 2.5; ↓
(Gacsályi et al. 2017)	Diazepam	Rat, SDR	3	IP, 30	8–14	NR	NR	3, ↓	NR
(Helton et al. 1998)	Diazepam	Rat, LE	0.03, 0.1, 0.3. 1	IP, 30	8	NR	NR	0.1, 0.3, 1; ↓	NR
(Johnson et al. 2003)	Diazepam	NR	0.6	IP, NR	NR	NR	NR	0.6; ↓	NR
(Johnson et al. 2005)	Diazepam	Rat, SDR	0.6	IP, NR	NR	NR	NR	0.6; ↓	NR
(Johnson et al. 2005)	Diazepam	Rat, SDR	0.6	IP, NR	NR	NR	NR	0.6; ↓	NR
(Joordens et al. 1997)	Diazepam	Rat, Wi	1, 3.2, 10	SC, 30	8*2 external control	=	NR; ↓; cue × dose	NR	NR
(Martin et al. 2002)	Diazepam	Rat, RORO	0.3, 1, 3	IP. 30	12	1; ↓	0.3, 1, 3; ↓	0.3, 1, 3; ↓	NR
(Nevins & Anthony 1994)	Diazepam	Rat, SDR	0.32, 1, 1.78, 3.2	IP. 30	8	=	NR; data shown	1, 1.78, 3.2; ↓	NR
(Nevins & Anthony 1994)	Diazepam	Rat, SDR	0.31, 1, 1.78. 3.2	IP, 30	8	=	NR; data shown	1, 1.78, 3.2; ↓	NR
(Pietraszek et al. 2005)	Diazepam	Rat, SDR	0.5, 1, 2	IP, 30	14	NR	NR; data shown	2; ↓	NR
(Risbrough et al. 2003)	Diazepam	Mouse, DBA/1 J	1, 3, 6	IP, 30	9 to 15	=	6; ↓	NR	3, 6; ↓
(Rorick-Kehn et al. 2007)	Diazepam	Rat, SDR	0.6	PO, 30	8	NR	NR	0.6; ↓	NR
(Schulz et al. 2001)	Diazepam	Rat, SDR	1.25	IP, 30	10	NR	NR	NR	1.25; ↓
(Steiner et al. 2012)	Diazepam	Rat, F344	1, 3, 10	PO, 30	12	10; ↓	3, 10; ↓	NR	NR
(Tizzano et al. 2002)	Diazepam	Rat, SDR	0.3, 0.6, 1	IP, 30	8	=	NR; data shown	0.6, 1; ↓	NR
(Tizzano et al. 2002)	Diazepam	Rat, SDR	0.6	IP, 30	8	=	NR; data shown	0.6; ↓	NR
(Tizzano et al. 2002)	Diazepam	Rat, SDR	0.6	IP, 30	8	=	NR; data shown	0.6; ↓	NR
(Zhang et al. 2016)	Diazepam	Rat, SDR	2	SC, 10	10	NR	NR	NR	NR
(Zhao et al. 2018b)	Diazepam	Rat, Wi	0.3, 1, 3	IP, 30	24	NR; ↓, main effect	No cue × dose	NR	NR
(Davis 1979)	Flurazepam	Rat, SDR	2.5, 10, 20	IP, 10	8	=	NR; ↓	NR	NR; ↓
(Hijzen & Slangen 1989)	Midazolam	Rat, Wi	0, 0.5, 1, 2	IP, 10	12	NR; ↓, main effect	No cue × dose	NR	NR
(Joordens et al. 1996) Utrecht	Oxazepam	Rat, Wi	1, 3, 10	PO, 60	12	NR; ↓, main effect	No cue × dose	NR	NR
(Joordens et al. 1996) Oss	Oxazepam	Rat, Wi	1, 3, 10	PO, 60	12	NR; ↓	NR; ↓; cue × dose	NR	NR
*Barbiturates*									
(Chi 1965)	Amobarbital	Rat, SDR	10, 20, 40	IP, 10	10	=	10, 20, 40; ↓	NR	NR
*GABA*_*A*_* receptor agonists*									
(Hijzen et al. 1995)	Alcohol	Rat, Wi	500, 1000, 2000	IP, 30	12	=	=	NR	NR
*GABA*_*A*_* receptor partial agonists*									
(Guscott et al. 2000)	Bretazenil	Rat, SDR	0.1, 0.3, 1, 3	PO, 30	10–12	1; ↓	1, 3; ↓	1, 3; ↓	NR
(Guscott et al. 2000)	Bretazenil	Rat, SDR	0.1, 0.3, 1, 3	PO, 30	10–12	=	1; ↓	1; ↓	NR
(Guscott et al. 2000)	FG 8205	Rat, SDR	0.3, 1, 3, 10	IP, 30	11	1, 3, 10; ↓	1, 3, 10; ↓	3, 10; ↓	NR
(Guscott et al. 2000)	FG 8205	Rat, SDR	0.3, 1, 3, 10	IP, 30	10–12	=	3, 10; ↓	3, 10; ↓	NR
*GABA*_*A*_* receptor α1 subunit agonist*									
(Zhao et al. 2018b)	Zolpidem	Rat, Wi	1, 3, 10	IP, 15	24	3, 10; ↓	10; ↓	3; ↓	NR
GABA_A_ *receptor α2,3 subunit agonist*									
(Atack et al. 2006)	TPA023	Rat, SDR	0.3, 1, 3	PO, 30	15	=	1, 3; ↓	1, 3; ↓	NR
(Atack et al. 2011)	TPA023B	Rat, SDR	0.1, 0.3, 1	PO, 45	10	=	1; ↓	0.3, 1; ↓	NR
*GABA*_*A*_* receptor α5 subunit antagonist*									
(Gacsályi et al. 2017)	S44819	Rat, SDR	1, 3, 10	IP, 30	8–14	=	NR	1, 3; ↓	NR
*GABA*_*A*_* inverse receptor agonists*									
(Risbrough & Geyer 2005)	FG-7142	Mouse, DBA/1 J	1, 3, 10, 20	IP, 5	8 to 12	=	20; ↓	NR	10, 20; ↓
(Risbrough & Geyer 2005)	FG-7142	Mouse, 129/SvEv	1, 3, 10, 20	IP, 5	8 to 12	=	=	NR	3; ↓
(Hijzen & Slangen 1989)	Lindane	Rat, Wi	7.5, 15, 30	PO, 180	NR	NR; ↑; main effect	No cue × dose	NR	NR
(Bijlsma et al. 2010)	Pentylene tetrazole	Rat, Wi	3, 10, 30	IP, 10	9	NR; ↓; main effect	No cue × dose	NR	=
(Hijzen & Slangen 1989)	DMCM	Rat, Wi	0, 0.1, 0.2, 0.4	IP, 6	NR	NR; ↑; main effect	No cue × dose	NR	NR
*GABA*_*A*_* receptor antagonist*									
(Davis et al. 1988)	Flumazenil aka Ro 15–1788	Rat, SDR	2	NR, NR	5	NR	NR	NR	NR
(Tizzano et al. 2002)	Flumazenil	Rat, SDR	2	IP, 60	8	NR	NR	=	NR
*GABA*_*B*_* receptor positive allosteric modulator*									
(Li et al. 2015)	BHF177	Rat, Wi	20, 40	PO, 60	15–16	=	=	NR	=
Serotonergic system									
*5-HT*_*1A*_* receptor partial agonists (azapirones)*									
(Brodkin et al. 2002)	Buspirone	Rat, Wi	0.3, 1, 3	IP, 30	8	=	=	NR	NR
(Davis et al. 1988)	Buspirone	Rat, SDR	5	SC, 0	5	NR	NR; ↓; cue x dose	NR	NR
(Li et al. 2015)	Buspirone	Rat, Wi	1, 3	SC, 10	12	=	3; ↓	NR	3; ↓
(Mansbach & Geyer 1988)	Buspirone	Rat, SDR	1.25, 2.5, 5	SC, 10	10	1.25, 5 ↑	=	2.5, 5.0; ↓	2.5, 5.0; ↓
(Nevins & Anthony 1994)	Buspirone	Rat, SDR	0.56, 1, 3.2, 5.6	IP, 30	8	=	NR; data shown	1, 3.2, 5.6; ↓	NR
(Nevins & Anthony 1994)	Buspirone	Rat, SDR	0.56, 1, 3.2, 5.6	IP, 30	8	=	NR; data shown	1, 3.2, 5.6; ↓	NR
(Risbrough et al. 2003)	Buspirone	Mouse, DBA/1 J	2.5, 5, 10	SC, 30	9 to 11	=	=	NR	5, 10; ↓
(Steiner et al. 2012)	Buspirone	Rat, F344	10, 30, 100	PO, 30	12	10, 30, 100; ↑	100; ↓	NR	NR
(Mansbach & Geyer 1988)	Gepirone	Rat, SDR	3, 10	IP, 10	7 to 8	=	=	=	10; ↓
(Davis et al. 1988)	Ipsapirone	Rat, SDR	10, 20, 40	IP, 0	5	40; ↓	40; ↓	40; ↓	NR
(Mansbach & Geyer 1988)	Ipsapirone	Rat, SDR	1, 3, 5.6, 10	SC, 10	10	=	=	5.6; ↓	3.0, 5.6; ↓
*5-HT*_*1A*_* biased receptor agonists*									
(Zhao et al. 2019)	F13714	Rat, Wi	0.01, 0.04, 0.16	IP, 60	12	0.04, 0.16; ↓	0.16; ↓	NR	NR
(Zhao et al. 2019)	F15599	Rat, Wi	0.01, 0.04, 0.16	IP, 60	12	=	=	NR	NR
*5-HT*_*1A*_* receptor agonists*									
(Davis et al. 1988)	8-OH-DPAT	Rat, SDR	2.5, 5, 10	IP, 0	5 (10)	NR	NR	no dose effect	NR
(Joordens et al. 1998)	8-OH-DPAT	Rat, Wi	0.03, 0.1, 0.3	SC, 10	12	=	0.3; ↓	NR	NR
(Joordens et al. 1998)	8-OH-DPAT	Rat, Wi	0.3	SC, 10	35 (36)	NR	NR	0.3; ↓	NR
(Mansbach & Geyer 1988)	8-OH-DPAT	Rat, SDR	0.125, 0.5	SC, 10	10	=	=	0.125, 0.25; ↓	0.125, 0.25; ↓
(Zhao et al. 2019)	R( +)-8-OH-DPAT	Rat, Wi	0.03, 0.1, 0.3	SC, 10	12	0.03, 0.1, 0.3; ↓	0.03, 0.1, 0.3; ↓	NR	NR
(Joordens et al. 1998)	Flesinoxan	Rat, Wi	10	PO, 60	35 (36)	NR	NR	10; ↓	NR
(Joordens et al. 1996) Utrecht	Flesinoxan	Rat, Wi	1, 3, 10	PO, 60	12	=	NR; ↓; cue x dose	NR	NR
(Joordens et al. 1996) Oss	Flesinoxan	Rat, Wi	1, 3, 10	PO, 60	12	dose NR; ↓	NR; ↓; cue x dose	NR	NR
*5-HT*_*1A*_* receptor antagonists*									
(Joordens et al. 1998)	DU125,530	Rat, Wi	1, 3, 10	SC, 30	12	=	1, 3, 10; ↓	NR	NR
(Joordens et al. 1998)	Pindolol	Rat, Wi	3, 10, 30	SC, 30	11	=	10, 30; ↓	NR	NR
(Joordens et al. 1998)	WAY100,635	Rat, Wi	0.1, 0.3, 1	SC, 30	12	=	1; ↓	NR	NR
(Risbrough & Geyer 2005)	WAY100,635	Mouse, DBA/1 J	0.3	IP, 10	8 to 12	=	=	NR	NR
(Zhao et al. 2019)	WAY100,635	Rat, Wi	0.1, 0.3, 1	SC, 30	12	0.1, 0.3, 1; ↓; main effect	no cue x dose	NR	NR
*5-HT*_*2c*_* receptor agonists*									
(Bijlsma et al. 2010)	m-CPP	Rat, Wi	0.5, 1, 2	IP, 25	8	NR; ↓, main effect	no cue x dose	NR	2; ↑
(Mansbach & Geyer 1988)	m-CPP	Rat, SDR	0.25, 1	SC, 10	10	1; ↓	1; ↓	=	=
(Risbrough & Geyer 2005)	m-CPP	Mouse, DBA/1 J	0.3, 1, 3	SC, 15	8 to 12	3; ↑	3; ↑	NR	=
*5-HT*_*2a, 2c*_* receptor antagonists*									
(Davis et al. 1988)	Cinanserin	Rat, SDR	10	IP, NR	5	NR	NR	NR	NR
(Mansbach & Geyer 1988)	Methysergide	Rat, SDR	0.3, 1, 3, 10	SC, 10	7 to 8	1; ↑	=	=	=
(Davis et al. 1988)	Cyproheptadine	Rat, SDR	5	NR, NR	5	NR	NR	NR	NR
(Martin et al. 2002)	SB-242084	Rat, RORO	0.1, 0.3, 1	IP, 30	12	=	=	=	NR
*5-HT*_*3*_* receptor antagonists*									
(Nevins & Anthony 1994)	(R)-Zacopride	Rat, SDR	0.001, 0.01, 0.1, 1	IP, 45	8	=	NR; data shown	1; ↓	NR
(Nevins & Anthony 1994)	(R)-Zacopride	Rat, SDR	0.001, 0.01, 0.1, 1	IP, 45	8	=	NR; data shown	0.01, 0.1, 1; ↓	NR
(Nevins & Anthony 1994)	Granisetron	Rat, SDR	0.001, 0.01, 0.1, 1	IP, 45	8	=	NR; data shown	=	NR
(Nevins & Anthony 1994)	Granisetron	Rat, SDR	0.001,0,01, 0,1, 1	IP, 45	8	=	NR; data shown	0.01, 0.1, 1; ↓	NR
(Nevins & Anthony 1994)	Ondansetron	Rat, SDR	0.001, 0.01, 0.1, 1	IP, 45	8	=	NR; data shown	=	NR
(Nevins & Anthony 1994)	Ondansetron	Rat, SDR	0.001, 0.01, 0.1, 1	IP, 45	8	=	NR; data shown	0.001, 0.01, 0.1, 1; ↓	NR
(Nevins & Anthony 1994)	Ondansetron	Rat, SDR	0.1	IP, 45	8	=	NR; data shown	0.1; ↓	NR
(Nevins & Anthony 1994)	Ondansetron	Rat, SDR	0.1	IP, 45	8	=	NR; data shown	=	NR
(Nevins & Anthony 1994)	Ondansetron	Rat, SDR	0.1	IP, 45	8	=	NR; data shown	0.1; ↓	NR
(Nevins & Anthony 1994)	Ondansetron	Rat, SDR	0.1	IP, 45	8	=	NR; data shown	0.1; ↓	NR
Bill et al. 1992	WAY100289	Rat, LH	0.003, 0.03, 0.3	SC, 30	10	NR	NR	NR	NR
*5-HT re-uptake inhibitors (SSRIs)*									
(Steiner et al. 2012)	Fluoxetine	Rat, F344	3, 10, 30	PO, 45	12	=	=	NR	NR
(Joordens et al. 1996)	Fluvoxamine	Rat, Wi	5, 10, 20	PO, 60	12	=	=	NR	NR
(Joordens et al. 1996)	Fluvoxamine	Rat, Wi	5, 10, 20	PO, 60	12	=	=	NR	NR
(Steiner et al. 2012)	Sertraline	Rat, F344	10, 30, 100	PO, 60	12	30, 100; ↑	30, 100; ↑	NR	NR
*TCAs*									
(Hijzen et al. 1995)	Amitriptyline	Rat, Wi	2.5, 5, 10	IP, 60	12	=	=	NR	NR
Cassella 1985	Imipramine	Rat, SDR	5, 10	IP, 0	10	=	=	=	NR
(Nevins & Anthony 1994)	Imipramine	Rat, SDR	5, 10	IP, NR	8	=	NR; data shown	=	NR
*5-HT releaser*									
(Davis et al. 1988)	p-Chloro-amphetamine	Rat, SDR	5	IP, 15	5	NR	NR	NR	NR
Glutamatergic system									
*mGluR1antagonists*									
(Pietraszek et al. 2005)	EMQMCM	Rat, SDR	1.25, 2.5, 5	IP, 30	16	=	NR; data shown	5; ↓	NR
*mGLuR5 antagonists*									
(Carcache et al. 2011)	15i	NR	0.3, 1, 3	PO, 60	10	NR	NR;data shown	1, 3; ↓	NR
(Brodkin et al. 2002)	MPEP	Rat, Wi	3, 10, 30	IP, 60	8	=	10, 30; ↓	NR	NR
(Schulz et al. 2001)	MPEP	Rat, SDR	0.3, 3, 10	PO, 60	NR	=	30; ↓	30; ↓	NR
(Cosford et al. 2003)	MPEP	NR	NR	IP, NR	8	NR	NR	NR	NR
Busse et al. 2004	MTEP	Rat, Wi	0.3, 1, 3	IP, 60	8	=	1, 3; ↓	NR	NR
(Pietraszek et al. 2005)	MTEP	Rat, SDR	0.6, 1.25, 2.5,	IP, 30	14	=	NR, data shown	2.5, 5; ↓	NR
(Cosford et al. 2003)	MTEP	NR	NR	IP, NR	8	NR	NR	NR	NR
(Roppe et al. 2004a)	5-[(2-Methyl-1,3-thiazol-4-yl)ethynyl]-2,30-bipyridine (MTEP derivate	NR	NR	PO, 60	NR	NR	NR	NR	NR
(Roppe et al. 2004b)	3-(5-pyridin-2-yl-2H-tetrazol-2-yl)benzonitrile (MTEP derivate)	Rat, Wi	NR	PO, 60	8	NR	NR	NR	NR
*mGluR2,3 agonists*									
(Collado et al. 2004)	(2S,1'S,2'R,3'R)-2-(2¢-carboxy-3¢-hydroxymethylcyclopropyl) glycine (( +)-3)	Rat, SDR	0.003, 0.04, 0.3, 3 µg/kg	, NR	NR	NR	NR	0.3, 3 µg/kg; ↓	NR
(Collado et al. 2002)	(2S,1'S,2'S,3'R)-2-(2¢-carboxy-3'-methylcyclopropyl) glycine	NR	NR	PO, NR	8	NR	NR	NR	NR
(Johnson et al. 2005)	APPES	Rat, SDR	0.0001, 0.001, 0.01, 0.1	SC, NR	NR	NR	NR	0.1; ↓	NR
(Helton et al. 1998)	(-)-LY366563	Rat, LE	1, 3, 10	PO, 60	16	=	NR	=	NR
(Helton et al. 1998)	( +)-LY354740	Rat, LE	0.01, 0.03, 0.1, 1, 3, 10	PO, 60	16	=	NR	0.1, 1, 3, 10; ↓	NR
(Tizzano et al. 2002)	( +)-LY354740	Rat, SDR	0.3	IP, 30	8	NR	NR	0.3; ↓	NR
(Tizzano et al. 2002)	( +)-LY354740	Rat, SDR	0.003, 0.03, 0.3, 3	IP, 30	8	=	NR, data shown	0.3, 3; ↓	NR
(Tizzano et al. 2002)	( +)-LY354740	Rat, SDR	0.3	IP, 30	8	=	NR, data shown	0.3; ↓	NR
(Rorick-Kehn et al. 2007)	LY404039	Rat, SDR	0.03, 0.3, 3, 30 µg/kg	PO, 30	8	NR	NR	3, 30 µg/kg; ↓	NR
(Rorick-Kehn et al. 2007)	LY404039	Rat, SDR	30 µg/kg	PO, 30	8	NR	NR	30 µg/kg; ↓	NR
(Johnson et al. 2003)	LY487379	NR	0.03, 0.3, 3	IP, NR	NR	NR	NR	3; ↓	NR
(Johnson et al. 2005)	LY487379 (aka 4-MPPTS)	Rat, SDR	0.001, 0.01, 0.1, 1	SC, NR	NR	NR	NR	1; ↓	NR
*mGluR2,3 antagonists*									
(Tizzano et al. 2002)	LY341495	Rat, SDR	1	SC, 60	8	=	NR, data shown	=	NR
(Tizzano et al. 2002)	LY341495	Rat, SDR	1	SC, 60	8	=	NR, data shown	=	NR
(Rorick-Kehn et al. 2007)	LY341495	Rat, SDR	1	SC, 60	8	NR	NR	=	NR
(Johnson et al. 2003)	LY341495	NR	3	SC, NR	NR	NR	NR	=	NR
*GlyR partial agonists*									
Anthony & Nevins 1993)	1-aminocyclopropanecarboxylate	Rat, SDR	10, 30, 100, 200	IP, 15	8	=	NR; data shown	200; ↓	NR
Anthony & Nevins 1993)	1-aminocyclopropanecarboxylate	Rat, SDR	200, 300, 400, 500	IP, 15	8	=	NR; data shown	200, 300, 400, 500; ↓	NR
Anthony & Nevins 1993)	( +)-HA-966	Rat, SDR	1, 3, 10	IP, 15	8	=	NR; data shown	10, 30; ↓	NR
Anthony & Nevins 1993)	d-Cycloserine	Rat, SDR	10, 30, 100, 300	IP,15	8	=	NR; data shown	30, 100, 300; ↓	NR
(Anthony & Nevins 1993)	d-Cycloserine	Rat, SDR	10, 30, 100, 300	IP,15	8	=	NR; data shown	30, 100, 300; ↓	NR
(Walker et al. 2002)	d-Cycloserine	Rat, SDR	15	IP, 30	5 (6)	NR	NR	NR	=
*GlyR antagonists*									
(Anthony & Nevins 1993)	7-chlorokynurenate	Rat, SDR	10, 30, 100	IP, 15	8	=	NR; data shown	30, 100; ↓	NR
(Walker et al. 2002)	( ±)-HA-966	Rat, SDR	6	IP, 40	5 (6)	NR	NR	NR	=
*GluN receptor antagonists*									
Anthony & Nevins 1993)	2-amino-7-phosphonoheptanote	Rat, SDR	10, 30	IP, 15	8	=	NR; data shown	10, 30; ↓	NR
Anthony & Nevins 1993)	3-(2-carboxypiperazin-4-yl)propyl 1-phosphonate (CPP)	Rat, SDR	3	IP, 15	8	=	NR; data shown	3; ↓	NR
(Zhang et al. 2016)	Phencyclidine	Rat, SDR	1.5	IP, 10	10	NR	NR	NR	NR
Noradrenergic system									
*α*_*1*_*-adrenoreceptor antagonists*									
(Davis 1979)	WB4101	Rat, SDR	1	IP, 10	10	=	NR	NR	=
*α*_*2*_*-adrenoreceptor antagonists*									
(Risbrough & Geyer 2005)	Atipamezole HCL	Mouse, DBA/1 J	0.3, 1, 3	SC, 30	8 to 12	3; ↑	3; ↑	NR	=
(Davis 1979)	Piperoxane	Rat, SDR	0.25, 1	IP, 10	10	=	1; ↑	NR	1; ↑
(Bijlsma et al. 2010)	Yohimbine	Rat, Wi	0.25, 0.5, 1	IP, 10	11	=	0.25, 1; ↑	=	=
(Davis 1979)	Yohimbine	Rat, SDR	0.125, 0.25	IP, 10	10	=	0.25; ↑	NR	0,25; ↑
(Davis et al. 1988)	Yohimbine	Rat, SDR	5	IP, NR	5	NR	NR	NR	NR
(Risbrough & Geyer 2005)	Yohimbine	Mouse, DBA/1 J	0.1, 1, 10	SC, 30	8 to 12	=	NR; ↓	NR	10; ↓
*α*_*2*_*-adrenoreceptor agonists*									
(Davis 1979)	Clonidine	Rat, SDR	NA	IP, 10 µg/kg	NR	NR; ↓	NR; ↓	NR	NR; ↓
*β*_*1,2*_*-adrenoreceptor antagonists*									
(Davis 1979)	Propranolol	Rat, SDR	20	IP, 10	10	=	NR	NR	=
Dopaminergic system									
*D*_*1*_* receptor agonists*									
(de Oliveira et al. 2006)	SKF 38,393	Rat, Wi	5, 10	IP, 10	20	=	=	NR	NR
*D*_*1*_* receptor antagonists*									
(de Oliveira et al. 2006)	SCH 23,390	Rat, Wi	0.05, 0.1	IP, 30	20	=	=	NR	NR
*D2 receptor agonists*									
(de Oliveira et al. 2006)	Quinpirole	Rat, Wi	0.1, 0.25	IP, 10	15	=	0.1, 0.25; ↓	NR	NR
*D*_*2*_* receptor antagonists*									
(Hijzen et al. 1995)	Haloperidol	Rat, Wi	0.065, 0.125, 0.25	IP, 30	12	=	NR; ↓; cue x dose	NR	NR
Muthuraju 2014	Haloperidol	Rat, Wi	0.1, 0.5	IP, 5	10–17	=	0.1, 0.5; ↓	NR	NR
(Nevins & Anthony 1994)	Haloperidol	Rat, SDR	0.1, 0.3	IP, NR	8	=	NR; data shown	=	NR
(de Oliveira et al. 2006)	Sulpiride	Rat, Wi	20, 40	IP, 10	15	=	=	NR	NR
*Psychostimulants*									
(Vale & Green 1996)	d-Amphetamine	Rat, LH	0.5, 1	IP, 30	11–12	=	=	NR	NR
(Hijzen et al. 1995)	d-Amphetamine	Rat, Wi	0.6, 1.2, 2.4	IP, 15	12	=	NR; ↓; cue x dose	NR	NR
Opioid system									
*μ receptor partial agonists*									
(Glover & Davis 2008)	Buprenorphine	Rat, SDR	0.004, 0.0075, 0.015, 0.03, 0.25	SC, 0	45 (NR)	=	NR; ↓	0.0075, 0.015, 0.03, 0.25; ↓	NR
*μ receptor agonists*									
(Davis 1979)	Morphine	Rat, SDR	0.63, 2.5, 10	IP, 10	8	=	NR; ↓	NR	NR
(Davis 1979)	Morphine	Rat, SDR	10	IP, 10	8	=	10; ↓	NR	NR
(Glover & Davis 2008)	Morphine	Rat, SDR	0.03, 0.25, 0.63, 2.5, 10	SC, 0	45 (NR)	=	NR; ↓	0.63, 2.5, 10; ↓	NR
(Hijzen et al. 1995)	Fentanyl	Rat, Wi	0.0025, 0.01, 0.04	IP, 30	12	=	=	NR	NR
*μ receptor antagonists*									
(Davis 1979)	Naloxone	Rat, SDR	2	IP, 20	8	=	=	NR	NR
(Davis et al. 1988)	Naloxone	Rat, SDR	5	NR, NR	5	NR	NR	NR	NR
(Hijzen et al. 1995)	Naloxone	Rat, Wi	2.5, 5, 10	IP, 25	12	=	=	NR	NR
(Glover & Davis 2008)	Naloxone	Rat, SDR	0.5	SC, 5	NR	NR	NR	=	NR
Neuropeptides									
*BB1,2 receptor antagonists*									
(Merali et al. 2006)	PD176252	Rat, NR	5, 10	IP, 20	9	=	5, 10; ↓	NR	NR
Cholecystokinin system									
*CCK*_*2*_* receptor agonists*									
(Hebb et al. 2003)	Boc CCK-4	Mouse, CD1	5, 15 µg/kg	SC, 30	5	=	15; ↓	NR	NR
*CCK*_*2*_* receptor antagonists*									
(Josselyn et al. 1995)	L-365,260	Rat, Wi	0.1, 1, 10	IP, 15	16 (11)	=	1, 10; ↓	NR	NR
CRF system									
*CRF*_*1*_* receptor antagonists*									
(Bijlsma et al. 2015)	CP154,526	Rat, Wi (wt)	10 µg/kg	IP, 30	9–10	NR; ↓; main effect	no cue x dose	NR	NR
(Chen et al. 1997)	CP-154,526	Rat, SDR	17.8	PO, 60	NR (12)	=	NR	17.8; ↓	NR
(Risbrough et al. 2009)	NBI-30775	Mouse, C57BL/6 J	20	IP, 30	7–13	NR	NR	NR	=
*Ghrelin-R1a agonist*									
(Toufexis et al. 2016)	Ghrelin	Rat, SDR male	10	SC, 20	16	=	NR	NR	=
(Toufexis et al. 2016)	Ghrelin	Rat, SDR female	10	SC, 20	16	=	NR	NR	=
*NOP receptor agonists*									
(Varty et al. 2008)	SCH 221,510	Rat, Wi	3, 6, 10	PO, 120	12–14	=	NR	NR	NR; ↓
(Jenck et al. 2000)	Ro 64–6198	Rat, Wi	1, 3.2, 10	IP, 30	16	3.2, 10; ↓	3.2, 10; ↓	NR	NR
(Lu et al. 2011)	SCH 655,842	Rat, Wi	1, 3, 10	PO, 60	12–15	=	NR	NR	3, 10; ↓
*NTS*_*1*_* receptor agonists*									
(Shilling & Feifel 2008)	PD149163	Rat, SDR	0.01, 0.1, 1.0	SC, 20	7–10	=	NR	NR	1; ↓
(Shilling & Feifel 2008)	PD149163	Rat, SDR	1	SC, 20	16	1; ↓	NR	NR	1; ↓
*OX*_*1,2*_* receptor antagonists*									
(Steiner et al. 2012)	Almorexant	Rat, F344	30, 100, 300	PO, 60	12	300; ↓	100, 300; ↓	NR	NR
*OX*_*1*_* receptor antagonists*									
(Steiner et al. 2013)	ACT-335827	Rat, F344	30, 100, 300	PO, 60	16	=	300; ↓	NR	NR
*OT receptor agonists*									
(Ayers et al. 2011)	Oxytocin	Rat, SDR	0.01, 0.1, 1.0 µg/kg	SC, 30	12	0.001, 0.1; ↓	=	NR	=
(Ayers et al. 2016)	Oxytocin	Rat, SDR	0.1	SC, 30	55 (54)	NR; ↓; main effect	no cue x dose	NR	=
(Missig et al. 2010)	Oxytocin	Rat, SDR	0.01, 0.1, 1 µg/kg	SC, 30	12	NR; ↓; main effect	no cue x dose	NR	=
*Secretin receptor agonists*									
(Myers et al. 2004)	Secretin	Rat, SDR	1, 3, 10, 30, 100 µg/kg	IP, 0	16 (8–12)	=	NR	10; ↓	=
Miscellaneous									
Cholinergic system									
*nACh receptor agonists*									
(Hijzen et al. 1995)	Nicotine	Rat, Wi	0.4, 0.8, 1.6	IP, 15	12	=	=	NR	NR
(Vale & Green 1996)	Nicotine	Rat, LH	0.05, 0.1, 0.4	SC, 0	10–12	=	=	NR	NR
(Vale & Green 1996)	Nicotine	Rat, LH	0.1	SC, 0	10	=	=	NR	NR
Endocannabinoid system									
*CB*_*1*_* receptor antagonists*									
(Chhatwal et al. 2005)	Rimonabant	Rat, SDR	5	IP, 60	8	NR	NR	NR	=
*Endocannabinoid reuptake inhibition*									
(Chhatwal et al. 2005)	AM404	Rat, SDR	10	IP, 60	8	=	NR	NR	=
Glucocorticoid system									
*MR/GR receptor agonists*									
(de Oliveira et al. 2013)	Corticosterone	Rat, Wi	3, 6	IP, 0	6 (12)	=	=	NR	NR
*Corticosterone synthesis blocker*									
(de Oliveira et al. 2013)	Metyrapone	Rat, Wi	30, 60	IP, 20	6 (12)	=	=	NR	NR
Other									
*Voltage dependent sodium channel blocker*									
(Hijzen et al. 1995)	Carbamazepine	Rat, Wi	5, 10, 20	IP, 60	12	=	=	NR	NR

*FPS* fear potentiation, *IP* intraperitoneally, *ITI* inter-trial interval, *LE* Long Evans, *NR* not reported, *PO* per os, *SC* subcutaneously, *SDR* Sprague–Dawley rat, *Wi* Wistar, *wt* wild type, *CRF* corticotropin-releasing factor, *GluN* ionotropic glutamate receptor, *GlyR* glysine receptor, *m-CPP* m-chlorophenylpiperazine, *mGlu* metabotropic glutamate, *MR/GR* mineralocorticoid receptor/glucocorticoid receptor, *nACh* nicotinic acetylcholine, *NOP* nociceptin opioid peptide, *NTS* neurotensin, *OT* oxytocin, *OX* orexin, ↓ response reduced relative to vehicle control condition, ↑ response increased relative to vehicle control condition, *cue* × *dose* interaction effect in ANOVA

(*) group size of intervention group is given in brackets if the group size of the experimental group differed from that of the vehicle-control group

Regarding experimental compounds, 13 compounds from 8 different GABA-ergic drug classes were tested in 17 experiments (see Table 3). Drug classes included the GABA_A_ receptor partial agonists (two experiments, two different drugs), the GABA_A_ receptor α1 subunit agonists (one experiment, one drug), the GABA_A_ receptor α2,3 subunit agonists (two experiments, one drug), the GABA_A_ receptor α5 subunit selective antagonists (one experiment, one drug), the GABA_A_ receptor agonist alcohol (one experiment), the GABA_A_ receptor antagonists (two experiments, one drug), and the GABA_B_ receptors (one experiment, one drug).

Experimental drug classes further included the GABA_A_ inverse receptor agonists (also known as GABA_A_ negative allosteric modulators), which are presumed anxiogenic drugs (five experiments, four drugs).

For 12 experiments, outcome data on fear potentiation was incomplete, whereas reporting of non-cued baseline startle-response data was incomplete for 16 experiments.

##### Effects of clinically used GABA-ergic compounds in the fear-potentiated startle

**Fig. 4:**
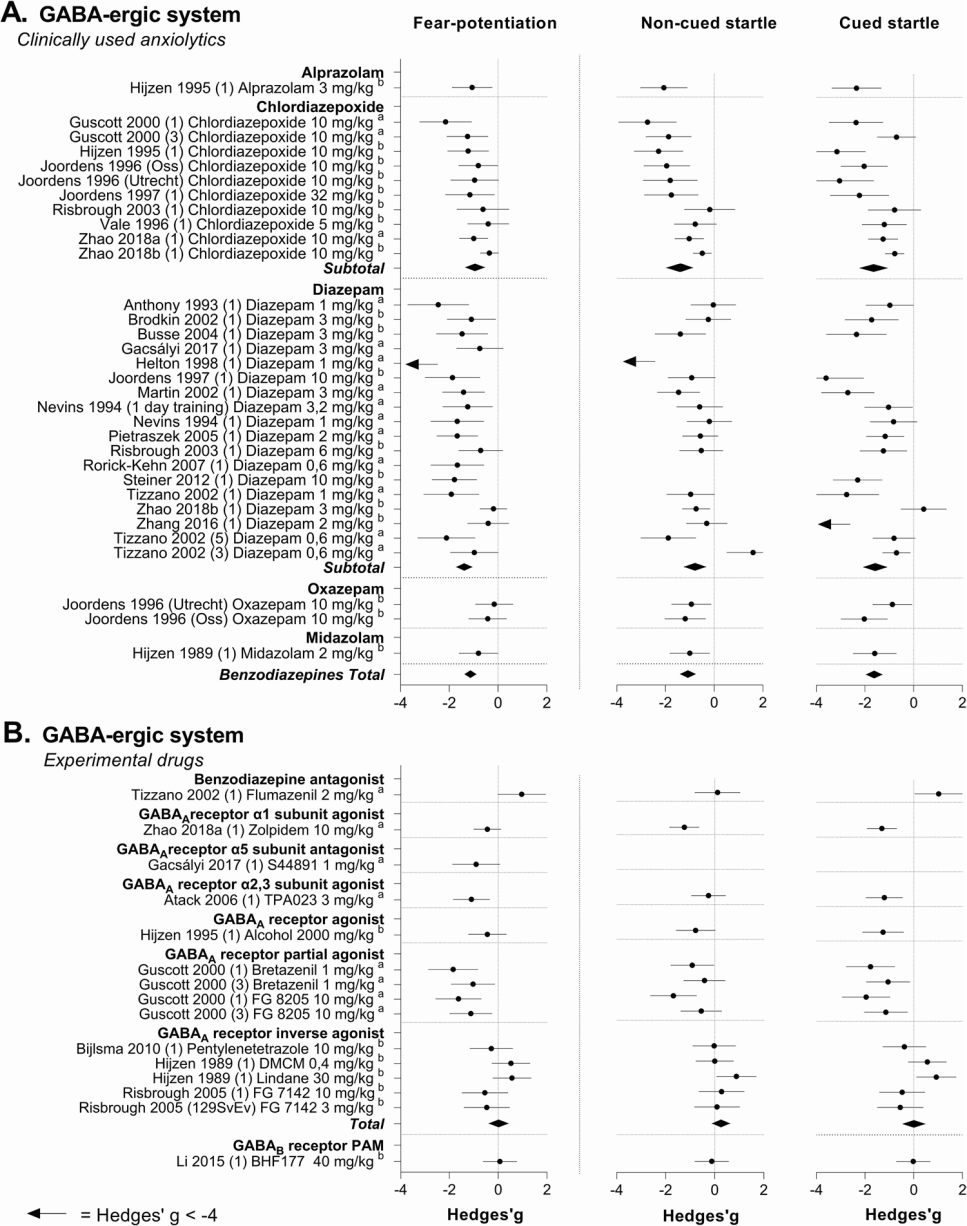
Forest plots of the effects of clinically used (**A**) and experimental (**B**) GABA-ergic drugs on fear potentiation (left), non-cued baseline startle response (middle), and the response to cued startle trials (right). Data are shown as Hedge’s *g* and 95% confidence intervals. Pooled effect sizes, shown as *Total*, were calculated per drug class. If data were insufficient to conduct a meta-analysis, data were grouped but not pooled. Pooled effects of subgroup analysis are shown as *Subtotal*. Fear potentiation represents ^a^ = difference score, ^b^ = calculated difference score, ^c^ = % fear-potentiated startle

As shown in Fig. 4a, the effects of benzodiazepines on fear potentiation and the non-cued baseline startle response were rather consistent. Pooling of the data showed that benzodiazepines as a drug class significantly reduced both fear potentiation (SMD − 1.13 [− 1.37, − 0.88], *I*^2^ = 59.3%) and the startle response to non-cued trials relative to vehicle treatment (SMD − 1.08 [− 1.39, − 0.76], *I*^2^ = 74%). Effect size estimates were rather consistent and the proportion of between-study heterogeneity was moderate for fear potentiation and high for the non-cued baseline startle response (Table 4).

**Table 4:** Summary statistics of drug classes included in the meta-analysis for fear-potentiation and the non-cued startle baseline response

Drug classes	Articles	Experiments	Animals	SMD [95% CI]	*I*^2^ (%)
*Fear potentiation*					
GABA-ergic system					
Benzodiazepines	22	32	762	− 1.13 [− 1.37, − 0.88]	59.3
GABA_A_ receptor inverse agonists	3	5	98	0.01 [− 0.48, 0.49]	36
Serotonergic system					
Buspirone	6	7	134	− 1.72 [− 1.50, − 0.74]	86
5-HT_1A_ receptor agonists	3	7	260	− 1.1 [− 1.4, − 0.77]	30
5-HT1A receptor antagonists	3	5	110	− 0.7 [− 1.23, − 0.25]	39
Glutamatergic system					
mGluR2,3 agonists	3	7	102	− 1.71 [− 2.59, − 0.83]	73
*Non-cued startle baseline response*					
GABA-ergic system					
Benzodiazepines	22	29	714	− 1.08 [− 1.39, − 0.76]	74
GABA_A_ receptor inverse agonists		5	98	0.26 [− 0.13, 0.64]	0
Serotonergic system					
Buspirone	6	7	134	0.36 [− 0.40, 1.13]	80
5-HT_1A_ receptor agonists	3	7	256	− 0.21 [− 0.69, 0.28]	72
5-HT1A receptor antagonists	3	5	110	− 0.65 [− 1.02, − 0.28]	0
Glutamatergic system					
mGluR2,3 agonists	1	2	32	Not pooled	

Drug classes were omitted from the analysis if these contained fewer than five experiments, from fewer than three different articles

*SMD* standardized mean difference (Hedge’s *g*), *CI* confidence interval

Diazepam and chlordiazepoxide were eligible for subgroup analysis on drugs. Both benzodiazepines significantly reduced the level of fear-potentiation (Fig. 4, Table 5; diazepam SMD − 1.42 [− 1.81, − 1.03], *I*^2^ = 64%; chlordiazepoxide SMD − 0.91 [− 1.23, − 0.58], *I*^2^ = 41.8%)). Pooled effect sizes and heterogeneity were moderate. Diazepam (SMD − 0.79 [− 1.25, − 0.33], *I*^2^ = 77%) and chlordiazepoxide (SMD − 1.43 [− 1.98, − 0.87], *I*^2^ = 74%) also considerably reduced the non-cued baseline startle response (Fig. 4; Table 6). Effect size estimates were comparable between the two drugs.

**Table 5:** Subgroup statistics for the effects of benzodiazepines on fear potentiation in rats

	Articles	Experiments	Animals	SMD [95% CI]	*I*^2^ (%)
All experiments	21	30	732	− 1.16 [− 1.42, − 0.90]	62
Drugs					
Alprazolam	1	1	24	Not pooled	
Chlordiazepoxide	7	9	300	− 0.97 [− 1.42, − 0.53]	48
Diazepam	14	17	336	− 1.44 [− 1.79, − 1.08]	65
Midazolam	1	1	24	Not pooled	
Oxazepam	1	2	48	Not pooled	
Strain					
F344	1	1	24	Not pooled	
HL	1	1	20	Not pooled	
LE	1	1	16	Not pooled	
RORO	1	1	24	Not pooled	
SDR	8	12	220	− 1.44 [− 1.78, − 1.10]	30
Wi	9	14	428	− 0.78 [− 1.04, − 0.52]	35
Time of testing					
Active period	2	3	176	Not pooled	
Passive period	13	18	384	− 1.10 [− 1.35, − 0.84]	30
NR	6	9	172	− 1.70 [− 2.26, − 1.14]	61
Shock intensity (mA)					
High (≥ 0.8)	4	5	80	− 1.70 [− 2.41, − 1.00]	76
Low (< 0.3)	3	3	60	Not pooled	
Moderate (0.3 ≤ intensity < 0.8)	14	22	592	− 1.09 [− 1.38, − 0.79]	58
Total number of cue-shock pairings					
High (> 10 pairings)	17	23	460	− 1.26 [− 1.55, − 0.98]	52
Moderate (3–10 pairings)	4	7	272	− 0.84 [− 1.31, − 0.37]	66
Low (< 3 pairings)	0	0	0	NA	NA
Highest startle probe intensity (dB; calculated relative to background noise)					
High (> 35)	14	20	568	− 0.97 [− 1.23, − 0.71]	53
Moderate (20 ≤ intensity < 35)	3	4	68	Not pooled	
Low (< 20)	0	0	0	NA	
NA (intensity not reported)	4	6	96	− 1.88 [− 2.74, − 1.03]	69
Study design					
Between-subjects	16	21	416	− 1.37 [− 1.65, − 1.09]	49
Within-subjects (Latin square)	4	9	316	− 0.70 [− 1.08, − 0.33]	49
*Quality indicators*					
Blinding reported					
No	19	27	528	− 1.26 [− 1.52, − 1.00]	51
Yes	1	1	48	Not pooled	
Yes. Not blinded	1	2	156	Not pooled	
Sample size calculation					
No	20	28	576	− 1.24 [− 1.49, − 0.99]	49
Yes	1	2	156	Not pooled	
Allocation bias					
No measures reported	8	9	280	− 1.01 [− 1.47, − 0.54]	77
Random allocation	4	5	124	− 1.20 [− 1.81, − 0.58]	39
Matching (any kind)	8	16	328	− 1.23 [− 1.59, − 0.88]	42

Subgroups were omitted from the analysis if these contained fewer than five experiments, from fewer than three different articles

*SMD* standardized mean difference (Hedge’s *g*), *CI* confidence interval, *F344* Fischer 344, *LE* Lewis, *LH* Lister Hooded, *NA* not applicable, *RORO* Fuellinsdorf Albino, *SDR* Sprague–Dawley, *Wi* Wistar

**Table 6:** Subgroup statistics for the effects of benzodiazepines on the non-cued baseline startle response in rats

	Articles	Experiments	Animals	SMD 95% CI	*I*^2^ (%)
All experiments	17	27	684	− 1.13 [− 1.47, − 0.80]	75
Drugs					
Alprazolam	1	1	24	Not pooled	
Chlordiazepoxide	7	9	300	− 1.56 [− 2.16, − 0.96]	75
Diazepam	11	14	288	− 0.81 [− 1.30, − 0.32]	79
Midazolam	1	1	24	Not pooled	
Oxazepam	1	2	48	Not pooled	
Strain					
F344	1	1	24	Not pooled	
HL	1	1	20	Not pooled	
LE	0	0	0	NA	
RORO	1	1	24	Not pooled	
SDR	6	10	188	− 0.72 [− 1.22, − 0.22]	79
Wi	8	14	428	− 1.22 [− 1.63, − 0.81]	56
Time of testing	2	3	176	Not pooled	
Active period	11	17	368	− 1.22 [− 1.65, − 0.78]	73
Passive period	4	7	140	− 1.18 [− 1.87, − 0.49]	83
NR					
Shock intensity (mA)					
High (≥ 0.8)	1	2	32	Not pooled	
Low (< 0.3)	3	3	60	Not pooled	
Moderate (0.3 ≤ intensity < 0.8)	14	22	592	− 1.14 [− 1.53, − 0.76]	79
Total number of cue-shock pairings					
High (> 10 pairings)	13	20	412	− 1.35 [− 1.73, − 0.97]	71
Moderate (3–10 pairings)	4	7	272	− 0.56 [− 1.17, − 0.04]	75
Low (< 3 pairings)	0	0	0	NA	
Highest startle probe intensity (dB; calculated relative to background noise)					
High (> 35)	11	18	536	− 1.46 [− 1.85, − 1.06]	74
Moderate (20 ≤ intensity < 35)	3	4	68	Not pooled	
Low (< 20)	0	0	0	NA	
NA (intensity not reported)	3	5	80	− 0.57 [− 1.35, 0.21]	83
Study design					
Between-subjects	13	18	368	− 0.88 [− 1.30, − 0.46]	67
Within-subjects (Latin square)	4	9	316	− 1.64 [− 2.23, − 1.05]	84
*Quality indicators*					
Blinding reported					
No	15	24	480	− 1.20 [− 1.58, − 0.82]	76
Yes	1	1	48	Not pooled	
Yes, not blinded	1	2	156	Not pooled	
Sample size calculation					
No	16	25	528	− 1.19 [− 1.54, − 0.83]	75
Yes	1	2	156	Not pooled	
Allocation bias					
No measures reported	5	6	232	− 0.83 [− 1.53, − 0.13]	26
Random allocation	4	5	124	− 1.56 [− 2.38, − 0.73]	87
Matching (any kind)	8	16	328	− 1.14 [− 1.59, − 0.69]	76

Subgroups were omitted from the analysis if these contained fewer than five experiments, from fewer than three different articles

*SMD* standardized mean difference (Hedge’s *g*), *CI* confidence interval, *F344* Fischer 344, *LE* Lewis, *LH* Lister Hooded, *NA* not applicable, *RORO* Fuellinsdorf Albino, *SDR* Sprague–Dawley, *Wi* Wistar

A subgroup analysis comparing rat strains showed that the pooled effect of benzodiazepines on fear potentiation was larger in Sprague–Dawley rats (Table 5; SMD − 1.44 [− 1.78, − 1.10], *I*^2^ = 30%) than in Wistar rats (SMD − 0.78 [− 1.03, − 0.52], *I*^2^ = 35%). In both subgroups, heterogeneity was low, relative to that observed for the overall effect of benzodiazepines. The effect of benzodiazepines on the non-cued baseline startle response, however, did not seem dependent on the rat strain used (Table 6).

Subgroup analysis for the other pre-defined moderators did not indicate that the effects of benzodiazepine on fear potentiation or non-cued baseline startle response were dependent on the time of testing, shock intensity, the total number of cue-shock pairings, startle noise intensity, study design, or quality indicators (Tables 4 and 5, respectively).

##### Effects of experimental GABA-ergic compounds in the fear-potentiated startle test

Regarding the experimental drugs (Fig. 4b), it is interesting to note that the GABA_A_ receptor partial agonists like benzodiazepines also reduced fear potentiation and tended to reduce the non-cued baseline startle response.

The effect size estimates for the GABA_A_ receptor inverse agonists showed a different pattern. These anxiogenic drugs had no marked effect on the level of fear potentiation and tended to increase the non-cued baseline startle response. This pattern was reflected in the meta-analyses (Table 4) that showed that GABA_A_ receptor inverse agonists had no effect on fear potentiation (SMD 0.01 [− 0.48, 0.49], *I*^2^ = 36%) and enhanced the non-cued baseline startle response, but not significantly (SMD 0.26 [− 0.13, 0.64], *I*^2^ = 0%).

Data was too limited to perform a meta-analysis on the other experimental GABA-ergic drug classes.

##### Publication bias

Visual inspection of the funnel plot for effects on fear potentiation (Supplementary File 7) suggests that the plot is asymmetrical due to a low number of small studies with medium to large effect sizes. This observation is confirmed by Egger’s test for small-study effects (*p* = 0.004) and trim-and-fill analysis. Trim-and-fill analysis imputed 11 experiments and shifted the pooled effect size to the left (SMD − 0.76 [− 1.15, − 0.37]. For the non-cued baseline startle response, the appearance of the funnel plot (Supplementary File 7), Egger’s test for small-study effects (*p* = 0.54), and trim-and-fill analysis did not suggest the presence of publication bias.

##### Sensitivity analysis

We performed two sensitivity analyses, one for pooling experiments with rats and mice and one for pooling reported and calculated difference scores.

Removing the two mouse studies from the dataset had no effect on the direction of magnitude of the pooled effect for benzodiazepines (fear potentiation SMD − 1.16 [− 1.42, − 0.90], *I*^2^ = 61.6%, 30 experiments; non-cued baseline startle response SMD =  − 1.13 [− 1.47, − 0,80], *I*^2^ = 75%, 27 experiments) nor on the pooled effect sizes for diazepam and chlordiazepoxide in the subgroup analysis. Given the marked differences in experimental setup between rat and mouse studies (Supplementary File 4), we did not include the mouse studies in the subgroup analyses for methodological characteristics. Sensitivity analyses showed that the inclusion of the two mouse studies in the different subgroup analyses did not alter the outcome of these analyses.

Excluding experiments with calculated difference scores from the analysis did not alter the direction or significance of the effects of benzodiazepines on fear potentiation (pooled effect size for experiments with reported difference scores only: SMD − 1.55 [− 1.9, − 1.17], *I*^2^ = 48%, 13 experiments).

In conclusion, most drugs interacting with the GABA_A_ receptor seem to alter the level of fear potentiation and the magnitude of the non-cued baseline startle response. Anxiolytic-like drugs reduce these responses, whereas anxiogenic-like drugs may tend to enhance the responses.

#### Serotonergic system

SSRIs are the first-line pharmacological treatment for anxiety disorders. Although SSRIs are not effective in all patients or may leave patients with residual symptoms (Baldwin et al. 2014), their general efficacy indicates that drugs that act on the serotonergic system may alter the levels of anxiety in humans. Depending on the serotonin (5-HT) receptor subtype that is activated, this could result in anxiolytic effects, e.g., in the case of the 5-HT_1A_ receptor partial agonists buspirone, or in anxiogenic effects, as has been shown for the 5-HT_2C_ receptor agonist m-CPP (Charney et al. 1987).

##### Study characteristics for the serotonergic system

So far, 25 serotonergic drugs have been tested in a total of 52 experiments. These drugs were categorized into ten drug classes based on their mechanism of action (Table 3). Regarding clinically used anxiolytics, three different drug classes were tested, in a total of 15 experiments. Drug classes included the 5-HT_1A_ receptor partial agonists, represented by buspirone which was tested in eight experiments, the SSRIs (three experiments, three drugs), and the tricyclic antidepressants (TCAs; three experiments, three drugs).

Regarding the experimental drugs, 37 experiments were performed with 19 drugs from seven drug classes. The nine 5-HT_1A_ receptor ligands were categorized into four drug classes. Drug classes included the 5-HT_1A_ receptor partial agonists (two experiments, two drugs), the 5-HT_1A_ receptor biased agonists (two experiments, two drugs), the 5-HT_1A_ receptor agonists (eight experiments, two drugs), and the 5-HT_1A_ receptor antagonists (five experiments, three drugs). The other drug classes that were tested were the 5-HT_2A,2C_ receptor antagonists (four experiments, four drugs), the 5-HT_3_ receptor antagonists (ten experiments, three drugs), and 5-HT releasers (one experiment, one drug). Finally, the 5-HT_2C_ receptor agonist m-CPP, which is known for its anxiogenic effects in humans and animals (Charney et al. 1987; Willadsen et al. 2018), was tested in three experiments.

For six experiments, outcome data on fear potentiation was incomplete, whereas reporting of non-cued baseline startle response data was incomplete for seven experiments.

##### Effects of clinically used serotonergic drugs in the fear-potentiated startle test

**Fig. 5:**
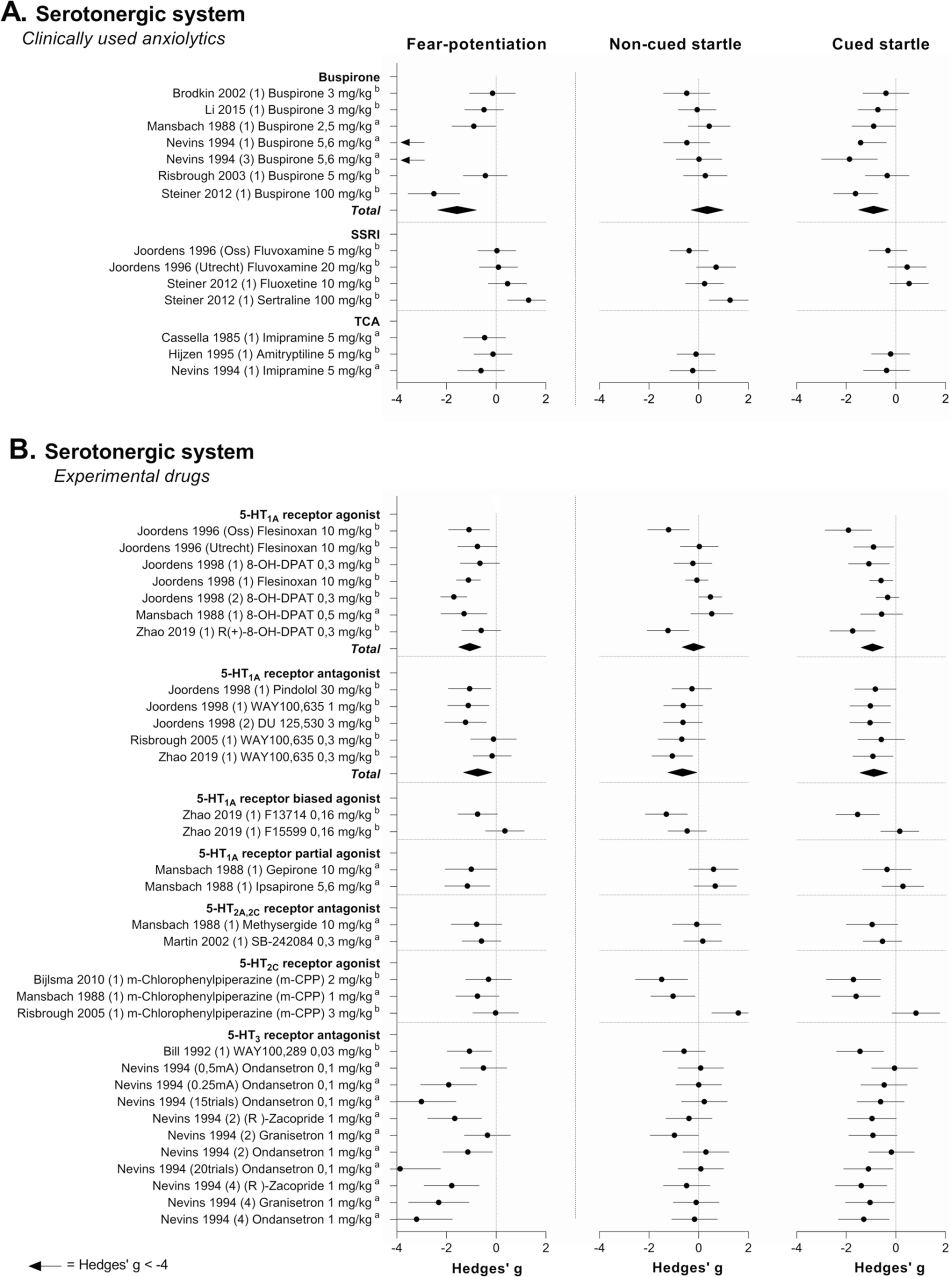
Forest plots of the effects of clinically used anxiolytics (**A**) and experimental (**B**) serotonergic drugs on fear potentiation (left), non-cued baseline startle response (middle), and the response to cued startle trials (right). Pooled effect sizes, shown as *Total*, were calculated per drug class. If data were insufficient to conduct a meta-analysis, data were grouped but not pooled. Data are shown as Hedge’s *g* and 95% confidence intervals. Fear potentiation represents ^a^ = difference score, ^b^ = calculated difference score, ^c^ = % fear-potentiated startle

As shown in Fig. 5a, effect size estimates for the registered anxiolytics varied between drug classes. Effects for buspirone ranged from null effects to marked anxiolytic effects. Meta-analysis showed that the registered anxiolytic buspirone significantly reduced fear potentiation (SMD − 1.72 [− 1.50, − 0.74], *I*^2^ = 86%), whereas buspirone had no effect on the non-cued baseline startle response (SMD 0.36 [− 0.40, 1.13], *I*^2^ = 80%; Table 4).

Acute administration of SSRIs and TCAs predominantly yielded null effects. In the experiments with SSRIs, point estimates were mostly small and positive relative to zero. There were not enough experiments to conduct a meta-analysis of the data obtained for SSRIs and TCAs.

##### Effects of experimental serotonergic drugs in the fear-potentiated startle test

Figure 5b shows the effect of the experimental serotonergic compounds on fear potentiation and on the non-cued baseline startle response. Effect size estimates for the 5-HT_3_ receptor antagonists on fear potentiation were relatively large. All 5-HT_3_ receptor antagonists except for WAY100289 were, however, tested within the scope of a single study. Therefore, it is difficult to generalize these data or to substantiate these findings with a meta-analysis.

Interestingly, the effects of the anxiogenic drug m-CPP on fear potentiation in rats were in the same direction as the effects of clinically used anxiolytics. Data were insufficient to statistically analyze the effect of m-CPP.

Meta-analysis showed that 5-HT_1A_ receptor agonists reduced fear potentiation (SMD − 1.1 [− 1.4, − 0.77], *I*^2^ = 30%) and did not alter the non-cued baseline startle response (SMD − 0.21 [− 0.69, 0.28], *I*^2^ = 72%). This profile is similar to that observed for the partial 5-HT_1A_ receptor agonist buspirone (Table 4).

5-HT_1A_ receptor antagonists on the other hand reduced both fear potentiation and the non-cued baseline startle response, but the effect sizes were rather small (fear potentiation SMD − 0.7 [− 1.23, − 0.25], *I*^2^ = 39%; non-cued baseline startle 5-HT_1A_ antagonists SMD − 0.65 [− 1.02, − 0.28], *I*^2^ = 0%, respectively; Table 4).

##### Sensitivity analysis

A sensitivity analysis to control for pooling data from rat and mouse experiments showed that excluding the mice experiments did not alter the substantive interpretation of the overall effects (buspirone, fear potentiation SMD − 1.99 [− 3.25, − 0.73], *I*^2^ = 88%, six experiments; non-cued baseline startle response SMD 0.39 [− 0.53, 1.30], *I*^2^ = 29%, six experiments; 5-HT_1A_ antagonists, fear potentiation SMD − 0.88 [− 1.38, − 0.37], *I*^2^ = 33%, four experiments; non-cued baseline startle SMD − 0.64 [− 1.05, − 0.24], *I*^2^ = 0%, four experiments).

In sum, data synthesis indicated that ligands interacting with the 5-HT_1A_ receptor reduce fear potentiation, in the absence of substantial effects on the startle response to non-cued trials. Remarkably, this anxiolytic effect was observed for 5-HT_1A_ agonists, partial agonists as well as antagonists. Acute treatment with either SSRIs or TCAs, however, did not reduce fear expression. In fact, the limited data conversely point toward a potential increase in startle response following acute treatment with SSRIs. Available data for the other drug classes suggest that 5-HT_3_ receptors may modulate the expression of conditioned fear, whereas for 5-HT_2_ receptor agonists and antagonists, this seems less likely.

#### Glutamatergic system

Currently, there are no glutamatergic drugs in clinical use for the treatment of anxiety. However, in the search for novel treatment strategies, the glutamatergic system has received considerable attention given its close interaction with GABA-ergic and serotonergic systems within corticolimbic projections (Sartori and Singewald 2019; Spooren et al. 2003). Over the years, a wide range of metabotropic and ionotropic receptor ligands has been synthesized and tested in preclinical animal tests (Dogra and Conn 2021; Nasir et al. 2020).

##### Study characteristics for the glutamatergic system

In the fear-potentiated startle test, 22 experimental drugs have been tested in 38 experiments. These drugs were categorized into seven drug classes based on their mechanism of action. Four of these drug classes act on metabotropic glutamate receptors. Drug classes included the mGLuR1 receptor antagonists (one experiment, one drug), mGluR5 antagonists (nine experiments, five drugs), mGluR2,3 receptor agonists (ten experiments, seven drugs), and mGluR2,3 antagonists (three experiments, one drug). The other three drug classes that were studied acted on ionotropic glutamate receptors. Drug classes included GlyR partial agonists (eight experiments, three drugs), GlyR antagonists (two experiments, two drugs), and GluN antagonists (three experiments, three drugs).

For 10 experiments, data on fear potentiation was not reported. Data on the non-cued baseline startle response after drug treatment was not fully reported for 17 experiments.

**Fig. 6:**
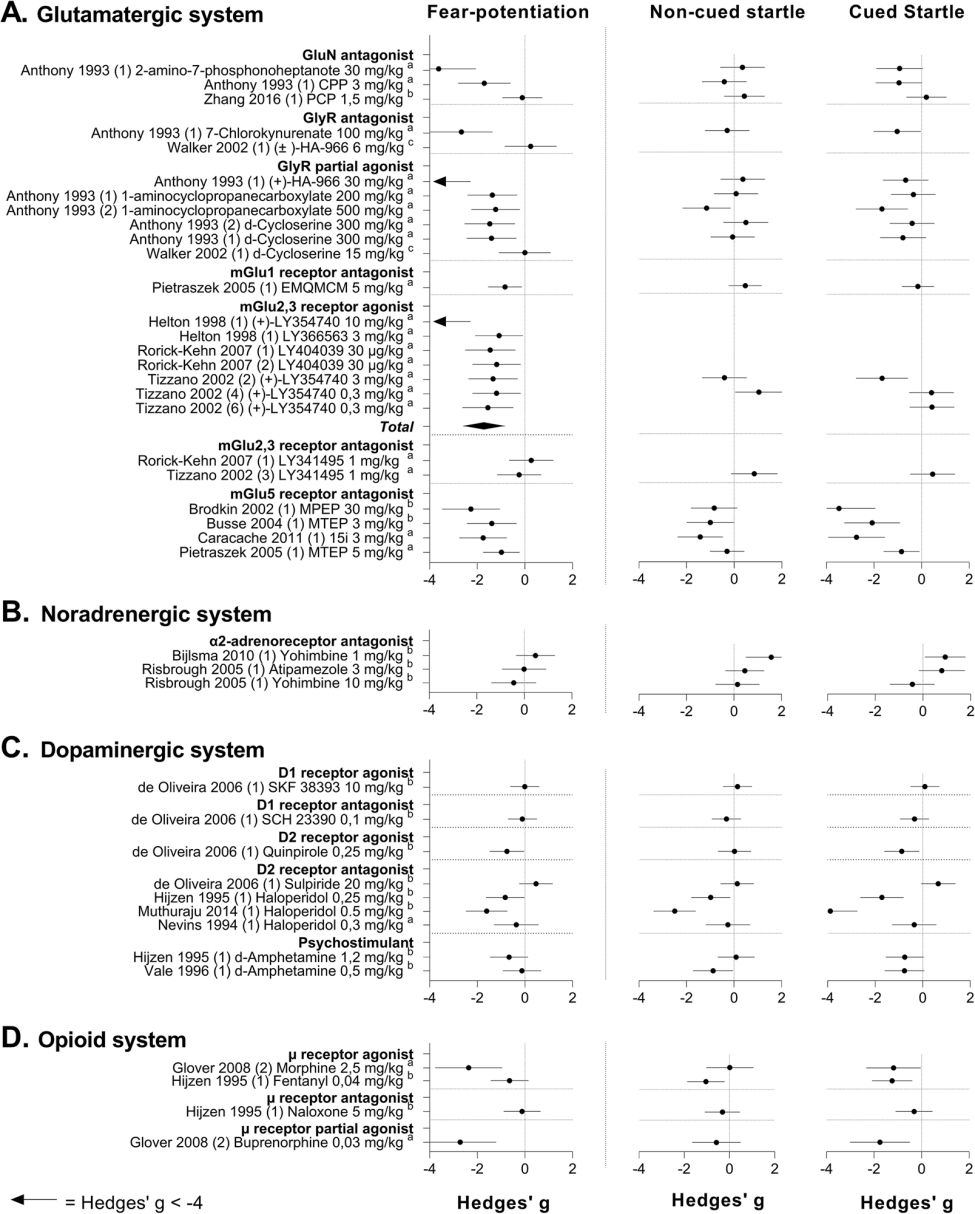
Forest plots of the effects of glutamatergic (**A**), noradrenergic (**B**), dopaminergic (**C**), and opioid drugs (**D**) on fear potentiation (left), non-cued startle response (middle), and the response to cued startle trials (right), sorted by the mechanism of action. Pooled effect sizes, shown as *Total*, were calculated per drug class. If data were insufficient to conduct a meta-analysis, data were grouped but not pooled. Data are shown as Hedge’s *g* and 95% confidence intervals. Fear potentiation based on ^a^ = difference score, ^b^ = calculated difference score, ^c^ = % fear-potentiated startle

Figure 6a shows the effect size estimates for the glutamatergic compounds that have been tested. GlyR partial agonists, mGluR2,3 agonists, and mGluR5 receptor antagonists each consistently reduced fear potentiation. Whereas the effects of the partial GlyR antagonists on the non-cued startle baseline were dispersed around zero, effect size estimates for the mGluR5 receptor antagonists were negative relative to zero, but predominantly non-significant. Remarkably, effects of mGluR2,3 agonists on the non-cued startle response was only reported for two of the seven experiments. For drugs that were hypothesized not to alter fear expression, e.g., the mGluR2,3 antagonist LY341495 and the GlyR antagonist ( ±)-HA-966, null effects on fear potentiation were reported.

Meta-analysis showed that mGluR2,3 agonists significantly reduced fear potentiation (SMD − 1.71 [− 2.59, − 0.83], *I*^2^ = 73%; Fig. 6a, Table 4). Data were insufficient to conduct a meta-analysis on the effect of mGluR2,3 agonists on the non-cued startle response.

Overall, data suggest that mGluR2,3 agonists as well as GlyR partial agonists and mGluR5 antagonists may reduce fear potentiation and that the magnitude of the effect does not seem to differ between these drug classes. Additional studies would, however, be necessary to confirm these observations.

#### Noradrenergic system

The noradrenergic system may modulate various forms of anxiety via α- and β-adrenoceptors. The β-adrenoceptor antagonist propranolol has been used for the treatment of performance anxiety and social anxiety disorders (Garakani et al. 2020). The α_2_-adrenoceptor agonist clonidine may also exert beneficial effects (Hoehn-Saric et al. 1981), but its clinical use is very limited (Garakani et al. 2020). The α_1_-adrenoceptor agonist prazosin has been shown to alleviate symptoms of PTSD (Raskind et al. 2003; Reist et al. 2021), but these potentially beneficial effects of prazosin are not unequivocal (Raskind et al 2018; Hendrickson et al 2021). Conversely, yohimbine, an α_2_-adrenoceptor antagonist, has been used as an experimental tool to induce anxiety in animals (Pellow et al. 1985) and humans (Charney et al. 1984).

##### Study characteristics for the noradrenergic system

Six different noradrenergic compounds from four drug classes were tested in nine experiments (Table 3). Two of these compounds were clinically active anxiolytics, representing two different drug classes: the α_2_-adrenoceptor agonist clonidine and the β_1,2_-adrenoceptor antagonist propranolol. With regard to experimental drugs, four experimental compounds were tested covering two drug classes. Drug classes included the α_1_-adrenoreceptor antagonists (one experiment, one drug) and the α_2_-adrenoreceptor antagonists (six experiments, three drugs), which are generally considered anxiogenic drugs.

For six of the nine experiments, no or incomplete data were reported for fear potentiation as well as for the non-cued baseline startle response. Effect size estimates could therefore only be calculated for the α_2_-adrenoreceptor antagonists yohimbine and atipamezole (Fig. 6b). Given the paucity of data, effects on fear potentiation could not readily be interpreted. Regarding the non-cued baseline startle response, it is interesting to note that these anxiogenic-like drugs enhanced the non-cued baseline startle response in all three experiments (Fig. 6b).

Data were insufficient to conduct a meta-analysis to determine the significance of this effect.

Given the wide range of anxiety symptoms that are affected by different noradrenergic drug classes in humans, it is unfortunate that noradrenergic drugs have not been studied more extensively in the fear-potentiated startle paradigm. This may have provided a better understanding of the role of the different noradrenergic receptors in the expression of conditioned fear. Furthermore, such studies could have been useful to disentangle which aspects of anxiety are reflected in the fear-potentiated and non-cued baseline startle response.

#### Dopaminergic system

Several lines of evidence suggest that the dopamine system modulates aversive states (de Vita et al. 2021). Exposure to acute stressors for instance alters dopaminergic transmission (Goldstein et al. 1996). As such, it has been suggested that dopaminergic drugs may alter the expression of the conditioned fear response (de Oliveira et al. 2006).

##### Study characteristics for the dopaminergic system

So far, six different dopaminergic drugs from five different drug classes have been tested in nine experiments (Table 3). Drug classes included the D_1_ receptor agonists, the D_1_ receptor antagonists, and the D_2_ receptor agonists which were all tested once in relatively large samples (*n* = 15–20). The D_2_ receptor antagonists (two drugs) were tested in four experiments, whereas the dopamine releasers were tested twice.

The available data on dopaminergic drug classes were insufficient to conduct a meta-analysis and do not allow firm conclusions on the effects of dopaminergic drugs in the fear-potentiated startle test. Yet, it is interesting to note that the individual effect size estimates suggest that haloperidol may reduce fear potentiation (Fig. 6c). Haloperidol, however, also reduced the non-cued baseline startle response in these two experiments and the observed variance for both potentiated and baseline startle response was considerable. The findings with haloperidol were not mirrored in the effects of sulpiride, the other D_2_ receptor antagonist tested (Fig. 6c). Given the paucity of data, further research is necessary to substantiate a possible role for D_2_ receptor ligands in the modulation of the fear-potentiated startle response.

#### Opioid system

The opioid receptor system is best known for its role in the regulation of pain and reward. Yet, μ and δ receptor agonists have also been shown to exert anxiolytic-like effects in animal tests for anxiety (Anand and Montgomery 2018; Nagase and Saitoh 2020) and μ receptors may modulate threat processing and fear conditioning in humans (Meier et al. 2021).

##### Study characteristics for the opioid system

So far, four different drugs from three different drug classes all involving the μ receptor have been tested in ten experiments. Drug classes included the μ receptor agonists (four experiments, two drugs), the μ receptor partial agonists (two experiments, one drug), and the μ receptor antagonists (four experiments, one drug). Results from these experiments as reported in the articles are summarized in Table 3.

For six experiments, no data or incomplete data on fear potentiation was reported. Drug effects on the non-cued baseline startle response after treatment were not fully reported for three experiments*.* The forest plot is therefore limited to four experiments.

As shown in Fig. 6d, both morphine and buprenorphine had moderate to large beneficial effects on fear potentiation, but confidence intervals were rather wide. In these experiments, the (partial) μ receptor agonists had no effect on the non-cued baseline startle response. Although the μ receptor agonist fentanyl showed a different profile, that is, no effect on fear potentiation and a small but significant reduction in the non-cued baseline startle response, together the data suggest that μ receptor agonism may reduce fear potentiation without major effects on the non-cued baseline startle response.

According to the reported effects, naloxone did not alter fear potentiation and baseline startle response in any of the four experiments (Table 3). This is in line with the effect size estimates (Fig. 6). This absence of effect of the μ receptor antagonist may suggest that endogenous endorphins do not, or only slightly modulate the fear-potentiated startle response in animals.

#### Neuropeptide systems

Neuropeptides play an important role in the regulation of emotional behavior. As such, the development of drugs that target neuropeptide systems could offer a valuable addition to existing anxiolytics that target the GABA-ergic or serotonergic system (Sartori and Singewald 2019).

##### Study characteristics for the neuropeptide systems

**Fig. 7:**
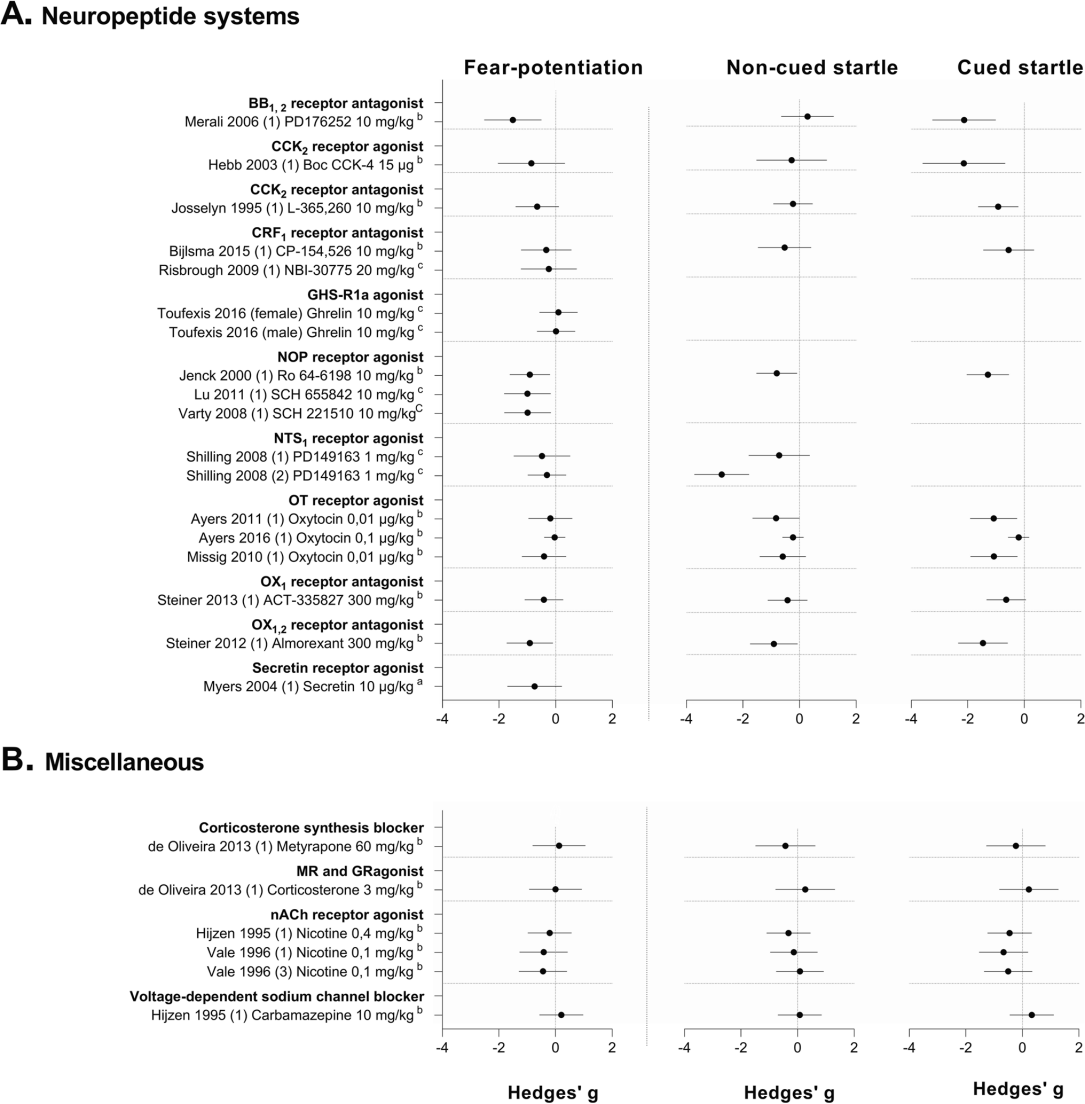
Forest plots of the effects of neuropeptidergic drugs (**A**) and drug classes that were not further categorized (**B**) on fear potentiation (left), the non-cued startle (middle), and the cued startle response (right). Data are shown as Hedge’s *g* and 95% confidence intervals. Fear potentiation represents ^a^ = reported difference score, ^b^ = calculated difference score, ^c^ = % fear potentiation

In the fear-potentiated startle test, 14 experimental drugs from 11 different drug classes have been tested in 19 separate experiments. Together, the drug classes targeted nine different neuropeptide systems (see Fig. 7a, Table 3). Most drug classes were studied for their anxiolytic potential and were tested in only one or two experiments (Table 3). These classes included the bombesin (BB)_1,2_ receptor antagonists, the orexin (OX)_1_ receptor antagonists, the OX_1,2_ receptor antagonists, and the neurotensin (NTS)_1_ receptor agonists. The oxytocin (OT) receptor agonists (three experiments, one drug), the corticotropin-releasing factor (CRF)_1_ receptor antagonists (three experiments, two drugs), and nociception opioid peptide (NOP) receptor agonists (three experiments, three drugs) were studied a bit more extensively. Two drug classes, the cholecystokinin (CCK)_2_ receptor agonists (one experiment, one drug) and the GHS-R1a agonists (two experiments, one drug), were tested for their presumed anxiogenic-like properties.

Drug effects on non-cued baseline startle response were not fully reported for seven experiments. For one experiment, the number of animals was not reported.

There was insufficient data to pool data and conduct a meta-analysis for any of the drug classes. Visual inspection of the forest plots (Fig. 7a) showed that the effect size estimates for drugs tested for anxiolytic potential reduced fear potentiation and the non-cued baseline startle response relative to vehicle treatment, but effects were small and non-significant, except for the BB1,2 antagonist, the OX1,2 receptor antagonist, and the NOP receptor agonists. In fact, the three different NOP receptor agonists that were tested all significantly reduced fear potentiation, with a small to moderate effect size, reasonable group sizes (*n* = 12–16), and moderate variance*.* Ro 64–6198 also reduced the non-cued startle response (Fig. 7a). For the other NOP agonists, the authors indicated that these drugs had no effect on the non-cued baseline startle response (Table 3), but actual data on the non-cued startle response were not reported. Therefore, the observed reduction in percentage fear potentiation following treatment with the NOP receptor agonists cannot readily be interpreted as a potentially anxiolytic drug effect.

Systemic administration of the presumed anxiogenic compounds Boc CCK-4 and ghrelin had no effect on fear potentiation. Boc CCK-4 also had no effect on the non-cued startle response (Fig. 7a), whereas the effects of ghrelin on the non-cued baseline startle response were not reported. Data were insufficient to determine the profile of these anxiogenic drugs in the fear-potentiated startle test.

#### Miscellaneous

##### Study characteristics

Four different neurotransmitter systems were grouped under miscellaneous: the cholinergic system, the endocannabinoid system, the glucocorticoid system, and “other.” In total, six different drugs from six different drug classes were tested in eight experiments. Drug classes included the nACh receptor agonists, cannabinoid (CB)1 receptor antagonists, cannabinoid reuptake inhibitors, glucocorticoid receptor agonists, corticosterone synthesis inhibitors, and voltage-dependent sodium channel blockers. Further details of these experiments are summarized in Table 3.

None of these drugs had a reported effect on fear potentiation or the non-cued baseline startle response. There was not enough data to conduct a meta-analysis. As shown in Fig. 7b, drug effects found in individual experiments were generally small and non-significant. The apparent absence of effect of nicotine on the non-cued baseline startle response is in line with findings in humans (Braun et al. 2012).

## Discussion

With this systematic review, we aimed to determine the effect of different drug classes on the fear-potentiated startle and non-cued baseline response in the fear-potentiated startle test. Data synthesis showed that the fear-potentiated startle response is sensitive to acute treatment with the clinically used anxiolytics benzodiazepines and the 5-HT_1A_ receptor partial agonist buspirones. 5-HT_1A_ receptor antagonists and mGluR2,3 agonists also reduced the fear-potentiated startle response. The non-cued baseline startle response appeared sensitive to GABA_A_-ergic drugs but not so much to drugs that alter serotonergic and glutamatergic signaling. Results further suggested that alterations in noradrenergic, dopaminergic, and opioid signaling may modulate the fear-potentiated startle response. This suggestion should, however, be taken with caution given the limited amount of data available.

### Pharmacological interventions and predictive validity of the fear-potentiated startle paradigm

#### Clinically active drugs

Meta-analysis showed that the clinically active anxiolytics benzodiazepines and buspirone reduced fear potentiation. Based on individual studies, we concluded that acute treatment with SSRIs had no anxiolytic effect in the fear-potentiated startle test. In fact*,* the small but consistently positive effect sizes observed in the individual single-dose studies included in this review may reflect anxiogenic-like effects of SSRIs. Interestingly, comparable effects were found in a study with healthy human subjects. Acute treatment with citalopram exacerbated the fear-potentiated startle response as well as the non-cued startle response (Grillon et al. 2007). In patients with an anxiety disorder, acute treatment may also increase symptoms of anxiety (Grillon et al. 2007), and repeated dosing of SSRIs is necessary to obtain anxiolytic effects (Baldwin et al. 2014). Given these findings, the sensitivity of the fear-potentiated startle test may be considered high. On the other hand, the effects of a single dose of SSRI may depend on the type of anxiety studied. A meta-analysis of drug effects in the separation-induced vocalization test showed that acute treatment with SSRIs reduced anxiety to a similar extent as benzodiazepines did in non-stressed guinea pigs (Groenink et al. 2015). This might indicate that acute treatment with SSRIs may suppress unconditioned defensive behavior, but not conditioned fear in animals. From this perspective, the fear-potentiated startle test may be less suited as a global screen for anxiolytic drug properties.

#### Experimental drugs

About half of the experimental drugs that were tested reduced the fear-potentiated startle response. This suggests that the test may exert a reasonable level of specificity. Among the potentially anxiolytic drugs that reduced fear potentiation were several drug classes that target 5-HT_1A_ receptors. A meta-analysis confirmed that 5-HT_1A_ receptor agonists and antagonists reduced fear potentiation, and individual studies indicated anxiolytic effects of 5-HT_1A_ partial agonists and the biased agonist F13714, which preferentially acts on somatodendritic autoreceptors (Newman-Tancredi et al. 2022). As extensively discussed by Zhao and co-workers, 5-HT_1A_ receptor agonists probably reduce fear potentiation via actions on presynaptic 5-HT_1A_ receptors, although a role for postsynaptic 5-HT_1A_ heteroreceptor in this anxiolytic effect cannot be excluded (Zhao et al. 2019). From a translational perspective, it is interesting to note that, although it was a small pilot study, the selective 5-HT_1A_ receptor agonist flesinoxan had no effect in patients with panic disorder and increased anxiety at high doses (van Vliet et al. 1996). Results of this pilot study would qualify flesinoxan as a false positive in the rat fear-potentiated startle test.

Regarding the glutamatergic system, GlyR partial agonists, mGluR2,3 agonists, and mGluR5 antagonists were among the drug classes for which predominantly anxiolytic effects were found in the individual studies. A meta-analysis confirmed the anxiolytic effect of mGluR2,3 agonists as a drug class. It is interesting that the effects reported for LY354740 closely resembled those observed in healthy humans. Just as in the rat fear-potentiated startle test, this mGluR2,3 agonist reduced fear potentiation and had no significant effect on the non-cued startle response (Grillon et al. 2003). In addition, LY544344, a mGluR2,3 agonist and prodrug of LY354740, had beneficial effects in patients with a generalized anxiety disorder (Dunayevich et al. 2007), whereas LY354740 was not efficacious in patients with panic disorder (Bergink and Westenberg 2005). Future studies may clarify to what extent these glutamatergic drug classes may add to the treatment of patients with anxiety disorders.

All three NOP agonists had moderate effects on fear potentiation. Although data were too limited for a meta-analysis, the observed reduction in fear potentiation may be interesting to pursue. The endogenous ligand of the NOP receptor, nociceptin/orphanin FQ, is expressed in brain areas that are involved in conditioned fear, such as the prefrontal cortex and basolateral amygdala, but also in brain stem nuclei (Ubaldi et al. 2021). As such, the N/OFQ-NOP system may modulate anxiety by altering serotonergic and noradrenergic neurotransmission in these areas. As recently reviewed by Ubaldi and co-workers, clinical development has unfortunately been hampered by the limited bioavailability of NOP receptor agonists (Ubaldi et al. 2021).

In 32% of the experiments, the experimental drugs under study did not alter fear potentiation. Interestingly, most drugs that targeted neuropeptide systems were among the drugs that had no effect on fear potentiation. Except for NOP receptor agonists, neuropeptidergic drugs generally had small, non-significant effects. This may suggest that neuropeptides play a limited role in the regulation of cued fear. Accordingly, for both CRF and oxytocin, there is considerable evidence that these neuropeptides may reduce sustained, general anxiety but not cued fear in rats (Ayers et al. 2016; Bijlsma et al. 2011; de Jongh et al. 2003; Missig et al. 2010; Walker et al. 2009).

Most drugs that were tested as a “negative control condition,” e.g., carbamazepine, nicotine, and naloxone, were also among the drugs that had no effect. The fact that the test successfully differentiates between presumed active and non-active compounds adds to the predictive validity of the fear-potentiated startle test. In addition, the finding that the psychostimulants d-amphetamine and nicotine did not alter fear potentiation suggests that an increase in general activity does not impact the outcome measure and adds to the robustness of the test.

#### Anxiogenic drugs

Visual inspection of the forest plots did not reveal a distinct profile for anxiogenic drugs in this test. In some experiments, effect size estimates were positive, indicative of an increase in fear potentiation. In most experiments, however, the anxiogenic drugs had no or only small, non-significant negative effects on fear potentiation. The direction of effect of the anxiogenic drug classes seemed unrelated to the neurotransmitter system target. Although data were too limited to conduct a meta-analysis, the current data suggest that anxiogenic-like drug effects are not reliably expressed as an increase in fear potentiation. This finding may be related to the observation that anxiogenics may exert strong effects on the non-cued baseline startle response, which may distort the drug effects on fear potentiation (Bijlsma et al. 2010; Risbrough and Geyer 2005).

### Contextual anxiety and drug effects on the non-cued baseline startle response

A secondary aim of this review was to determine the effects of drugs on the non-cued baseline startle response. During fear conditioning, animals will not only acquire the cue-shock contingency but also learn to associate the foot shock with the experimental context. Upon re-exposure to the context during testing, this may induce sustained, contextual anxiety (Groenink et al. 2008).

So far, methodological studies have been inconclusive as to whether drug-induced changes in the non-cued startle response just reflect non-specific drug effects, such as motor effects and sedation (Joordens et al. 1997) or may also reflect changes in contextual anxiety (Guscott et al. 2000; Missig et al. 2010; Zhao et al. 2018b). Although most evidence supports the notion of a contextual anxiety component in the non-cued baseline startle response, methodological studies have been focused on GABA_A_-ergic drugs, limiting the generalization of these findings. The current meta-analysis showed that benzodiazepines significantly reduced the non-cued baseline startle response, as did 5-HT_1A_ receptor antagonists. Buspirone and 5-HT_1A_ receptor agonists, on the other hand, did not alter the non-cued baseline startle response. This indicates that clinically used anxiolytics may show different profiles in the fear-potentiated startle test; anxiolytics could specifically reduce the fear-potentiated startle or reduce both the cued and the non-cued startle response. Whether such a differentiation results from an actual difference in the types of anxiety these drug classes may alleviate or is more generally related to their mechanism of action, e.g., a general CNS dampening of the central nervous system, cannot be concluded from the limited data available. Results from the individual experiments in which SSRIs and some anxiogenic drugs tended to enhance the non-cued baseline startle and partial GABA_A_ receptor agonists and mGluR5 receptor antagonists seemed to reduce this response may suggest that the non-cued startle response indeed reflects elements of contextual anxiety that is sensitive to modulation by different drug classes. This would also be in line with effects of drugs that selectively altered the non-cued baseline startle response, in the absence of effects on the fear-potentiated startle response, such as oxytocin.

It would be worthwhile to extend the work on the non-cued baseline startle response with other drug classes using specifically dedicated control conditions. Building on the studies already conducted for GABA_A_-ergic drugs and oxytocin, control conditions could include non-shocked animals and randomly trained animals (Ayers et al. 2016; Davis 1979; Hijzen and Slangen 1989; Joordens et al. 1997). The use of these control conditions may help to differentiate between non-specific drug effects, effects on general arousal, contextual and background anxiety as well as cued conditioned fear. These studies could provide insight into the types of anxiety that are represented by the cued and non-cued startle response and indicate how these responses may be used to predict the clinical potential of experimental drugs.

### Methodological characteristics

Ever since the introduction of the fear-potentiated startle paradigm (Brown et al. 1951), researchers have investigated how experimental factors affect the acquisition and the expression of conditioned fear in this test. With this systematic review, we intended to take the results of these individual studies a step further. By synthesizing the outcome of all available pharmacological studies with information on the experimental set-up that was applied, we aimed to identify methodological factors that are associated with larger effect sizes in pharmacological studies. Our data synthesis, however, showed that evidence was too limited to draw conclusions regarding species differences, sex differences, and the light–dark cycle. Below, we therefore only discuss those factors for which subgroup analyses could be performed. A discussion on the other methodological factors is provided in Supplementary File 8.

#### Animal characteristics

Meta-analysis suggested that the effects of benzodiazepines in the fear-potentiated startle test are dependent on the strain that is tested. The reduction in fear potentiation was stronger in Sprague–Dawley rats than in Wistar rats. Such a strain difference was not apparent for the effect of benzodiazepines on the non-cued baseline startle response. These findings are in line with an elegant study performed by Steiner and co-workers. They demonstrated strain differences in the fear-potentiated startle response, non-cued baseline as well as pretraining baseline startle responses (Steiner et al. 2011). They further showed that these strain differences were dissimilar for the three outcome measures. This finding might explain why in the meta-analysis strain differences were observed for fear potentiation but not for the non-cued startle response.

Given that the prevalence of anxiety disorders is higher in females than in males (Bandelow and Michaelis 2015), it is remarkable that female subjects were tested in only 3 of the 68 articles. Studies that assessed the effect of sex on the fear-potentiated startle response perse showed conflicting results with either no sex differences (Zhao et al. 2018a) or stronger fear-potentiation in female rats (de Jongh et al. 2005; Toufexis et al. 2016). In the two articles that compared drug effects in male and female subjects, no sex differences were found in the fear-potentiated startle response to diazepam, chlordiazepoxide (Zhao et al. 2018a), or ghrelin (Toufexis et al. 2016). From a translational perspective, it would be important to include female subjects in future studies.

#### Characteristics of the acquisition training procedure

Foot-shock intensity has been shown to affect fear conditioning in rats, following an inverted U-shape intensity–effect curve (Davis and Astrachan 1978; Leaton and Borszcz 1985). This may explain why in most articles a moderate shock intensity (0.4–0.6 mA) was used. According to the subgroup analyses we conducted, the effects of benzodiazepines on fear potentiation and on the non-cued baseline response did not seem associated with foot shock intensity or the total number of cue-shock pairings used. This finding may be specific to benzodiazepines and not necessarily generalize to other drug classes. In fact, individual studies suggest that drug effects may be dependent on the foot-shock intensity used. Nevins and Anthony (1994) showed that 5-HT_3_ receptor antagonists only reduced fear potentiation in rats that were trained with 0.25-mA foot shocks, but not 0.5 mA. In comparison, diazepam and buspirone did reduce the fear-potentiated startle under both training protocols in that same study (Nevins and Anthony 1994). Likewise, nicotine and d-amphetamine reduced fear potentiation in rats conditioned with 0.25-mA foot shocks (Vale and Green 1996), whereas no effects were found after training with 0.6 mA (Hijzen et al. 1995). Considering the predictive validity of the test, together these findings may suggest that using moderate-intensity foot shocks (0.50–0.70 mA) during fear conditioning would contribute to the specificity of the test.

#### Characteristics of the test procedure

It has been hypothesized that the actions of anxiolytic drugs may depend on the intensity of the startling noise used. Anxiolytic drugs would be less effective in reducing fear potentiation when high noise intensities are used (Davis et al. 1988). Subgroup analysis, however, did not suggest that the effects of benzodiazepines on either fear potentiation or the non-cued startle baseline response were dependent on startling noise intensity.

Although we cannot provide substantiated recommendations for refinement or optimization of the experimental setup, the systematic map does provide information on frequently used setups of the fear-potentiated startle test (Supplementary File 2, 4). From this information, the following general pattern can be deduced. Drugs are almost always tested in group-housed, adult Sprague–Dawley or Wistar rats. A commonly used set-up for the acquisition training consists of a foot shock with an intensity of 0.6 mA and a duration of 500 ms, which is delivered during the last 500 ms of a 3700-ms cue-light presentation. These cue-shock pairings, generally 20 in total, are presented with a variable time interval and divided over two training sessions. The time between the training and test sessions is typically 24 h. Protocols for the test session generally start with a 5-min acclimation period which is followed by presentation of habituation trials. The two trial types that are used to elicit the cued and non-cued startle responses are usually presented 30 times each, with different noise intensities and in a pseudo-random order. However, the fact that some setups and characteristics are frequently used does not necessary mean that these parameters are also the best once to use in a protocol. For example, in contrast to common practice, we would recommend to test both male and female rats, and to house animals under a reversed day–night cycle, because these factors may contribute to the translational value of the test (see Supplementary File 8). Of note, the characteristics described here have predominantly been used with males as test subjects. Also, since results of individual research papers suggest that different acquisition training procedures may induce qualitatively different anxiety states (Davis & Astrachan 1978; Nevins & Anthony 1994) and drug efficacy may be dependent on anxiety state (Nevins & Anthony 1994), it seems important to demonstrate that the protocol that is being used is established within the laboratory as sensitive to standard anxiolytics.

### Key study quality indicators, study design, and quality of reporting

In the majority of articles, measures were taken to reduce possible selection bias, which may occur due to differences in the baseline startle response. For this, several approaches were used including randomization, matching, and the use of a balanced Latin-square design. Subgroup analysis did not indicate that the method used affected the effect size. Yet, given the known individual differences in (fear-potentiated) startle response, a stratified random sampling design may be the preferred method for the fear-potentiated startle test. In a stratified random sampling design, animals are divided into groups based on their (potentiated) startle response, and then an appropriate number of animals from each group is randomly allocated to the experimental conditions. Such a design would control for confounding parameters, such as differences in startle response, while maintaining random allocation, thereby reducing the risk of bias.

A balanced within-subject design (balanced Latin-square) in which each animal receives each dose of the assigned drug in a counterbalanced order could provide an alternative approach to control for individual differences in startle response and reduce the number of animals needed for an experiment.

We found that hardly any article reported if the experiments were blinded or if a sample size calculation had been performed. Therefore, the study quality was generally considered low. Unfortunately, this poor level of reporting on study quality indicators is often found in preclinical animal research (Macleod et al. 2015). Quality of reporting may improve, however, now that several initiatives have raised awareness among researchers (e.g., Bespalov et al. 2021) and reporting guidelines have been adopted by many journals (Kilkenny et al. 2010; Percie du Sert et al. 2020). The fact that the one study that did report on both blinding and power calculations is a recent article (Zhao et al. 2018b) may support this notion.

## Limitations

We limited this systematic review to single-dose studies in healthy animals, a set-up that is often used to screen compounds for anxiolytic properties. An additional systematic review may help to determine the predictive validity of the test in case of chronic drug treatment and the added value of testing animals that have been exposed to stressors or other procedures to raise their basal level of anxiety. Given our aim to formulate recommendations on how to optimize the testing procedures, we further limited this review to studies that used cue light and foot shock during acquisition training, and acoustic stimuli to elicit the startle response. Future studies should indicate to what extend the use of different stimulus modalities may affect drug effects in the fear-potentiated startle test.

For the meta-analysis of drug effects on fear potentiation, we chose to only pool data that had been corrected for drug effects on the non-cued baseline startle response. We, therefore, calculated the difference score between fear-potentiated startle and non-cued baseline response if the differences score had not been reported in the original articles. Since the absolute startle values were reported at the group level, we could not use within-subject difference scores to compute the calculated mean difference score. This may have affected the effect size estimates, although this was not apparent from the sensitivity analysis.

Due to the limited data for the different drug classes, the subgroup analysis on the impact of methodological characteristics could only be performed for benzodiazepines. It is unclear if the findings would generalize to drugs with a different mechanism of action. In addition, subgroups were generally small and not all moderators may have been independent of each other, although we treated them as such. This may have affected the outcome of the subgroup analyses.

Since the overall quality of the included studies was poor, drug efficacy may have been overestimated both in the individual studies and in the meta-analyses. Finally, the effects of benzodiazepines on fear potentiation may also have been overestimated because publication bias likely occurred.

## Concluding remarks and recommendations

The fear-potentiated startle test seems to have moderate to high predictive validity if used as a test to detect anxiolytic properties after single drug administration. Additional studies are, however, necessary to further corroborate the sensitivity and specificity of the test. Given the translational value of the fear-potentiated startle test, it is unsatisfactory to see that the use of this test has declined in the past 10 years. It is, however, unclear how this reduced use relates to the use of other animal tests for anxiety. The observed decline could also reflect a more general reduction in preclinical psychopharmacological research in the field of anxiety.

We performed this systematic review and meta-analysis to provide a complete and objective overview of the effect of different drug classes on the fear-potentiated startle test. A meta-analysis has added value over comparing separate experiments with and without statistically significant effects. Pooling the data of individual experiments enhances the statistical power and may therefore detect effects that were not found in separate smaller experiments. Vice versa, a large significant drug effect in an imprecise experiment may prove insignificant upon pooling with data from more precise, larger experiments. Proper reporting of experimental outcome data is crucial to be able to include experiments in a meta-analysis. In the current review, this information was not reported for 42 experiments, approximately 20% of the included articles. This limited the number of drug classes for which a meta-analysis could be conducted. Faster progress may be achieved in this research field if we as a community would not only properly report but also share data of future studies via repositories.

We extracted many methodological details from the articles, which we documented in an openly accessible systematic map. Methods varied considerably between studies, which probably contributed to the high levels of heterogeneity in the meta-analyses, and also limited the power of the subgroup analyses.

The analyses did not allow for recommendations on how to optimize or refine the experimental procedure. Yet, although we do appreciate that certain settings are specific for particular labs for the test to deliver robust results, it may aid the field to align the main characteristics of the training and test procedures between laboratories in future studies. The systematic map could prove helpful for that.

### Supplementary Information

Below is the link to the electronic supplementary material.

Supplementary file1 (DOCX 47 KB)

Supplementary file2 (XLSX 267 KB)

Supplementary file3 (DOCX 35 KB)

Supplementary file4 (XLSX 30 KB)

Supplementary file5 (DOCX 85 KB)

Supplementary file6 (DOCX 101 KB)

Supplementary file7 (DOCX 111 KB)

Supplementary file8 (DOCX 112 KB)

## Data Availability

https://osf.io/m4nw3/?view_only=5c6c0ebbad014b88b42126fdd42a3af6
