# Supplementary material for: Pharmacological modulation of conditioned fear in the fear-potentiated startle test: a systematic review and meta-analysis of animal studies
Source: Psychopharmacology (Berl). 2023 Jan 18;240(11):2361–401. doi: 10.1007/s00213-022-06307-1 (PMC10593622; doi:10.1007/s00213-022-06307-1)
Supplement: Supplementary file 1 — Supplementary file1 (DOCX 47 KB) [file 213_2022_6307_MOESM1_ESM.docx]

**Pharmacological modulation of conditioned fear in the fear-potentiated startle test: a systematic review and meta-analysis of animal studies**

Psychopharmacology

Lucianne Groenink, P Monika Verdouw, Yulong Zhao, Freija ter Heegde, Kimberley E Wever, Elisabeth Y Bijlsma

Corresponding author: Lucianne Groenink, l.groenink@uu.nl

**Supplementary File 1** Analysis of the year of publication of articles reporting on the fear-potentiated startle protocol as screen for acute, systemic drug effects

**Supplementary File 1** Analysis of the year of publication of articles reporting on the fear-potentiated startle protocol as screen for acute, systemic drug effects


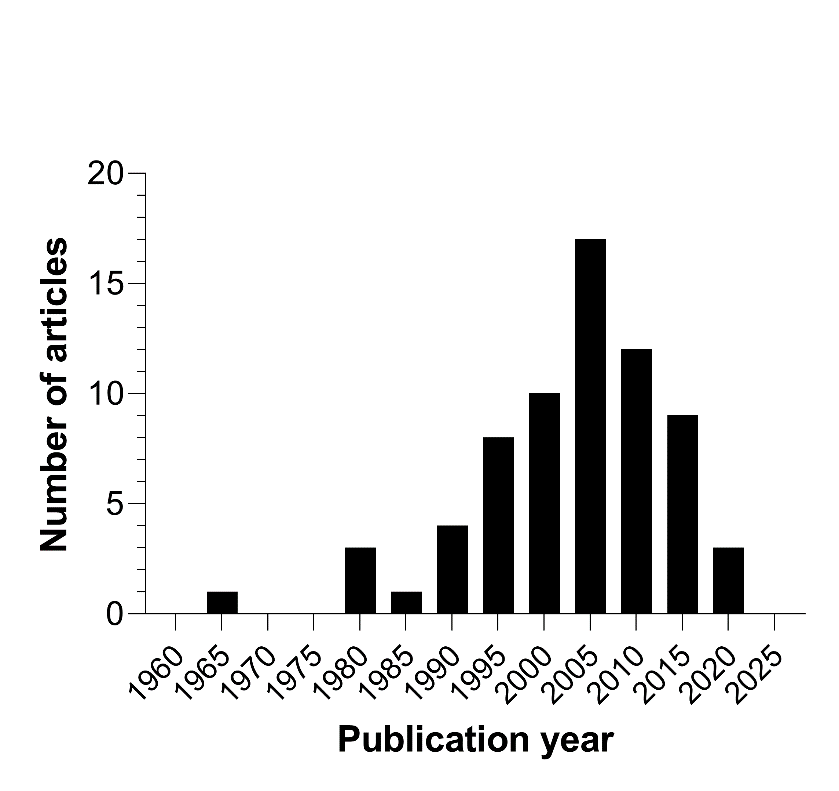


An analysis of the year of publication of the included articles showed that the first paper that studied acute, systemic drug effects in the fear-potentiated startle test was published in 1965. The number of publications peaked between 2000 and 2010 and has gradually declined
