# Supplementary material for: Pharmacological modulation of conditioned fear in the fear-potentiated startle test: a systematic review and meta-analysis of animal studies
Source: Psychopharmacology (Berl). 2023 Jan 18;240(11):2361–401. doi: 10.1007/s00213-022-06307-1 (PMC10593622; doi:10.1007/s00213-022-06307-1)
Supplement: Supplementary file 3 — Supplementary file3 (DOCX 35 KB) [file 213_2022_6307_MOESM3_ESM.docx]

**Pharmacological modulation of conditioned fear in the fear-potentiated startle test: a systematic review and meta-analysis of animal studies**

Psychopharmacology

Lucianne Groenink, P Monika Verdouw, Yulong Zhao, Freija ter Heegde, Kimberley E Wever, Elisabeth Y Bijlsma

Corresponding author: Lucianne Groenink, l.groenink@uu.nl

**Supplementary File 3** Study characteristics of animal and housing conditions

**Supplementary File 3** Study characteristics of animal and housing conditions

| Article | Species | Strain | Sex | Age (wks) | Bodyweight (g) | Housing | Time of testing  (period light-dark cycle) |
| --- | --- | --- | --- | --- | --- | --- | --- |
| Anthony and Nevins, 1993 | Rats | SDR | m | NR | 250-450 | 1 to 2 | passive |
| Atack et al., 2006 | Rats | SDR | m | NR | 250-300 | NR | NR |
| Atack et al., 2011 | Rats | SDR | m | NR | 260-310 | NR | NR |
| Ayers et al., 2011 | Rats | SDR | m | NR | 225-250 | group | passive |
| Ayers et al., 2016 | Rats | SDR | m | NR | 225-250 | group | passive |
| Bijlsma et al., 2010 | Rats | Wi | m | NR | 300-350 | group | passive |
| Bijlsma et al., 2015 | Rats | Wi (wt) | m | NR | NR | group | passive |
| Bill et al., 1992 | Rats | HL | m | NR | 250-285 | group | active |
| Brodkin et al., 2002 | Rats | Wi | m | adult | 225-300 | group | passive |
| Busse et al., 2004 | Rats | Wi | m | adult | 225-300 | group | passive |
| Caracache et al., 2011 | Rats | NR | m | NR | NR | NR | NR |
| Cassella and Davis, 1985 | Rats | SDR | m | NR | 300-400 | group | NR |
| Chen et al., 1997 | Rats | SDR | NR | NR | NR | NR | NR |
| Chhatwal et al., 2005 | Rats | SDR | m | NR | 350-450 | group | passive |
| Chi, 1965 | Rats | SDR | m | 13-17 | NR | NR | NR |
| Collado et al., 2002 | Rats | NR | NR | NR | NR | NR | NR |
| Collado et al., 2004 | Rats | SDR | m | NR | 225-274 | group | NR |
| Cosford et al., 2003 | Rats | NR | NR | NR | NR | NR | NR |
| Davis, 1979a | Rats | SDR | m | NR | 250-300 | group | passive |
| Davis, 1979b | Rats | SDR | m | NR | 250-300 | group | NR |
| Davis et al., 1979c | Rats | SDR | m | NR | 250-300 | group | NR |
| Davis 1988a | Rats | SDR | m | NR | 300-400 | group | NR |
| de Oliveira et al., 2006 | Rats | Wi | m | NR | 220-280 | group | passive |
| de Oliveira et al., 2013 | Rats | Wi | m | NR | 270-290 | group | NR |
| Gacsályi et al., 2017 | Rats | SDR | m | NR | 190-270 | NR | passive |
| Glover and Davis, 2008 | Rats | SDR | m | NR | 350-450 | group | passive |
| Guscott et al., 2000 | Rats | SDR | m | NR | 250-350 | group | NR |
| Hebb et al., 2003 | Mice | CD-1 | m | 12 | NR | single | passive |
| Helton et al., 1998 | Rats | LE | m | NR | 180-400 | NR | NR |
| Hijzen and Slangen, 1989 | Rats | Wi | m | NR | 200-220 | group | NR |
| Hijzen et al., 1995 | Rats | Wi | m | NR | 175-200 | group | passive |
| Jenck et al., 2000 | Rats | Wi | m | NR | 260-280 | NR | passive |
| Johnson et al., 2003 | Rats | NR | NR | NR | NR | NR | NR |
| Johnson et al., 2005 | Rats | SDR | m | NR | 325-400 | single | NR |
| Joordens et al., 1996 | Rats | Wi | m | NR | 150-200 | group | passive |
| Joordens et al., 1997 | Rats | Wi | m | NR | 175-200 | group | passive |
| Joordens et al., 1998 | Rats | Wi | m | NR | 175-200 | group | passive |
| Josselyn et al., 1995 | Rats | Wi | m | NR | 275-325 | single | passive |
| Li et al., 2015 | Rats | Wi | m | NR | 300-350 | group | active |
| Lu et al., 2011 | Rats | Wi | m | NR | 200-300 | group | passive |
| Mansbach and Geyer, 1988 | Rats | SDR | m | NR | NR | group | active |
| Martin et al., 2002 | Rats | RORO | m | adult | 260 | NR | NR |
| Merali et al., 2006 | Rats | NR | m | NR | 300-375 | NR | NR |
| Missig et al., 2010 | Rats | SDR | m | NR | 225-250 | group | passive |
| Muthuraju, 2014 | Rats | Wi | M | NR | 250-300 | Single | passive |
| Myers et al., 2004 | Rats | SDR | m | NR | 350-450 | group | NR |
| Nevins and Anthony, 1994 | Rats | SDR | m | NR | 250-450 | 1 to 2 | passive |
| Pietraszek et al., 2005 | Rats | SDR | m | adult | 240-280 | group | passive |
| Risbrough et al., 2003 | Mice | DBA/1J | m | 6-8 | NR | group | active |
| Risbrough and Geyer, 2005 | Mice | DBA/1J; 129Sv | m;f | 6-8 | NR | group | active |
| Risbrough et al., 2009 | Mice | C57BL/ 6J(WT) | m/f | NR | NR | group | active |
| Roppe et al., 2004a | Rats | NR | NR | NR | NR | NR | NR |
| Roppe et al., 2004b | Rats | Wi | NR | NR | NR | NR | NR |
| Rorick-Kehn et al., 2007 | Rats | SDR | m | NR | 200-350 | group | NR |
| Schulz et al., 2001 | Rats | SDR | m | NR | 210-300 | group | NR |
| Shilling and Feifel, 2008 | Rats | SDR | m | NR | 275-375 | group | passive |
| Steiner et al., 2011 | Rats | F344 | m | NR | 250-300 | group | passive |
| Steiner et al., 2012 | Rats | F344 | m | 8-10 | 240-260 | group | passive |
| Steiner et al., 2013 | Rats | F344 | m | adult | NR | group | passive |
| Tizzano et al., 2002 | Rats | SDR | m | NR | 250-400 | single | NR |
| Toufexis et al., 2016 | Rats | SDR | m,f | 13-17 | NR | NR | passive |
| Vale and Green, 1996 | Rats | LH | m | NR | 275-350 | group | active |
| Varty et al., 2008 | Rats | Wi | m | NR | 200-500 | group | passive |
| Walker et al., 2002, | Rats | SDR | m | adult | 300-400 | group | active |
| Zhang and Li, 2016 | Rats | SDR | m | 8-13 | NR | group | passive |
| Zhao et al.,2018a | Rats | Wi | m | 6 | NR | group | passive |
| Zhao et al., 2018b | Rats | Wi | m,f | 6 | NR | group | active |
| Zhao et al., 2019 | Rats | Wi | m | 6 | NR | group | passive |

F344= Fischer 344; f = female; LE = Long Evans; LH = Lister Hooded; m = male; NR = not reported; SDR = Sprague-Dawley rat; Wi = Wistars. ^a, b, c^ denote separate experiments within the same article
