# Supplementary material for: Pharmacological modulation of conditioned fear in the fear-potentiated startle test: a systematic review and meta-analysis of animal studies
Source: Psychopharmacology (Berl). 2023 Jan 18;240(11):2361–401. doi: 10.1007/s00213-022-06307-1 (PMC10593622; doi:10.1007/s00213-022-06307-1)
Supplement: Supplementary file 5 — Supplementary file5 (DOCX 85 KB) [file 213_2022_6307_MOESM5_ESM.docx]

**Pharmacological modulation of conditioned fear in the fear-potentiated startle test: a systematic review and meta-analysis of animal studies**

Psychopharmacology

Lucianne Groenink, P Monika Verdouw, Yulong Zhao, Freija ter Heegde, Kimberley E Wever, Elisabeth Y Bijlsma

Corresponding author: Lucianne Groenink, l.groenink@uu.nl

**Supplementary file 5** Overview of methodological characteristics used in each article sorted by general characteristics (5.1), acquisition training (5.2), and test session (5.3, 5.4)

**Table 5.1** Study characteristics of startle equipment and pretraining sessions

| *Nr* | *Paper* | *Year* | *Startle equipment* | *Calibration equipment* | *Background noise intensity (dB)* | *Pre-training startle exposure (#trials*intensity (dB), interval (s)(# days))* |
| --- | --- | --- | --- | --- | --- | --- |
| 1 | Anthony and Evins | 1993 | SR-LAB | yes | 80 | NR |
| 2 | Atack et al., | 2006 | SR-LAB | NR | NR | 10*100, NR |
| 3 | Atack et al., | 2011 | NR | NR | NR | NR |
| 4 | Ayers et al., | 2011 | SR-LAB | NR | 65 | 30*95 / 105 / 115, 15 |
| 5 | Ayers et al., | 2016 | SR-LAB | NR | 65 | 30*95 / 105 / 115, 15 (3) |
| 6 | Bijlsma et al., | 2010 | SR-LAB | yes | 55 | NR |
| 7 | Bijlsma et al., | 2015 | SR-LAB | NR | 70 | NR |
| 8 | Bill et al., | 1992 | Campden | NR | 75 | NA |
| 9 | Brodkin et al., | 2002 | SR-LAB | NR | NR | NR |
| 10 | Busse et al., | 2004 | SR-LAB | NR | NR | NR |
| 11 | Caracache et al., | 2011 | NR | NR | NR | NR |
| 12 | Cassella and Davis | 1985 | Custom | NR | 55 | 30*85 / 100 / 115, NR |
| 13 | Chen et al., | 1997 | NR | NR | 68 | 20*120, NR |
| 14 | Chhatwal et al., | 2005 | Custom | NR | 60 | Pre-exposure to box only, NA (2) |
| 15 | Chi | 1965 | Custom | NR | NR | No |
| 16 | Collado et al., | 2002 | SR-LAB | NR | NR | 30*120, NR |
| 17 | Collado et al., | 2004 | NR | NR | NR | NR |
| 18 | Cosford et al., | 2003 | NR | NR | NR | NR |
| 19 | Davis | 1979a | Custom | NR | 46 | 10* NR, NR |
| 20 | Davis | 1979b | Custom | NR | 46 | 10*110, 20 |
| 21 | Davis et al., | 1979c | Custom | NR | 45 | 10*110, 20 |
| 22 | Davis 1988a | 1988 | Custom | NR | 55 | 30*95 / 100 / 105, NR |
| 23 | de Oliveira et al., | 2006 | MED | yes | 55 | 30*100, NR (2) |
| 24 | de Oliveira et al., | 2013 | Custom | NR | 55 | 30*100, NR (2) |
| 25 | Gacsályi et al., | 2017 | NR | NR | 55 | NR |
| 26 | Glover and Davis | 2008 | Custom | NR | 60 | 30*95, 30 |
| 27 | Guscott et al., | 2000 | SR-LAB | NR | 55 | 10*100, 30 |
| 28 | Hebb et al., | 2003 | Custom | NR | 50 | 10*100, 30-35 (3) |
| 29 | Helton et al., | 1998 | SR-LAB | NR | NR | No |
| 30 | Hijzen and Slangen | 1989 | Custom | NR | 51 | 20*105 / 110, NR |
| 31 | Hijzen et al., | 1995 | Custom | NR | 55 | 30*95 / 105, NR |
| 32 | Jenck et al., | 2000 | NR | NR | NR | NR |
| 33 | Johnson et al., | 2003 | NR | NR | NR | NR |
| 34 | Johnson et al., | 2005 | NR | NR | NR | 30*120, NR |
| 35 | Joordens et al., | 1996 | Custom / SR-LAB | NR | 65 | 30*105 / 115, NR |
| 36 | Joordens et al., | 1997 | SR-LAB | NR | 78 | No |
| 37 | Joordens et al., | 1998 | Custom | NR | 65 | 30*105 / 115, 30 |
| 38 | Josselyn et al., | 1995 | Custom | NR | 70 | 15*119, 60 |
| 39 | Li et al., | 2015 | SR-LAB | NR | NR | 30*100, 30 (2) |
| 40 | Lu et al., | 2011 | SR-LAB | NR | NR | NR |
| 41 | Mansbach and Geyer | 1988 | SR-LAB | yes | NR | No |
|  |  |  |  |  |  |  |
| 42 | Martin et al., | 2002 | SR-LAB | yes | 68 | No |
| 43 | Merali et al., | 2006 | MED | NR | 50 | No |
| 44 | Missig et al., | 2010 | SR-LAB | NR | 65 | 10*95 / 105 / 115, 15 |
| 45 | Muthuraju et al., | 2014 | MED | NR | 55 | 30*100, 30 (2) |
| 46 | Myers et al., | 2004 | Custom | NR | 60 | 30*95,30 |
| 47 | Nevins and Anthony | 1994 | SR-LAB | yes | 80 | No |
| 48 | Pietraszek et al., | 2005 | Custom | NR | 64 | 30*95 / 100 / 105, NR |
| 49 | Risbrough et al., | 2003 | SR-LAB | yes | 65 | 10*100, NR |
| 50 | Risbrough and Geyer | 2005 | SR-LAB | yes | 65 | 10*100, NR |
| 51 | Risbrough et al., | 2009 | SR-LAB | yes | 65 | 24*100 / 105, NR |
| 52 | Roppe et al., | 2004a | NR | NR | NR | NR |
| 53 | Roppe et al., | 2004b | NR | NR | NR | NR |
| 54 | Rorick-Kehn et al., | 2007 | SR-LAB | NR | 70 | No |
| 55 | Schulz et al., | 2001 | Custom | NR | 52 | No |
| 56 | Shilling and Feifel | 2008 | SR-LAB | NR | 65 | 30*95, NR |
| 57 | Steiner et al., | 2011 | SR-LAB | yes | 62 | No |
| 58 | Steiner et al., | 2012 | SR-LAB | yes | 62 | No |
| 59 | Steiner et al., | 2013 | SR-LAB | yes | 62 | NR |
| 60 | Tizzano et al., | 2002 | SR-LAB | NR | NR | 30*120, NR |
| 61 | Toufexis et al., | 2016 | Custom | NR | 60 | 30*90 / 95, 30 (2) |
| 62 | Vale and Green | 1996 | Custom | NR | 60 | No |
| 63 | Varty et al., | 2008 | SR-LAB | NR | NR | No |
| 64 | Walker et al., | 2002 | Custom | NR | 60 | 30*95, NR |
| 65 | Zhang and Li | 2016 | KS | NR | 70 | 18*105, 25-35 (2) |
| 66 | Zhao et al., | 2018a | SR-LAB | NR | 70 | 10*95 / 100 / 110, 30 |
| 67 | Zhao et al., | 2018b | SR-LAB | yes | 70 | 10*95 / 100 / 110, 30 |
| 68 | Zhao et al., | 2019 | SR-LAB | yes | 70 | 10*95 / 100 / 110, 30 |

NA = not applicable; NR = not reported

**Table 5.2** Study characteristics of acquisition training sessions

| *Nr* | *Paper* | *Year* | *Shock intensity (mA)* | *Shock duration (ms)* | *Cue light duration (ms)* | *Timing of shock (ms)* | *n Cue-shock pairings per session* | *n Training sessions (n/day; n days)* | *Calculated total number of pairings* | *Interval of pairing (fixed/variable)* | *Interval of pairing (s): mean (range)* |
| --- | --- | --- | --- | --- | --- | --- | --- | --- | --- | --- | --- |
| 1 | Anthony and Nevins | 1993 | 0,5 | 500 | 3000 | 2500 | 20 | 1;2 | 40 | fixed | 20 |
| 2 | Atack et al. | 2006 | 0,6 | 500 | 3700 | 3200 | 10 | 1;2 | 20 | NR | NR |
| 3 | Atack et al. | 2011 | NR | NR | NR | NR | NR | NR | na | NR | NR |
| 4 | Ayers et al. | 2011 | 0,6 | 500 | 3000 | 2500 | 5 | 1;1 | 5 | variable | NR  (60-180) |
| 5 | Ayers et al. | 2016 | 0,6 | 500 | 3000 | 2500 | 10 | 1;1 | 10 | variable | NR  (60-180) |
| 6 | Bijlsma et al. | 2010 | 0,6 | 500 | 3700 | 3200 | 10 | 1;2 | 20 | variable | 240  (180-300) |
| 7 | Bijlsma et al. | 2015 | 0,6 | 500 | 3700 | 3200 | 10 | 1;2 | 20 | variable | 240  (180-300) |
| 8 | Bill et al. | 1992 | NR | NR | NR | NR | NR | 1;2 | na | NR | NR |
| 9 | Brodkin et al. | 2002 | 0,6 | 500 | 10000 | 10000 | 30 | 1;2 | 60 | fixed | 60 |
| 10 | Busse et al. | 2004 | 0,6 | 500 | 10000 | 9500 | 30 | 1;2 | 60 | fixed | 60 |
| 11 | Caracache et al. | 2011 | 0,5 | 500 | 5000 | NR | 20 | 1;2 | 40 | variable | 120  (60-150 |
| 12 | Cassella and Davis | 1985 | 0,6 | 500 | 3700 | 3200 | 10 | 1;2 | 20 | variable | 240  (180-300) |
| 13 | Chen et al. | 1997 | 1,2 | 500 | 3200 | 2700 | 20 | 1;1 | 20 | fixed | 120 |
| 14 | Chhatwal et al. | 2005 | 0,4 | 500 | 3700 | 3200 | 10 | 1;2 | 20 | fixed | 216 |
| 15 | Chi | 1965 | 158 V | 200 | 7500 | 7500 | 32 | 1;1 | 32 | variable | 150  (NR) |
| 16 | Collado et al. | 2002 | 0,5 | 500 | 5500 | 5000 | 10 | 1;1 | 10 | NR | NR |
| 17 | Collado et al. | 2004 | NR | NR | NR | NR | NR | NR | na | NR | NR |
| 18 | Cosford et al. | 2003 | NR | NR | NR | NR | NR | NR | na | NR | NR |
| 19 | Davis | 1979a | 0,4 | 500 | 3000 | 2500 | 45 | 1;2 | 90 | variable | 60  (45-75) |
| 20 | Davis | 1979b | 0,25 | 500 | 1000 | 500 | 45 | 1;2 | 90 | variable | 60  (45-75) |
| 21 | Davis et al. | 1979c | 0,25 | 500 | 1000 | 500 | 45 | 1;2 | 90 | variable | 60  (45-75) |
| 22 | Davis 1988a | 1988 | 0,6 | 500 | 3700 | 3200 | 10 | 1;2 | 20 | variable | 240  (180-300) |
| 23 | de Oliveira et al. | 2006 | 0,6 | 1000 | 4000 | 3000 | 10 | 1;2 | 20 | variable | NR  (60-180) |
| 24 | de Oliveira et al. | 2013 | 0,6 | 1000 | 4000 | 3000 | 10 | 1;1 | 10 | variable | NR  (60-180) |
| 25 | Gacsályi et al. | 2014 | 1,0 | 500 | 3700 | 3200 | 10 | 1;2 | 20 | variable | 180  (NR-NR) |
| 26 | Glover and Davis | 2008 | 0,4 | 500 | 3700 | 3200 | 10 | 1;1 | 10 | fixed | 240 |
| 27 | Guscott et al. | 2000 | 0,6 | 500 | 3700 | 3200 | 10 | 1;2 | 20 | fixed | 60 |
| 28 | Hebb et al. | 2003 | 0,6 | 500 | 30000 | 29500 | 10 | 1;2 | 20 | variable | 210  (150-270) |
| 29 | Helton et al. | 1998 | 1 | 500 | 5500 | 5000 | 10 | 1;2 | 20 | fixed | 10 |
| 30 | Hijzen and Slangen | 1989 | 0,4 | 500 | 1000 | 500 | 45 | 1;2 | 90 | variable | 60  (45-75) |
| 31 | Hijzen et al. | 1995 | 0,6 | 500 | 3700 | 3200 | 10 | 1;2 | 20 | variable | 240  (180-300) |
| 32 | Jenck et al. | 2000 | NR | NR | NR | NR | NR | NR | na | NR | NR |
| 33 | Johnson et al. | 2003 | NR | NR | NR | NR | NR | NR | na | NR | NR |
| 34 | Johnson et al. | 2005 | 0,6 | 500 | 5000 | 4500 | 10 | 1;1 | 10 | fixed | 10 |
| 35 | Joordens et al. | 1996 | 0,6 | 500 | 3700 | 3200 | 10 | 1;2 | 20 | variable | 240  (180-300) |
| 36 | Joordens et al. | 1997 | 1 | 500 | 3000 | 3000 | 15 | 1;2 | 30 | fixed | 180 |
| 37 | Joordens et al. | 1998 | 0,6 | 500 | 3700 | 3200 | 10 | 1;2 | 20 | variable | 240  (180-300) |
| 38 | Josselyn et al. | 1995 | 0,5 | 500 | 3000 | 2500 | 10 | 1;3 | 30 | variable | 300  (240-360) |
| 39 | Li et al. | 2015 | 0,4 | 500 | 3700 | 3200 | 10 | 1;2 | 20 | variable | 180  (130-230) |
| 40 | Lu et al. | 2011 | 0,4 | 500 | 3500 | 3000 | 20 | 1;2 | 40 | variable | 90  (NR) |
| 41 | Mansbach and Geyer | 1988 | 1,0 / 1,25 | 500 | 2500 / 3200 | 2000 / 2700 | 10 or 15 | 1;2 | na | variable | NR  (120–240) |
| 42 | Martin et al. | 2002 | 0,25 | 500 | 3700 | 3200 | 15 | 1;2 | 30 | variable | 30  (20-40) |
| 43 | Merali et al. | 2006 | 0,65 | 500 | 3700 | 3200 | 20 | 1;2 | 40 | variable | 60  (45-75) |
| 44 | Missig et al. | 2010 | 0,6 | 500 | 3000 | 2500 | 5 | 1;1 | 5 | variable | NR  (60-180) |
| 45 | Muthuraju et al. | 2014 | 0,6 | 1000 | 4000 | 3000 | 10 | 1;1 | 10 | variable | NR  (60-180) |
| 46 | Myers et al. | 2004 | 0,4 | 500 | 3700 | 3200 | 10 | 1;2 | 20 | variable | 240  (180-300) |
| 47 | Nevins and Anthony | 1994 | 0,25 | 500 | 3000 | 2500 | 10 | 1;2 | 20 | fixed | 20 |
|  | Nevins and Anthony | 1994 | 0,5 | 500 | 3000 | 2500 | 15 | 1;1 | 15 | fixed | 20 |
| 48 | Pietraszek et al. | 2005 | 0,6 | 500 | 3700 | 3200 | 10 | 1;2 | 20 | variable | 60  (30-90) |
| 49 | Risbrough et al. | 2003 | 0,14 | 500 | 30000 | 29500 | 20 | 1;1 | 20 | fixed | 120 |
| 50 | Risbrough and Geyer | 2005 | 0,14 | 500 | 30000 | 29500 | 20 | 1;4 | 80 | fixed | 120 |
| 51 | Risbrough et al. | 2009 | 0,4 | 250 | 30000 | 30000 | 10 | 1;2 | 20 | variable | 180  (130-230) |
| 52 | Roppe et al. | 2004a | NR | NR | NR | NR | NR | NR | na | NR | NR |
| 53 | Roppe et al. | 2004b | NR | NR | NR | NR | NR | NR | na | NR | NR |
| 54 | Rorick-Kehn et al. | 2007 | 1 | 500 | 5000 | 4500 | 10 | 1;2 | 20 | fixed | 10 |
| 55 | Schulz et al. | 2001 | 0,6 | 500 | 3700 | 3200 | 10 | 1;1 | 10 | variable | 120  (90-150) |
| 56 | Shilling and Feifel | 2008 | 0,6 | 500 | 3700 | 3200 | 10 | 1;2 | 20 | variable | 120  (60-180) |
| 57 | Steiner et al. | 2011 | 0,4 | 500 | 3700 | 3200 | 15 | 2;2 | 30 | variable | 30  (NR) |
| 58 | Steiner et al. | 2012 | 0,4 | 500 | 3700 | 3200 | 15 | 2;2 | 30 | variable | 30  (NR) |
| 59 | Steiner et al. | 2013 | 0,4 | 500 | 3700 | 3200 | 15 | 2;2 | 30 | variable | 30  (NR) |
| 60 | Tizzano et al. | 2002 | 0,5 | 500 | 5500 | 5000 | 10 | 1;1 | 10 | NR | NR |
| 61 | Toufexis et al. | 2016 | 0,4 | 500 | 3700 | 3200 | 10 | 1;2 | 20 | fixed | 180 |
| 62 | Vale and Green | 1996 | 0,25 | 500 | 1000 | 500 | 17 | 1;3 | 51 | fixed | 10 |
| 63 | Varty et al. | 2008 | 0,4 | 500 | 3500 | 3000 | 20 | 1;2 | 40 | variable | 90  (NR) |
| 64 | Walker et al. | 2002 | 0,4 | 500 | 3700 | 3200 | 10 | 1;1 | 10 | variable | 240  (180-300) |
| 65 | Zhang and Li | 2016 | 0,6 | 500 | 3700 | 3200 | 10 | 1;1 | 10 | variable | 240  (180-300) |
| 66 | Zhao et al. | 2018a | 0,6 | 500 | 3700 | 3200 | 10 | 1;1 | 10 | variable | 240  (180-300) |
| 67 | Zhao et al. | 2018b | 0,6 | 500 | 3700 | 3200 | 10 | 1;1 | 10 | variable | 240  (180-300) |
| 68 | Zhao et al. | 2019 | 0,6 | 500 | 3700 | 3200 | 10 | 1;1 | 10 | variable | 240  (180-300) |

Mansbach & Geyer: Not specified which characteristics were used for each drug. Alterations in procedure did not affect baseline startle according to authors

**Table 5.3** Study characteristics of test sessions (I)

| *Nr* | *Paper* | *Year* | *Interval between last training and testing (hr)* | *Test in other box than training* | *Acclimatization (min)* | *Startle habituation (N*dB,ISI)* | *N non-cued/ cued trials* | *Noise intensity (dB)* | *Noise duration (ms)* |
| --- | --- | --- | --- | --- | --- | --- | --- | --- | --- |
| 1 | Anthony and Nevins | 1993 | 72 | NR | 10 | 30*110, 20 | 20/ 20 | 110 | NR |
| 2 | Atack et al. | 2006 | 24 | no | 5 | 10*100, 30 | 5/ 5 | 95 | 50 |
| 3 | Atack et al. | 2011 | NR | NR | NR | NR | NR | 100 | NR |
| 4 | Ayers et al. | 2011 | 24 | no | 5 | 10*95 / 105 / 115, 15 | 30/ 30 | 95 / 105 / 115 | 50 |
| 5 | Ayers et al. | 2016 | 24 | no | 5 | 10*95 / 105 / 115, 15 | 30/ 30 | 95 / 105 / 115 | 50 |
| 6 | Bijlsma et al. | 2010 | 24 | no | 5 | 10*105, 30 | 15/ 15 | 90 / 95 / 105 | 50 |
| 7 | Bijlsma et al. | 2015 | 24 | no | 5 | 10*105, 30 | 20/ 20 | 100 / 105 | 50 |
| 8 | Bill et al. | 1992 | 24 | no | 7 | No | 20/ 20 | 96 | 1000 |
| 9 | Brodkin et al. | 2002 | 24 | NR | NR | No | 21/ 21 | 95 | 20 |
| 10 | Busse et al. | 2004 | 24 | NR | NR | No | 21/ 21 | 95 | 20 |
| 11 | Caracache et al. | 2011 | 24 | NR | NR | 12*102, 30 | 12/ 12 | 102 | 50 |
| 12 | Cassella and Davis | 1985 | 24 | yes | 5 | No | 30/ 30 | 85 / 100 / 115 | 50 |
| 13 | Chen et al. | 1997 | 72 | NR | NR | 10*108, NR | NR | 108 | 40 |
| 14 | Chhatwal et al. | 2005 | 120 | no | NR | No | 15/ 15 | 95 | 50 |
| 15 | Chi | 1965 | 18 | no | 5 | 10*122, NR | 0/ 32 ^C^ | 122 | 100 |
| 16 | Collado et al. | 2002 | 24 | no | 5 | NR | NR | NR | NR |
| 17 | Collado et al. | 2004 | NR | NR | NR | NR | NR | NR | NR |
| 18 | Cosford et al. | 2003 | NR | NR | NR | NR | NR | NR | NR |
| 19 | Davis | 1979a | 24 | yes | 5 | No | 40/ 40 | 105 / 110 | 90 |
| 20 | Davis | 1979b | 24 | yes | 5 | No | 40/ 40 | 110 | 90 |
| 21 | Davis et al. | 1979c | 24 | yes | 5 | No | 40/ 40 | 105 / 110 | 90 |
| 22 | Davis 1988a | 1988 | 72-96 | yes | 5 | 10*95, 30 | 30/ 30 | 90 / 95 / 105 | 50 |
| 23 | de Oliveira et al. | 2006 | NR | no | 5 | No | 30/ 30 | 100 | 50 |
| 24 | de Oliveira et al. | 2013 | 24 | yes | 5 | No | 30/ 30 | 100 | 50 |
| 25 | Gacsályi et al. | 2017 | 48 | no | 5 | 10*95, 20-40 | 10/ 10 | 95 | NR |
| 26 | Glover and Davis | 2008 | 24-48 | no | 5 | 30*95, 30 | 15/ 15 | 95 | 50 |
| 27 | Guscott et al. | 2000 | 24 | no | 5 | 10*100, 30 | 10/ 10 | 95 or 100 | 50 |
| 28 | Hebb et al. | 2003 | 24 | no | 5 | No | 0/ 10 ^C^ | 100 | 10 |
| 29 | Helton et al. | 1998 | 48 | no | NR | 10*110, NR | 10/ 10 | 110 | 50 |
| 30 | Hijzen and Slangen | 1989 | 24 | no | NR | No | 20/ 20 | 105 / 110 | NR |
| 31 | Hijzen et al. | 1995 | 24 | no | 5 | 10*100, 30 | 15/ 15 | 95 / 100 / 110 | 50 |
| 32 | Jenck et al. | 2000 | NR | NR | NR | NR*95, NR | 15/ 15 | 90 / 95 / 105 | 50 |
| 33 | Johnson et al. | 2003 | NR | NR | NR | No | NR | NR | NR |
| 34 | Johnson et al. | 2005 | 24 | NR | 5 | 10*120 ^B^ | 10/ 10 | 120 | 50 |
| 35 | Joordens et al. | 1996 | 24 | no | 5 | 10*110, 30 | 15/ 15 | 105 / 110 / 115 | 50 |
| 36 | Joordens et al. | 1997 | 24 | no | 5 | 10*118, 30 | 0/ 50 ^C^ | 118 | 25 |
| 37 | Joordens et al. | 1998 | 24 | no | 5 | 10*110, 30 | 15/ 15 | 105 / 110 / 115 | 50 |
| 38 | Josselyn et al. | 1995 | 96-144 | no | NR | 5*119, 60 | 20/ 20 | 119 | NR |
| 39 | Li et al. | 2015 | 24 | no | 5 | No | 30/ 30 | 100 | 40 |
| 40 | Lu et al. | 2011 | 24 | no | NR | 5*105, 25 | 10/ 10 | 105 | NR |
| 41 | Mansbach and Geyer | 1988 | 24-48 | no | 5 | 1, 6 or 11*97 / 108 / 118, NR | NR | 97 / 108 / 118 | NR |
| 42 | Martin et al. | 2002 | 24 | yes | 5 | 10*95, 30 | 15/ 15 | 90 / 95 / 105 | 50 |
| 43 | Merali et al. | 2006 | NR | no | 5 | 20*110, NR | 5/ 5 | 110 | NR |
| 44 | Missig et al. | 2010 | 96 | no | 5 | 10*95, 15 | 30/ 30 | 95 / 105 / 115 | NR |
| 45 | Muthuraju et al. | 2014 | 24 | yes | 5 | No | 30/ 30 | 100 | 50 |
| 46 | Myers et al. | 2004 | 24 | no | 5 | 30*95, 30 | 9/ 9 | 95 / 100 / 105 | 50 |
| 47 | Nevins and Anthony | 1994 | 72 | no | 10 | No | 20/ 20 | 110 | NR |
| 48 | Pietraszek et al. | 2005 | 24 | no | 5 | 6*95 / 100 / 105, 30 | 15/ 15 | 95 / 100 / 105 | 50 |
| 49 | Risbrough et al. | 2003 | 24 | no | 5 | No | 12/ 12 | 100 | 40 |
| 50 | Risbrough and Geyer | 2005 | 24 | no | 5 | No | 12/ 12 | 100 | 40 |
| 51 | Risbrough et al. | 2009 | 24 | no | NR | 8*100 / 105, 60 | 8/ 8 | 100 / 105 | 40 |
| 52 | Roppe et al. | 2004a | NR | NR | NR | NR | NR | NR | NR |
| 53 | Roppe et al. | 2004b | NR | NR | NR | NR | NR | NR | NR |
| 54 | Rorick-Kehn et al. | 2007 | 48 | NR | NR | 10*110, NR | 10/ 10 | 110 | 50 |
| 55 | Schulz et al. | 2001 | 48 | no | 5 | 10*100, 30 | 10/ 10 | 100 | 20 |
| 56 | Shilling and Feifel | 2008 | 24 | no | 5 | 10*95, NR | 20/ 20 | 95 | 40 |
| 57 | Steiner et al. | 2011 | 24 | no | 5 | NR | 30/ 30 | 90 / 100 / 110 | 10 |
| 58 | Steiner et al. | 2012 | 24 | no | 5 | No | 30/ 30 | 90 / 100 /110 | 10 |
| 59 | Steiner et al. | 2013 | 24 | no | 5 | No | 30/ 30 | 90 / 100 / 110 | 10 |
| 60 | Tizzano et al. | 2002 | 24 | no | NR | 10*120, NR | 20/ 20 | 120 | NR |
| 61 | Toufexis et al. | 2016 | 48 | no | 5 | 15*90, 30 & 15*95, 30 ^A^ | 30/ 30 | 90 & 95 ^A^ | 50 |
| 62 | Vale and Green | 1996 | >3 | yes | 3 | No | 20/ 20 | 100 | 500 |
| 63 | Varty et al. | 2008 | 48 | NR | NR | No | 10/ 10 | 105 | NR |
| 64 | Walker et al. | 2002 | 24 | no | 5 | 30*95, 30 | 10/ 10 | NR | 50 |
| 65 | Zhang and Li | 2016 | 72 | no | 5 | 4*105, 25-35 | 10/ 10 | 105 | 40 |
| 66 | Zhao et al. | 2018a | 24 | yes & no | 5 | 10*110, 30 | 30/ 30 | 95 / 100 / 110 | 50 |
| 67 | Zhao et al. | 2018b | 24 | no | 5 | 10*110, 30 | 30/ 30 | 95 / 100 / 110 | 50 |
| 68 | Zhao et al. | 2019 | 24 | no | 5 | 10*110, 30 | 30/ 30 | 95 / 100 / 110 | 50 |

**Table 5.4** Study characteristics of test sessions (II)

| *Nr* | *Paper* | *Year* | *Interval time (fixed/variable)* | *Interval time: mean (range) (s)* | *Trial order (balanced, random, pseudo-random)* | *Cue light duration (ms)* | *Startle probe relative to cue light (ms)* | *Startle measure duration (ms)* |
| --- | --- | --- | --- | --- | --- | --- | --- | --- |
| 1 | Anthony and Nevins | 1993 | Fixed | 20 | Pseudo-Random | 3000 | 2500 | NR |
| 2 | Atack et al. | 2006 | Fixed | 30 | Random | 3200 | 3200 | 100 |
| 3 | Atack et al. | 2011 | NR | NR | NR | NR | NR | NR |
| 4 | Ayers et al. | 2011 | Fixed | 15 | Pseudo-Random | 3000 | NR | NR |
| 5 | Ayers et al. | 2016 | Fixed | 15 | Pseudo-Random | 3000 | 2950 | 100 |
| 6 | Bijlsma et al. | 2010 | Fixed | 30 | Pseudo-Random | 3250 | 3200 | 65 |
| 7 | Bijlsma et al. | 2015 | Fixed | 30 | NR | 3250 | 3200 | 65 |
| 8 | Bill et al. | 1992 | Fixed | 10 | Pseudo-Random | NR | NR | NR |
| 9 | Brodkin et al. | 2002 | Fixed | 30 | Pseudo-Random | 10000 | NR | NR |
| 10 | Busse et al. | 2004 | Fixed | 30 | Pseudo-Random | 10000 | NR | NR |
| 11 | Caracache et al. | 2011 | Fixed | 30 | NR | NR | NR | NR |
| 12 | Cassella and Davis | 1985 | Fixed | 30 | Pseudo-Random | 3700 | 3200 | 200 |
| 13 | Chen et al. | 1997 | NR | NR | NR | 3200 | 3200 | NR |
| 14 | Chhatwal et al. | 2005 | NR | NR | NR | 3700 | NR | 2 |
| 15 | Chi | 1965 | Variable | 150 | Pseudo-Random | 7500 | 7500 | 1000 |
| 16 | Collado et al. | 2002 | NR | NR | NR | NR | NR | NR |
| 17 | Collado et al. | 2004 | NR | NR | NR | NR | NR | NR |
| 18 | Cosford et al. | 2003 | NR | NR | NR | NR | NR | NR |
| 19 | Davis | 1979a | Variable | 30 (25-35) | Pseudo-Random | 2500 | 2500 | 200 |
| 20 | Davis | 1979b | Variable | 30 (25-35) | Pseudo-Random | 1000 | 500 | 200 |
| 21 | Davis et al. | 1979c | Variable | 30 (25-35) | Pseudo-Random | 1000 | 500 | 200 |
| 22 | Davis 1988a | 1988 | Fixed | 30 | Pseudo-Random | 3700 | 3200 | 200 |
| 23 | de Oliveira et al. | 2006 | Fixed | 30 | NR | 4000 | 3000 | 100 |
| 24 | de Oliveira et al. | 2013 | Fixed | 30 | Random | 4000 | 3000 | 100 |
| 25 | Gacsályi et al. | 2017 | NR | NR | Random | NR | NR | NR |
| 26 | Glover and Davis | 2008 | Fixed | 30 | Pseudo-Random | 3700 | 3200 | 300 |
| 27 | Guscott et al. | 2000 | Fixed | 30 | Random | 3200 | 3200 | 100 |
| 28 | Hebb et al. | 2003 | Variable | 30 or 35 | NA | 30000 | 29850 | 150 |
| 29 | Helton et al. | 1998 | Fixed | 8 | Alternating | 5000 | NR | NR |
| 30 | Hijzen and Slangen | 1989 | Variable | 25 (20-30) | Pseudo-Random | 1000 | 500 | 200 |
| 31 | Hijzen et al. | 1995 | Fixed | 30 | Pseudo-Random | 3700 | 3200 | 200 |
| 32 | Jenck et al. | 2000 | NR | NR | Random | NR | NR | NR |
| 33 | Johnson et al. | 2003 | NR | NR | NR | NR | NR | NR |
| 34 | Johnson et al. | 2005 | NR | NR | Random | NR | NR | NR |
| 35 | Joordens et al. | 1996 | Fixed | 30 | Pseudo-Random | 3200 | 3200 | 200 |
| 36 | Joordens et al. | 1997 | Fixed | 30 | NA | 3000 | 3000 | 200 |
| 37 | Joordens et al. | 1998 | Fixed | 30 | Pseudo-Random | 3700 | 3200 | 200 |
| 38 | Josselyn et al. | 1995 | NR | NR | Pseudo-Random | 3000 | 2500 | 100 |
| 39 | Li et al. | 2015 | Variable | 60 (30-90) | Pseudo-Random | NR | NR | NR |
| 40 | Lu et al. | 2011 | Variable | 25 (20-30) | Pseudo-Random | NR | NR | NR |
| 41 | Mansbach and Geyer | 1988 | NR | NR | NR | 2500 / 3200 | 2500 / 3200 | 100 |
| 42 | Martin et al. | 2002 | Variable | 30 (20-40) | NR | 3700 | NR | NR |
| 43 | Merali et al. | 2006 | NR | NR | NR | 3700 | 3200 | NR |
| 44 | Missig et al. | 2010 | Fixed | 15 | Pseudo-Random | 3000 | NR | NR |
| 45 | Muthuraja et al. | 2014 | Fixed | 30 | Random | 4000 | 3950 | 100 |
| 46 | Myers et al. | 2004 | Fixed | 30 | Pseudo-Random | 3700 | 3200 | 200 |
| 47 | Nevins and Anthony | 1994 | Fixed | 20 | Pseudo-Random | 3000 | NR | 150 |
| 48 | Pietraszek et al. | 2005 | Variable | 30 (15-45) | NR | 3700 | NR | NR |
| 49 | Risbrough et al. | 2003 | Fixed | 120 | Pseudo-Random | 30000 | NR | 200 |
| 50 | Risbrough and Geyer | 2005 | Fixed | 120 | Pseudo-Random | 30000 | 29500 | 200 |
| 51 | Risbrough et al. | 2009 | Variable | 60 (30-90) | Pseudo-Random | 30000 | NR | 65 |
| 52 | Roppe et al. | 2004a | NR | NR | NR | NR | NR | NR |
| 53 | Roppe et al. | 2004b | NR | NR | NR | NR | NR | NR |
| 54 | Rorick-Kehn et al. | 2007 | Fixed | 8 | Alternating | 5000 | NR | NR |
| 55 | Schulz et al. | 2001 | Fixed | 30 | Pseudo-random | 3700 | 3200 | 80 |
| 56 | Shilling and Feifel | 2008 | Fixed | 30 | Blocks | NR | 3200 | NR |
| 57 | Steiner et al. | 2011 | variable | 30 (25-35) | Pseudo-random | 3200 | 3200 | 100 |
| 58 | Steiner et al. | 2012 | Variable | 30 (25-35) | Pseudo-Random | 3 | 3200 | 100 |
| 59 | Steiner et al. | 2013 | Variable | 30 (25-35) | Pseudo-Random | 3700 | 3200 | 100 |
| 60 | Tizzano et al. | 2002 | NR | NR | Random | NR | NR | NR |
| 61 | Toufexis | 2016 | Fixed | 30 | Pseudo-Random | 3250 | 3200 | 200 |
| 62 | Vale and Green | 1996 | Fixed | 10 | Random | 1000 | 500 | 250 |
| 63 | Varty et al. | 2008 | Variable | 25 (20-30) | NR | 3500 | NR | NR |
| 64 | Walker et al. | 2002 | Fixed | 30 | Pseudo-Random | 3700 | 3200 | 200 |
| 65 | Zhang and Li | 2016 | Variable | 30 (25-35) | Random | 3240 | 3200 | 100 |
| 66 | Zhao et al. | 2018a | Fixed | 30 | Pseudo-Random | 3250 | 3200 | NR |
| 67 | Zhao et al. | 2018b | Fixed | 30 | Pseudo-Random | 3250 | 3200 | 65 |
| 68 | Zhao et al. | 2019 | Fixed | 30 | Pseudo-Random | 3250 | 3200 | 65 |

**Table 4.5** Study characteristics of test sessions (III)

| *Nr* | *Paper* | *Year* | *Background intensity (dB)* | *Noise intensity (dB)* | *Lowest intensity (calculated dB)* | *Highest intensity (calculated dB)* | *Intensity range (dB)* | *Noise duration (ms)* |
| --- | --- | --- | --- | --- | --- | --- | --- | --- |
| 1 | Anthony and Nevins | 1993 | 80 | 110 | 30 | 30 | 0 | NR |
| 2 | Atack et al. | 2006 | NR | 95 | NR | NR | NR | 50 |
| 3 | Atack et al. | 2011 | NR | 100 | NR | NR | NR | NR |
| 4 | Ayers et al. | 2011 | 65 | 95 / 105 / 115 | 30 | 50 | 20 | 50 |
| 5 | Ayers et al. | 2016 | 65 | 95 / 105 / 115 | 30 | 50 | 20 | 50 |
| 6 | Bijlsma et al. | 2010 | 55 | 90 / 95 / 105 | 35 | 50 | 15 | 50 |
| 7 | Bijlsma et al. | 2015 | 70 | 100 / 105 | 30 | 35 | 5 | 50 |
| 8 | Bill et al. | 1992 | 75 | 96 | 21 | 21 | 0 | 1000 |
| 9 | Brodkin et al. | 2002 | NR | 95 | NR | NR | NR | 20 |
| 10 | Busse et al. | 2004 | NR | 95 | NR | NR | NR | 20 |
| 11 | Caracache et al. | 2011 | NR | 102 | NR | NR | NR | 50 |
| 12 | Cassella and Davis | 1985 | 55 | 85 / 100 / 115 | 30 | 60 | 30 | 50 |
| 13 | Chen et al. | 1997 | 68 | 108 | 40 | 40 | 0 | 40 |
| 14 | Chhatwal et al. | 2005 | 60 | 95 | 35 | 35 | 0 | 50 |
| 15 | Chi | 1965 | NR | 122 | NR | NR | NR | 100 |
| 16 | Collado et al. | 2002 | NR | NR | NR | NR | NR | NR |
| 17 | Collado et al. | 2004 | NR | NR | NR | NR | NR | NR |
| 18 | Cosford et al. | 2003 | NR | NR | NR | NR | NR | NR |
| 19 | Davis | 1979a | 46 | 105 / 110 | 59 | 64 | 5 | 90 |
| 20 | Davis | 1979b | 46 | 110 | 64 | 64 | 0 | 90 |
| 21 | Davis et al. | 1979c | 45 | 105 / 110 | 60 | 65 | 5 | 90 |
| 22 | Davis 1988a | 1988 | 55 | 90 / 95 / 105 | 35 | 50 | 15 | 50 |
| 23 | de Oliveira et al. | 2006 | 55 | 100 | 45 | 45 | 0 | 50 |
| 24 | de Oliveira et al. | 2013 | 55 | 100 | 45 | 45 | 0 | 50 |
| 25 | Gacsályi | 2017 | 55 | 95 | 40 | 40 | 0 | NR |
| 26 | Glover and Davis | 2008 | 60 | 95 | 35 | 35 | 0 | 50 |
| 27 | Guscott et al. | 2000 | 55 | 95 or 100 | 40 | 45 | 5 | 50 |
| 28 | Hebb et al. | 2003 | 50 | 100 | 50 | 50 | 0 | 10 |
| 29 | Helton et al. | 1998 | NR | 110 | NR | NR | NR | 50 |
| 30 | Hijzen and Slangen | 1989 | 51 | 105 / 110 | 54 | 59 | 5 | NR |
| 31 | Hijzen et al. | 1995 | 55 | 95 / 100 / 110 | 40 | 55 | 15 | 50 |
| 32 | Jenck et al. | 2000 | NR | 90 / 95 / 105 | NR | NR | NR | 50 |
| 33 | Johnson et al. | 2003 | NR | NR | NR | NR | NR | NR |
| 34 | Johnson et al. | 2005 | NR | 120 | NR | NR | NR | 50 |
| 35 | Joordens et al. | 1996 | 65 | 105 / 110 / 115 | 40 | 50 | 10 | 50 |
| 36 | Joordens et al. | 1997 | 78 | 118 | 40 | 40 | 0 | 25 |
| 37 | Joordens et al. | 1998 | 65 | 105 / 110 / 115 | 40 | 50 | 10 | 50 |
| 38 | Josselyn et al. | 1995 | 70 | 119 | 49 | 49 | 0 | NR |
| 39 | Li et al. | 2015 | NR | 100 | NR | NR | NR | 40 |
| 40 | Lu et al. | 2011 | NR | 105 | NR | NR | NR | NR |
| 41 | Mansbach and Geyer | 1988 | NR | 97 / 108 / 118 | NR | NR | NR | NR |
| 42 | Martin et al. | 2002 | 68 | 90 / 95 / 105 | 22 | 37 | 15 | 50 |
| 43 | Merali et al. | 2006 | 50 | 110 | 60 | 60 | 0 | NR |
| 44 | Missig et al. | 2010 | 65 | 95 / 105 / 115 | 30 | 50 | 20 | NR |
| 45 | Muthuraju et al. | 2014 | 55 | 100 | 45 | 45 | 0 | 50 |
| 46 | Myers et al. | 2004 | 60 | 95 / 100 / 105 | 35 | 45 | 10 | 50 |
| 47 | Nevins and Anthony | 1994 | 80 | 110 | 30 | 30 | 0 | NR |
| 48 | Pietraszek et al. | 2005 | 64 | 95 / 100 / 105 | 31 | 41 | 10 | 50 |
| 49 | Risbrough et al. | 2003 | 65 | 100 | 35 | 35 | 0 | 40 |
| 50 | Risbrough and Geyer | 2005 | 65 | 100 | 35 | 35 | 0 | 40 |
| 51 | Risbrough et al. | 2009 | 65 | 100 / 105 | 35 | 40 | 5 | 40 |
| 52 | Roppe et al. | 2004a | NR | NR | NR | NR | NR | NR |
| 53 | Roppe et al. | 2004b | NR | NR | NR | NR | NR | NR |
| 54 | Rorick-Kehn et al. | 2007 | 70 | 110 | 40 | 40 | 0 | 50 |
| 55 | Schulz et al. | 2001 | 52 | 100 | 48 | 48 | 0 | 20 |
| 56 | Shilling and Feifel | 2008 | 65 | 95 | 30 | 30 | 0 | 40 |
| 57 | Steiner et al. | 2011 | 62 | 90 / 100 / 110 | 28 | 48 | 20 | 10 |
| 58 | Steiner et al. | 2012 | 62 | 90 / 100 /110 | 28 | 48 | 20 | 10 |
| 59 | Steiner et al. | 2013 | 62 | 90 / 100 / 110 | 28 | 48 | 20 | 10 |
| 60 | Tizzano et al. | 2002 | NR | 120 | NR | NR | NR | NR |
| 61 | Toufexis et al. | 2016 | 60 | 90 & 95 ^A^ | 30 or 35 ^A^ | 30 or 35 ^A^ | 0 | 50 |
| 62 | Vale and Green | 1996 | 60 | 100 | 40 | 40 | 0 | 500 |
| 63 | Varty et al. | 2008 | NR | 105 | NR | NR | NR | NR |
| 64 | Walker et al. | 2002 | 60 | NR | NR | NR | NR | 50 |
| 65 | Zhang and Li | 2016 | 70 | 105 | 35 | 35 | 0 | 40 |
| 66 | Zhao et al. | 2018a | 70 | 95 / 100 / 110 | 25 | 40 | 15 | 50 |
| 67 | Zhao et al. | 2018b | 70 | 95 / 100 / 110 | 25 | 40 | 15 | 50 |
| 68 | Zhao et al. | 2019 | 70 | 95 / 100 / 110 | 25 | 40 | 15 | 50 |
