# Supplementary material for: Pharmacological modulation of conditioned fear in the fear-potentiated startle test: a systematic review and meta-analysis of animal studies
Source: Psychopharmacology (Berl). 2023 Jan 18;240(11):2361–401. doi: 10.1007/s00213-022-06307-1 (PMC10593622; doi:10.1007/s00213-022-06307-1)
Supplement: Supplementary file 6 — Supplementary file6 (DOCX 101 KB) [file 213_2022_6307_MOESM6_ESM.docx]

**Pharmacological modulation of conditioned fear in the fear-potentiated startle test: a systematic review and meta-analysis of animal studies**

Psychopharmacology

Lucianne Groenink, P Monika Verdouw, Yulong Zhao, Freija ter Heegde, Kimberley E Wever, Elisabeth Y Bijlsma

Corresponding author: Lucianne Groenink, l.groenink@uu.nl

**Supplementary file 6**

**A.** Summary of key indicators of study quality

**B.** Quality indicators and study design per article

**Supplementary file 6A.** Summary of key indicators of study quality. Data are shown as percentage of the total number of included articles.


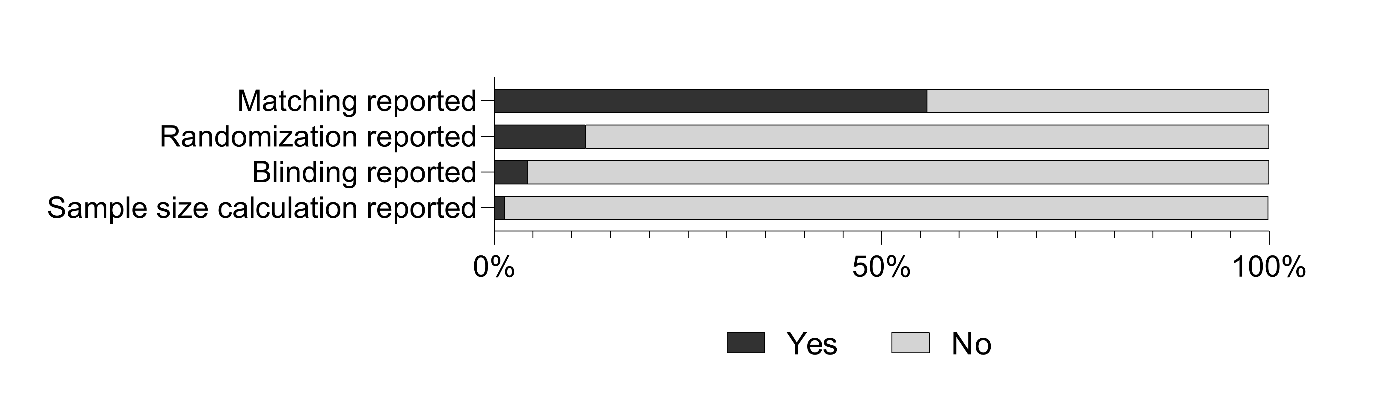


**Supplementary File** **6B.** Quality indicators and study design

| Article | Random allocation reported | Allocation based on matching; Procedure | Blinding reported | Sample size calculation reported | Study-design |
| --- | --- | --- | --- | --- | --- |
| Anthony and Nevins, 1993 | No | yes; post-training startle response (low and high groups) | no | no | Between subjects |
| Atack et al., 2006 | no | yes; pre-training startle response | no | no | Between subjects |
| Atack et al., 2011 | no | NR | no | no | NR |
| Ayers et al., 2011 | no | yes; pre-training startle response | no | no | Between subjects |
| Ayers et al., 2016 | no | yes; pre-training startle response | no | no | Between subjects |
| Bijlsma et al., 2010 | no | no | no | no | Within subjects (Latin square) |
| Bijlsma et al., 2015 | no | no | no | no | Within subjects (Latin square) |
| Bill et al., 1992 | yes | no; random allocation | yes | no | Between subjects |
| Brodkin et al., 2002 | no | no | no | no | Between subjects |
| Busse et al., 2004 | no | no | no | no | Between subjects |
| Caracache et al., 2011 | no | no | no | no | Between subjects |
| Cassella and Davis, 1985 | no | yes; pre-training startle response | no | no | Between subjects |
| Chen et al., 1997 | no | yes; pre-training startle response | no | no | Between subjects |
| Chhatwal et al., 2005 | no | yes; post training percent FPS | no | no | Between subjects |
| Chi, 1965 | yes | no; random allocation | no | no | Between subjects |
| Collado et al., 2002 | no | yes; pre-training startle response | no | no | NR |
| Collado et al., 2004 | no | NR | no | no | NR |
| Cosford et al., 2003 | no | NR | no | no | NR |
| Davis, 1979a | no | yes; pre-training startle response | no | no | Between subjects |
| Davis, 1979b | no | yes; pre-training startle response | no | no | Between subjects |
| Davis et al., 1979c | no | yes; pre-training startle response | no | no | Between subjects |
| Davis 1988a | no | yes; pre-training startle response | no | no | Between subjects |
| de Oliveira et al., 2006 | no | yes; pre-training startle response | no | no | Between subjects |
| de Oliveira et al., 2013 | no | yes; pre-training startle response | no | no | Between subjects |
| Gacsályi et al., 2017 | no | no | no | no | Between subjects |
| Glover and Davis, 2008 | no | yes; pre-training startle response | no | no | Between subjects |
| Guscott et al., 2000 | no | yes; pre-training startle response | no | no | Between subjects |
| Hebb et al., 2003 | no | no | no | no | Between subjects |
| Helton et al., 1998 | no | no | no | no | Between subjects |
| Hijzen and Slangen, 1989 | no | yes; pre-training startle response | no | no | Between subjects |
| Hijzen et al., 1995 | no | no; pre-training startle response as covariate | no | no | Within subjects (Latin square) |
| Jenck et al., 2000 | no | NR | no | no | Between subjects |
| Johnson et al., 2003 | no | NR | no | no | NR |
| Johnson et al., 2005 | yes | yes; pre-training startle response | no | no | Between subjects |
| Joordens et al., 1996 | no | yes; pre-training startle response | no | no | Within subjects (Latin square) |
| Joordens et al., 1997 | yes | no | no | no | Between subjects |
| Joordens et al., 1998 | no | yes; pre-training startle response^1^ | no | no | Within subjects (Latin square) |
| Josselyn et al., 1995 | no | yes; pre-training startle response | no | no | Between subjects |
| Li et al., 2015 | no | yes; pre-training startle response | no | no | Between subjects |
| Lu et al., 2011 | no | yes; post training percent FPS and startle response | no | no | Between subjects |
| Mansbach and Geyer, 1988 | no | no | no | no | Between subjects |
| Martin et al., 2002 | no | no | no | no | Between subjects |
| Merali et al., 2006 | no | yes; post training percent FPS | no | no | Between subjects |
| Missig et al., 2010 | no | yes; pre-training startle response | no | no | Between subjects |
| Muthuraju, 2014 | no | yes; pre-training startle respons | no | no | Between subjects |
| Myers et al., 2004 | no | yes; pre-training startle response | no | no | Between subjects |
| Nevins and Anthony, 1994 | no | yes; post training low high startle response groups | no | no | Between subjects |
| Pietraszek et al., 2005 | no | yes; pre-training startle response | no | no | Between subjects |
| Risbrough et al., 2003 | no | yes; pre-training startle response | no | no | Between subjects |
| Risbrough and Geyer, 2005 | no | yes; pre-training startle response | no | no | Between subjects |
| Risbrough et al., 2009 | no | yes; pre-training startle response | no | no | Between subjects |
| Roppe et al., 2004a | no | NR | no | no | NR |
| Roppe et al., 2004b | no | NR | no | no | NR |
| Rorick-Kehn et al., 2007 | no | no | no | no | Between subjects |
| Schulz et al., 2001 | yes | no | no | no | Between subjects |
| Shilling and Feifel, 2008 | no | yes; pre-training startle response | no | no | Between subjects |
| Steiner et al., 2011 | no | no | no | no | Within subjects (Latin square) |
| Steiner et al., 2012 | yes | no | no | no | Within subjects (Latin square) |
| Steiner et al., 2013 | no | NR | no | no | Within subjects (Latin square) |
| Tizzano et al., 2002 | no | yes; pre-training startle response | no | no | Between subjects |
| Toufexis et al., 2016 | no | yes; pre-training startle response | no | no | Between subjects |
| Vale and Green, 1996 | no | no | no | no | Between subjects |
| Varty et al., 2008 | no | yes; Post-training percent FPS | no | no | Between subjects |
| Walker et al., 2002, | no | yes; Pretraining startle response | no | no | Between subjects |
| Zhang and Li, 2016 | yes | no; Random allocation | no | no | Between subjects |
| Zhao et al.,2018a | yes | no; Random allocation | yes, blinded | no | Between subjects |
| Zhao et al., 2018b | no | no | yes, not blinded | yes | Within subjects (Latin square) |
| Zhao et al., 2019 | yes | no; Random allocation | no | no | Within subjects (Latin square) |

^1^ except for first 8-OH-DPAT experiment’; NR = not reported
