# Supplementary material for: Pharmacological modulation of conditioned fear in the fear-potentiated startle test: a systematic review and meta-analysis of animal studies
Source: Psychopharmacology (Berl). 2023 Jan 18;240(11):2361–401. doi: 10.1007/s00213-022-06307-1 (PMC10593622; doi:10.1007/s00213-022-06307-1)

**Pharmacological modulation of conditioned fear in the fear-potentiated startle test: a systematic review and meta-analysis of animal studies**

Psychopharmacology

Lucianne Groenink, P Monika Verdouw, Yulong Zhao, Freija ter Heegde, Kimberley E Wever, Elisabeth Y Bijlsma

Corresponding author: Lucianne Groenink, l.groenink@uu.nl

**Supplementary file 7**

Funnel plots for the effect of benzodiazepines on fear potentiation and on the non-cued baseline startle response.

Supplementary file 7. Funnel plots for the effect of benzodiazepines on fear potentiation (A) and on the non-cued baseline startle response (B).


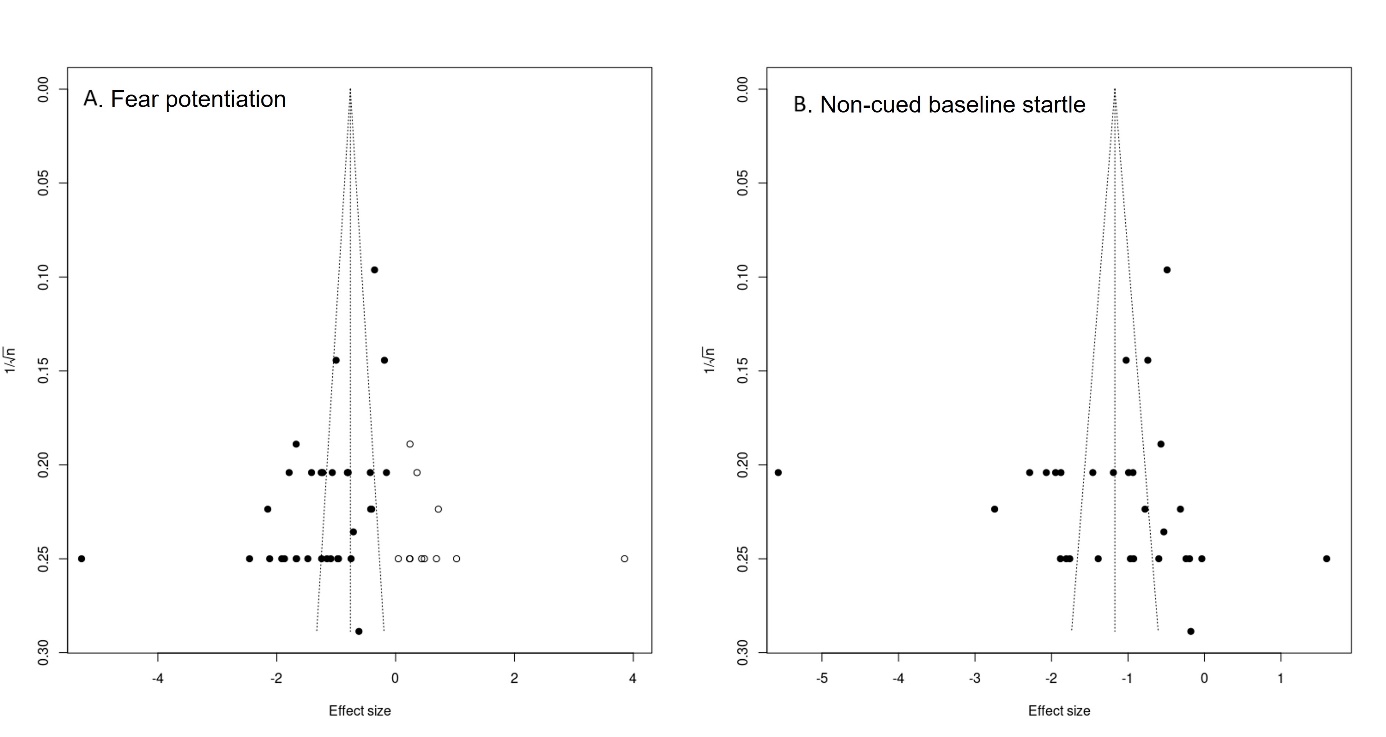

Supplement: Supplementary file 7 — Supplementary file7 (DOCX 111 KB) [file 213_2022_6307_MOESM7_ESM.docx]
