# Supplementary material for: Pharmacological modulation of conditioned fear in the fear-potentiated startle test: a systematic review and meta-analysis of animal studies
Source: Psychopharmacology (Berl). 2023 Jan 18;240(11):2361–401. doi: 10.1007/s00213-022-06307-1 (PMC10593622; doi:10.1007/s00213-022-06307-1)
Supplement: Supplementary file 8 — Supplementary file8 (DOCX 112 KB) [file 213_2022_6307_MOESM8_ESM.docx]

**Pharmacological modulation of conditioned fear in the fear-potentiated startle test: a systematic review and meta-analysis of animal studies**

Psychopharmacology

Lucianne Groenink, P Monika Verdouw, Yulong Zhao, Freija ter Heegde, Kimberley E Wever, Elisabeth Y Bijlsma

Corresponding author: Lucianne Groenink, l.groenink@uu.nl

**Supplementary File 8.** Description and discussion of methodological characteristics, other than those predefined for subgroup analysis.

**Supplementary File 8.** Description and discussion of methodological characteristics, other than those predefined for subgroup analysis.

**Description of methodological characteristics**

*Acclimation*

In thirty-eight articles animals were familiarized to the testing conditions before they were exposed to the fear conditioning procedure. In 37 articles this included exposure to both the test chamber and startle probes, whereas in one article animals were placed in the test chamber but not exposed to startle-eliciting probes. In 8 out of the 38 articles exposure to the chamber was repeated one or two times (Table 2, Supplementary file 4, Table A).

Characteristics of the presented startle probes varied. Animals were exposed to either one (22 articles) or more varying startle probe intensities (15 articles). In most cases (22 of 36 articles) similar noise intensities were used during acclimation and testing.

In thirteen articles, animals were not pre-exposed to the test chambers and startle probes before they entered the fear conditioning procedure. In another 17 articles it was not mentioned if animals had been pre-exposed to the test conditions.

*Acquisition training*

For the acquisition training a variety of protocols was used. Protocols mostly varied in the (total) number of cue - shock pairings presented, foot-shock intensity used, (total) number of training sessions, and number of training days. Duration of foot-shock, duration of cue light presentation and the timing of foot-shock relative to cue light presentation were relatively similar between articles (Table 2, Supplementary File S4, Table B).

In 58 articles, animals received one fear conditioning training per day. Typically, such a training was performed on two consecutive days (37 articles), although a one-day training (18 articles), and a three- (2 articles) or four-day training (1 article) protocols were also reported. The three other articles that reported this information trained the animals two times per day for two consecutive days (Steiner et al., 2011, 2012, 2013). In 7 articles, no information on the number of training sessions was reported.

Almost all articles that reported on foot-shock duration delivered a shock of 500 ms duration (55 out of 60 articles), and shock delivery co-terminated with the cue light presentation, except for two articles (Brodkin et al., 2002; Chi, 1965). In these two articles, also a substantially longer cue light duration was used (10 s and 7.5 s respectively). The four articles in which mice were tested also described the use of a considerably longer cue light duration (30s), whereas foot-shock was delivered during the last 500 (or 250) ms of the cue-light just as in most of the rat studies.

In most articles the cue light was presented between 3000 and 4000 ms (42 articles), with 3700 ms being the most frequently used duration (28 articles). The shortest cue light duration used was 1000 ms. The 1000 ms cue light duration was reported in four articles, three of which also used the highest number of cue-shock pairings (45 parings; (Michael Davis, 1979; Michael Davis et al., 1979; Hijzen & Slangen, 1989).

Both fixed (16 articles) and variable (40 articles) inter-trial intervals were used between the cue-shock pairings (11 articles did not report this information). The average inter-trial interval used varied between 10 s and 6 min. Variable intervals seemed more often combined with longer inter-trial interval durations (the 90 s and longer), but this was not a consistent pattern (Table 2, Supplementary File S4, Table B).

*Fear-potentiated startle test*

*General characteristics*

The time between fear conditioning and the fear-potentiated startle test was reported in 59 articles. This period ranged from 3 to 144 hours, with 24 h being the most frequently used interval (41/59 articles; Table 2). In 41 articles training and testing were conducted in the same context, in nine articles it was explicitly mentioned that the context in which animals received training was different from the context in which they were tested. In one article context was included as experimental factor in the study design (Zhao, Bijlsma, Verdouw, Garssen, et al., 2018). In the remaining 17 articles this information was not reported.

Background noise in the startle chambers varied between 45 to 80 dB. In the majority of the articles (37 articles), the reported background noise was between 55 and 70 dB. In twenty of the 68 articles the intensity of background noise was not reported (Supplementary Table 3).

Forty-eight articles reported that the test session started with a brief adaptation period. The time for adaptation varied from three to ten minutes, with five minutes being most frequently used (44/48). Besides this adaptation to the test equipment, 39 articles reported on the presentation of startle probes to habituate animals to the probes presented during the actual test. In 22 articles animals were not habituated to the startle probes and 7 articles did not report on habituation trials. Thirty articles reported the use of both adaptation time and habituation trials.

Thirty-four out of these 39 articles used one single intensity for habituation trials. In 24 articles this intensity was similar to the intensity of the test trials. The total number of habituation trials used ranged from 4 to 30 (Table 2, Supplementary File S4 Table C).

*Characteristics of startle trials*

The number of cued and non-cued trials presented to an animal within one test session ranged from 5 each to 40 each, with 30 trials being most frequently reported (16 articles). Nine articles did not report how many trials were presented. Three articles used separate experimental groups to compare the startle response to cued and non-cued trials (Chi, 1965; Hebb et al., 2003; Joordens et al., 1997).

Of the articles that measured the startle response to cued and non-cued trials within a session (65/ 68) 47 reported the order of trial presentation (Table 2, Supplementary File S4, Table C). Nine articles used a random presentation of trial types within a session, 36 articles applied a pseudo-random order, meaning that the total number of trials was split into equal blocks, and within each block, all trials are presented at random. Two articles alternated the cued and non-cued trials, and in one article the different trial types were presented separately (Shilling & Feifel, 2008). First, all trials of one type were presented, followed by all trials of the other type. This order was counterbalanced within treatment conditions.

In 36 articles a fixed inter-trial interval was used, whereas in 16 articles a variable inter-trial interval was used. The interval duration ranged from 8 s to 150 s. An inter-trial interval of 30 s was most frequently reported (35/52 articles; Table 2). In only five articles an inter-trial interval of 60 s or more was used. Three of these articles used mice as subjects. Sixteen articles did not report any information on the inter-trial interval.

In most articles, noise duration was 50 ms (29 of the 50 articles that reported this value; Supplementary File S4, Table D). Based on the articles that reported both background noise and probe intensities (49 articles), we calculated that the startle probe intensity varied between 21 and 65 dB above background (in absolute values the lowest intensity used was 85 dB, and the highest intensity 122 dB (Supplementary File S4, Table E).

Thirty nine of the 68 articles reported details on the time at which the startle probe was presented relative to the cue light presentation. In most articles (36/39), the startle probe was timed at the same moment as the foot-shock was switched on during training. In the other three articles, the startle probe was presented at the end of the time window in which the foot-shock was presented during the training (Ayers et al., 2016; Chen et al., 1997; Mansbach & Geyer, 1988).

**Discussion**

*4.2.1 Animal characteristics*

*Species differences*

It was not possible to make any reasonable comparisons between species, because only four of the included articles reported on experiments in which mice were. In addition, the conditioning procedures used for mice differed markedly from those used for rats. Most distinct difference related to the long duration of cue light presentation, up to 30 s. The use of specific characteristics for the mouse fear-potentiated startle has been well addressed by Falls (Falls, 2002).

*Sex differences*

Data were insufficient to conduct a meta-analysis on sex differences. Individual studies showed conflicting results with either no sex differences (Zhao, Bijlsma, Verdouw, & Groenink, 2018) or stronger fear-potentiation in female rats (de Jongh et al., 2005; Toufexis et al., 2016). The recommendations and directives to include female subjects in pharmacological studies (McCullough et al., 2014; Sandberg et al., 2015) will provide more conclusive evidence on the role of sex differences in the modulation of drug effects.

*Light-dark cycle*

The present review shows that most experiments were conducted during the light phase of the light-dark cycle, when the animals are resting. Data were too limited to analyse the impact of the phase of the light-dark cycle on drug actions in the fear-potentiated startle. Neurotransmitter systems (Jiménez-Zárate et al., 2021; Rueter & Jacobs, 1996), as well as animal behaviour (Roedel et al., 2006) are known to show light-dark variations. More specifically, the startle response in rats also varies depending on the phase of the light-dark cycle, with higher and more stable responses during the dark phase (Davis & Sollberger, 1971). It would therefore seem more accurate, also from a translational point of view to conduct the experiments during the dark phase, when animals are active.

**References**

Ayers, L., Agostini, A., Schulkin, J., & Rosen, J. B. (2016). Effects of oxytocin on background anxiety in rats with high or low baseline startle. *Psychopharmacology*, *233*(11), 2165–2172. https://doi.org/10.1007/s00213-016-4267-0

Brodkin, J., Busse, C., Sukoff, S. J., & Varney, M. A. (2002). Anxiolytic-like activity of the mGluR5 antagonist MPEP: A comparison with diazepam and buspirone. *Pharmacology Biochemistry and Behavior*, *73*(2), 359–366. https://doi.org/10.1016/S0091-3057(02)00828-6

Chen, Y. L., Mansbach, R. S., Winter, S. M., Brooks, E., Collins, J., Corman, M. L., Dunaiskis, A. R., Faraci, W. S., Gallaschun, R. J., Schmidt, A., & Schulz, D. W. (1997). *A Centrally Active Corticotropin-Releasing Factor 1 Receptor Antagonist*. *2623*(96), 1749–1754.

Chi, C. C. (1965). The effect of amobarbital sodium on conditioned fear as measured by the potentiated startle response in rats. *Psychopharmacologia*, *7*(2), 115–122. https://doi.org/10.1007/BF00403634

Davis, M. (1979). Morphine and Naloxone: Effects on Conditioned Fear as Measured With the Potentiated Startle Paradigm. *European Journal of Pharmacology*, *54*, 341–347.

Davis, M., Redmond, D. E., & Baraban, J. M. (1979). Noradrenergic agonists and antagonists: Effects on conditioned fear as measured by the potentiated startle paradigm. *Psychopharmacology*, *65*(2), 111–118. https://doi.org/10.1007/BF00433036

Davis, M., & Sollberger, A. (1971). Twenty-four-hour periodicity of the startle response in rats. *Psychonomic Science*, *25*(1), 37–39. https://doi.org/10.3758/BF03335842

de Jongh, R., Geyer, M. A., Olivier, B., & Groenink, L. (2005). The effects of sex and neonatal maternal separation on fear-potentiated and light-enhanced startle. *Behavioural Brain Research*, *161*(2). https://doi.org/10.1016/j.bbr.2005.02.004

Falls, W. A. (2002). Fear-potentiated startle in mice. *Current Protocols in Neuroscience*, *Chapter 8*. https://doi.org/10.1002/0471142301.NS0811BS19

Hebb, A. L., Zacharko, R. M., Bowie, J. P., & Drolet, G. (2003). Differential startle reactivity following central CCK-8S and systemic Boc CCK-4 administration in mice: antecedent stressor history and testing condition. *Behav Neurosci*, *117*(4), 704–715. https://doi.org/10.1037/0735-7044.117.4.704

Hijzen, T. H., & Slangen, J. L. (1989). Effects of midazolam, DMCM and lindane on potentiated startle in the rat. *Psychopharmacology*, *99*(3), 362–365.

Jiménez-Zárate, B. S., Piña-Leyva, C., Rodríguez-Sánchez, M., Florán-Garduño, B., Jiménez-Zamudio, L. A., & Jiménez-Estrada, I. (2021). Day-Night Variations in the Concentration of Neurotransmitters in the Rat Lumbar Spinal Cord. *Journal of Circadian Rhythms*, *19*(1), 1–8. https://doi.org/10.5334/JCR.215

Joordens, R. J. E., Hijzen, T. H., Peeters, B. W. M. M., & Olivier, B. (1997). Control conditions in the fear-potentiated startle response paradigm. *NeuroReport*, *8*(4), 1031–1034. https://doi.org/10.1097/00001756-199703030-00042

Mansbach, R. S., & Geyer, M. A. (1988). Blockade of potentiated startle responding in rats by 5-hydroxytryptamine1A receptor ligands. *European Journal of Pharmacology*, *156*(3), 375–383. https://doi.org/10.1016/0014-2999(88)90283-X

McCullough, L. D., de Vries, G. J., Miller, V. M., Becker, J. B., Sandberg, K., & McCarthy, M. M. (2014). NIH initiative to balance sex of animals in preclinical studies: generative questions to guide policy, implementation, and metrics. *Biology of Sex Differences*, *5*(1). https://doi.org/10.1186/S13293-014-0015-5

Roedel, A., Storch, C., Holsboer, F., & Ohl, F. (2006). Effects of light or dark phase testing on behavioural and cognitive performance in DBA mice. *Laboratory Animals*, *40*(4), 371–381. https://doi.org/10.1258/002367706778476343

Rueter, L. E., & Jacobs, B. L. (1996). Changes in forebrain serotonin at the light-dark transition: correlation with behaviour. *Neuroreport*, *7*(5), 1107–1111. https://doi.org/10.1097/00001756-199604100-00031

Sandberg, K., Umans, J. G., Berga, S. L., Bonham, A. C., Clarke, R., Darsow, T., Deschamps, A. M., Goodman, J. L., Gore, A. C., Howard, B. v., Karty, A., Mann, J., McCarthy, M. M., Mellman, T. A., Miller, V. M., Molina, P. E., Morris, P. L., Northcott, C. A., O’Brien, E. R. M., … Woolley, C. S. (2015). Recommendations concerning the new U.S. National Institutes of Health initiative to balance the sex of cells and animals in preclinical research. *FASEB Journal : Official Publication of the Federation of American Societies for Experimental Biology*, *29*(5), 1646–1652. https://doi.org/10.1096/FJ.14-269548

Shilling, P. D., & Feifel, D. (2008). The neurotensin-1 receptor agonist PD149163 blocks fear-potentiated startle. *Pharmacology Biochemistry and Behavior*, *90*(4), 748–752. https://doi.org/10.1016/j.pbb.2008.05.025

Steiner, M. A., Gatfield, J., Brisbare-Roch, C., Dietrich, H., Treiber, A., Jenck, F., & Boss, Christoph. (2013). Discovery and Characterization of ACT-335827, an Orally Available, Brain Penetrant Orexin Receptor Type 1 Selective Antagonist. *ChemMedChem*, *8*(6), 898–903. https://doi.org/10.1002/cmdc.201300003

Steiner, M. A., Lecourt, H., & Jenck, F. (2012). The brain orexin system and almorexant in fear-conditioned startle reactions in the rat. *Psychopharmacology*, *223*(4), 465–475. https://doi.org/10.1007/s00213-012-2736-7

Steiner, M. A., Lecourt, H., Rakotoariniaina, A., & Jenck, F. (2011). Favoured genetic background for testing anxiolytics in the fear-potentiated and light-enhanced startle paradigms in the rat. *Behavioural Brain Research*, *221*(1), 34–42. https://doi.org/10.1016/J.BBR.2011.02.021

Toufexis, D. J., Lipatova, O., Johnson, A. C., & Abizaid, A. (2016). Food-Restriction Lowers the Acoustic Startle Response in both Male and Female Rats, and, in Combination with Acute Ghrelin Injection, Abolishes the Expression of Fear-Potentiated Startle in Male Rats. *Journal of Neuroendocrinology*, *28*(11). https://doi.org/10.1111/jne.12436

Zhao, Y., Bijlsma, E. Y., Verdouw, M. P., & Groenink, L. (2018). No effect of sex and estrous cycle on the fear potentiated startle response in rats. *Behavioural Brain Research*, *351*, 24–33. https://doi.org/10.1016/J.BBR.2018.05.022

Zhao, Y., Bijlsma, E. Y., Verdouw, P. M., Garssen, J., & Groenink, L. (2018). The contribution of contextual fear in the anxiolytic effect of chlordiazepoxide in the fear-potentiated startle test. *Behavioural Brain Research*, *353*, 57–61. https://doi.org/10.1016/j.bbr.2018.06.035
